# Elucidating the Inhibitory Effect of Resveratrol and Its Structural Analogs on Selected Nucleotide-Related Enzymes

**DOI:** 10.3390/biom10091223

**Published:** 2020-08-22

**Authors:** Yifei Wu, Tze-chen Hsieh, Joseph M. Wu, Xiaoxiao Wang, Joshua S. Christopher, Amanda H. Pham, Justin David-Li Swaby, Lei Lou, Zhong-Ru Xie

**Affiliations:** 1 Computational Drug Discovery Laboratory, School of Electrical and Computer Engineering, College of Engineering, University of Georgia, Athens, GA 30602, USA; wuyifei@uga.edu (Y.W.); jsc04581@uga.edu (J.S.C.); amandahuongpham@gmail.com (A.H.P.); jds67892@uga.edu (J.D.-L.S.); lei.lou@uga.edu (L.L.); 2 Department of Biochemistry & Molecular Biology, New York Medical College, Valhalla, NY 10595, USA; tze-chen_hsieh@nymc.edu (T.-c.H.); Joseph_Wu@nymc.edu (J.M.W.); drwangx2008@gmail.com (X.W.); 3 The Franklin College of Arts and Sciences, University of Georgia, Athens, GA 30602, USA

**Keywords:** resveratrol, inhibition mechanism, nucleotide-related enzymes, molecular dynamics simulation, molecular docking

## Abstract

Resveratrol, the most widely studied natural phytochemical, has been shown to interact with different target proteins. Previous studies show that resveratrol binds and inhibits DNA polymerases and some other enzymes; however, the binding and functioning mechanisms remain unknown. The elucidated knowledge of inhibitory mechanisms of resveratrol will assist us in new drug discovery. We utilized molecular docking and molecular dynamics (MD) simulation to reveal how resveratrol and structurally similar compounds bind to various nucleotide-dependent enzymes, specifically, DNA polymerases, HIV-1 reverse transcriptase, and ribonucleotide reductase. The results show that resveratrol and its analogs exert their inhibitory effects by competing with the substrate dNTPs in these enzymes and blocking elongation of chain polymerization. In addition, the results imply that resveratrol binds to a variety of other ATP-/NTP-binding proteins.

## 1. Introduction

Anti-proliferative and antiviral activities are among the most extensively studied properties of resveratrol (3,4${}^{\prime }$,5-trihydroxystilbene) [1,2,3,4,5], which has demonstrated nutraceutical and potentially therapeutic activities in a large number of studies [6,7,8,9]. The distinct functions of resveratrol are in part attributed to its regulatory activity on signaling pathways and its impacts on molecular targets [10]. For instance, resveratrol inhibits the replication of viruses, thus interfering with viral infection, which is critical to the prevention and the treatment of viral diseases, such as AIDS [11]. Resveratrol has been shown to inhibit DNA polymerase α ($K_{i}$ = 3.3 μM), DNA polymerase δ ($K_{i}$ = 5 μM), and ribonucleotide reductase (IC_50_ = 100 μM) [12,13], which are crucial enzymes for DNA related metabolic processes, whose abnormalities may cause cancer or aging diseases [14,15,16]. However, the mechanisms of inhibition of DNA polymerases and ribonucleotide reductase remain unclear [12,17].

The structure of resveratrol is a prominent feature that governs its biological activity. *Trans*-resveratrol has one *para*-hydroxyl group and two *meta*-hydroxyl groups [18]. Among three hydroxyl groups, the *para*-hydroxyl group shows major reactivity against the oxidative damage and thus may play a primary role on how resveratrol acts as a deterrent to various diseases caused by free radicals such as cancer and Alzheimer’s [19,20,21]. Of note, the structural similarity between resveratrol and purine/pyrimidine bases may contribute to its pronounced inhibitory effects on nucleotide-dependent cellular processes and specific enzymes.

Previous studies have shown that resveratrol bears structural resemblance to adenosine triphosphate (ATP) and cyclic adenosine monophosphate (cAMP), which provide a molecular and functional mechanism for its inhibition of mTOR by occupying the binding site of ATP [22]. Similarly, resveratrol has also been shown by enzyme kinetics to inhibit protein kinase CKII by competing with ATP [23]. Because ATP is an essential building block for RNA or DNA synthesis, we hypothesize that resveratrol modulates DNA replication or RNA transcription by competing with nucleotides for the binding sites of pertinent enzymes, specifically, DNA polymerases and ribonucleotide reductase. Moreover, a similar consideration could be applied to the other nucleotide-related enzymes such as reverse transcriptase and RNA polymerase, i.e., their inhibition by resveratrol operates using a substrate competition mechanism.

To test our hypothesis, we selected three enzymes, namely, DNA polymerase α, HIV-1 reverse transcriptase, and ribonucleotide reductase, as our targets. The overarching goal of our study was to test and demonstrate the mechanism of inhibition of resveratrol. To this end, we modeled the interaction between resveratrol and nucleotide-related enzymes using protein-ligand docking. We used molecular docking to test the effects of resveratrol and several structural analogs for competition with representative nucleotides on the substrate binding site of the three selected enzymes. The results show that resveratrol and several of its analogs display steric clashes against the nucleotides for occupancy on the binding site, which could explain the inhibition mechanism of resveratrol on the nucleotide-related enzymes.

## 2. Materials and Methods

### 2.1. Protein Structure Preparation

The associated PDB files of DNA polymerase α (PDB ID: 4Q5V), ribonucleotide reductase (PDB ID: 5TUS), and HIV-1 reverse transcriptase (PDB ID: 5TXM) were downloaded from RCSB’s Protein Data Bank (PDB) [24]. To obtain the structure of DNA polymerase δ, we first downloaded the DNA sequence of human DNA polymerase δ from the UniProt website (uniprot ID: P28340) [25,26] and the protein structure of *Saccharomyces cerevisiae* DNA polymerase δ (PDB ID:3IAY) as the template from PDB [24,27]. The DNA sequence of human DNA polymerase δ and 3IAY was uploaded to the SWISS-Model server to generate the predicted structure of human DNA polymerase δ. The DNA molecule in the predicted structure was extracted from 3IAY.

The protein structures used for docking were processed by the protein preparation wizard in Maestro (11.5 version, Schrodinger) [28]. The workflow of protein preparation consists of the following three steps: (1) Preprocessing: assigning bond orders, adding hydrogens, creating zero-order bonds to metals, creating disulfide bridges, filling in missing side chains using Prime, deleting waters beyond 5.00 Å from het groups, and generating het states using Epik (pH = 7.0 ± 2.0) [29]; (2) Optimization: setting pH = 7.0 and performing optimization; and (3) Minimization: this step was performed using the OPLS3e force field [30]. The converged heavy atoms to root-mean-square deviation (RMSD) is 0.30 Å.

### 2.2. Ligand Preparation

The 3-D molecular structures of *trans*-resveratrol, *cis*-resveratrol, pterostilbene, piceatannol, quercetin, myricetin, mulberroside A, pinostilbene, and hydroxyurea were obtained from the PubChem database. The 3-D molecular structures of resveratrol analogs: 3,3${}^{\prime }$,4,4${}^{\prime }$,5,5${}^{\prime }$-hexahydroxystilbene (M8), *trans*-3,5-dihydroxy-4${}^{\prime }$-methoxystilbene (DRG), *trans*-4,4${}^{\prime }$-dihydroxystilbene (4,4${}^{\prime }$-DHS), 3${}^{\prime }$-hydroxypterostilbene (HPSB), *trans*-3${}^{\prime }$,4${}^{\prime }$,5${}^{\prime }$-trihydroxystilbene (3,4,5-THS), (E)-5-(4-Nitrostyryl) benzo[d][1,3]dioxole (Compound-1), (E)-5-(4-Nitrostyryl)-1,3-phenylene diacetate (Compound-2), and (E)-4-(2,3,4-Trimethoxystyryl)benzenamine (Compound-3) were built in Maestro (version 11.5, Schrodinger) based on a previous study [5,17,18,31]. All the compounds were prepared using OPLS3e force field in Ligprep panel in Maestro [30]. The preparation process included converting 2D structures to 3D ones, adding hydrogens, computing correct partial charges, and optimizing the structures.

### 2.3. Ligand-Protein Docking

To predict the details of interaction between ligands and the target proteins and to estimate their binding affinities (see section **MM-GBSA calculation**), ligand docking was conducted using the extra-precision (XP) mode in Maestro. After the ligands and the target proteins were processed using Ligprep and protein preparation, respectively, a receptor grid box was generated according to the binding sites of existing ligands such as dNTP. For the predicted protein structure or the structure without an existing ligand, we would let the receptor grid box come close to the 3${}^{\prime }$-end of the DNA/RNA molecule. The size of the receptor grid box was set as default (20 Å). Then, the ligand-protein docking was performed using the XP mode.

### 2.4. MM-GBSA Calculation

The binding energy ($\Delta G_{\mathrm{bind}}$) between a protein and a ligand reflects how stably they bind to each other. Therefore, we examined whether an inhibitor tightly binds onto its target protein by calculating the MM-GBSA energies. Here, $\Delta G_{\mathrm{bind}}$ was estimated using the Prime MM-GBSA module in Maestro (As a default setting, the entropy term is neglected) [32]. In the MM-GBSA panel, the pose viewer files of docked complex were uploaded. The solvation model was VSGB, and the force field was OPLS3e [30]. Prime MM-GBSA $\Delta G_{\mathrm{bind}}$ was calculated using the following equation:

(1) $$\Delta G_{bind}=E_{complex}\left(minimized\right)-\left[E_{ligand}\left(minimized\right)+E_{receptor}\left(minimized\right)\right]$$

where $\Delta G_{\mathrm{Bind}}$ is binding free energy and $E_{complex}\left(minimized\right)$, $E_{ligand}\left(minimized\right)$ and $E_{receptor}\left(minimized\right)$ are minimized energies of receptor-ligand complex, ligand and receptor, respectively.

### 2.5. Molecular Dynamics Simulation

The MD simulations were performed using GROMACS version 2018.1 and CHARMM36 all-atom force field (March 2019) [33,34,35,36,37]. Here, the complex of 4Q5V bound with *trans*-resveratrol and the complex of 4Q5V bound with dCTP were chosen to run MD simulation. The starting coordinates of the protein-ligand complex were obtained from docking experiments. Then, we defined a dodecahedral unit cell with diameter of 10.275 nm and volume of 2850.21 nm${}^{3}$, and filled it with water. After adding ions, the complex was minimized for 50,000 steps of steepest descent minimization. Next, the complex was equilibrated using an NVT ensemble (constant Number of particles, Volume, and Temperature) and NPT ensemble (the Number of particles, Pressure, and Temperature). The target temperature for equilibration was 300 K. The last step consisted of performing the stimulations for 30 ns. After the MD simulations, we calculated the minimum distance between the hydroxyl group and the residues (Leu864, Ash1004) for each ligand, that is, the distance between 3${}^{\prime }$-OH of dCTP and Ash1004, the distance between 3${}^{\prime }$-OH of dCTP and Leu864, the distance between 4${}^{\prime }$-OH of resveratrol and Ash1004, and the distance between 4${}^{\prime }$-OH of resveratrol and Leu864.

### 2.6. DNA Oligo Substrates Extension Assay

To test the inhibitory effects of the drugs on the replication activity of DNA polymerase δ, in vitro DNA oligo substrates extension assay was carried out, with concentration titration of the drugs at different time points. The template for primer extension consisted of a 40 mer: 5${}^{\prime }$-TCATCGGTCGCATCGCTGGCTGTCAAGGTGCTGTAGTGGC-3${}^{\prime }$, which was annealed with a 25 mer primer 5${}^{\prime }$-GCCACTACAGCACCTTGACAGCCAG-3${}^{\prime }$. The 5${}^{\prime }$ end of the 25 mer primer was labeled with gamma-32p-ATP. The reaction contained 100 nM of DNA, 100 nM PCNA, 4 nM DNA polymerase δ, 2, 5, 10, 20, or 30 μM of the drugs being tested where indicated, 50 mM Tris-HCl pH 7.5, 30 mM NaCl, 2 mM dithiothreitol, 0.1 mg/mL BSA, 5 mM MgCl${}_{2}$, and 20 μM dNTP. MgCl${}_{2}$ and dNTPs were added to the mixture to start the reactions, and equal volumes of gel loading buffer were added to stop the reactions. The loading buffer contained 50 mM EDTA, 95% formamide, 0.01% bromophenol blue, and 0.01% xylene cyanol. Reaction products were subjected to electrophoresis on sequencing gels (16–20% acrylamide/bisacrylamide 19:1 (Bio-Rad), 7.4 M urea, 1 mM EDTA, 90 mM Tris–HCl and 90 mM boric acid). Reaction products were visualized by phosphorimaging with a Molecular Dynamics Storm Phosphorimaging system and quantified with ImageQuant software (Amersham Biosciences).

## 3. Results

### 3.1. Inhibitory Effect of Resveratrol on DNA Polymerase α

**Figure 1:**
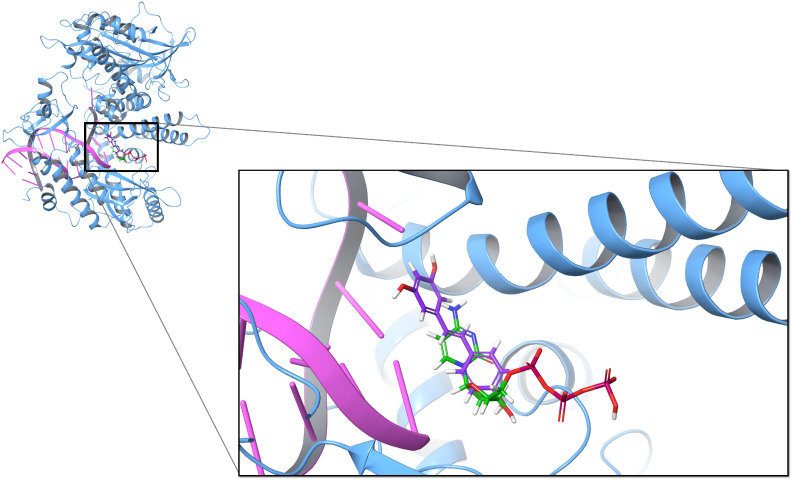
Superposition of two docking poses of *trans*-resveratrol (purple) and dCTP (green) on DNA polymerase α (PDB ID: 4Q5V).

To explore the inhibitory mechanism of resveratrol, we assessed the inhibitory effect of resveratrol on the dNTP binding process. On the DNA polymerase α structure (PDB ID: 4Q5V), dCTP is the incoming deoxynucleotide substrate that is more effectively inhibited by the natural diterpenoid aphidicolin compared to other dNTPs [38]. Docking dCTP onto its binding site on DNA polymerase α and calculating the MM-GBSA energy gives a score of −49.46 kcal/mol. The same docking approach using *trans*-resveratrol instead of dCTP results in an MM-GBSA energy score of −49.58 kcal/mol, showing that *trans*-resveratrol exhibits slightly better binding affinity than dCTP. This result suggests that *trans*-resveratrol preferentially binds to the dNTP binding site compared to dCTP. By overlapping two docking poses, we observed that *trans*-resveratrol binds to the same position as dCTP, which also demonstrated that *trans*-resveratrol could be used as a competing inhibitor for DNA polymerase α (Figure 1). Based on the previous docking results, we further docked dCTP onto the complex composed of 4Q5V bound with *trans*-resveratrol. We found that the binding position of dCTP was changed because *trans*-resveratrol occupied the original binding site. The MM-GBSA energy of dCTP decreased to −29.87 kcal/mol, which indicates that dCTP rarely binds to its original site because of the presence of *trans*-resveratrol.

**Figure 2:**
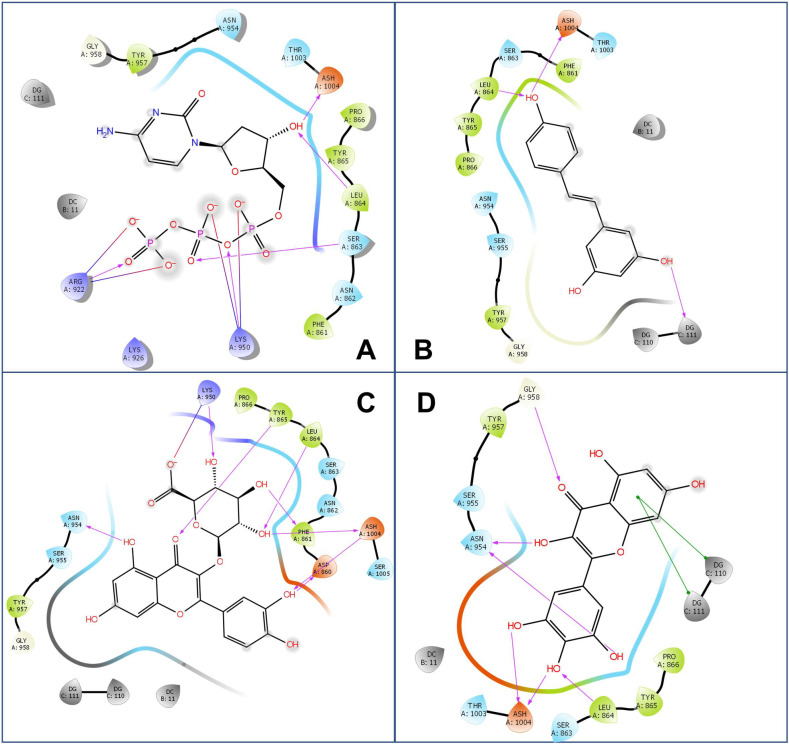
2-D Protein-ligand interaction diagrams of 4Q5V and four ligands: dCTP (**A**), *trans*-resveratrol (**B**), miquelianin (**C**), and myricetin (**D**). The purple arrow indicates the hydrogen bond; the green line represents π-π stacking.

Furthermore, to explore the inhibitory effects of resveratrol-related compounds, resveratrol analogs were individually examined as docking ligands onto the dCTP binding site. Calculation of the MM-GBSA of the 16 analogs tested shown in Table 1 revealed that miquelianin (quercetin 3-*O*-glucuronide) had the best binding affinity, giving a score of −73.53 kcal/mol. By comparing the 2-D protein-ligand interaction diagrams (Figure 2), we found that Leu864 and Ash1004 (protonated Asp, the pKa value of Ash1004 is 8.34 in the local environment, calculated by PROPKA Program in Maestro Protein Preparation Wizard [39]) are positioned for interaction with ligands via hydrogen bonds (formed by the delta oxygens on the side chain of Ash1004 and the oxygen or nitrogen on the main chain of Leu864), as illustrated by the interaction with the 4${}^{\prime }$-hydroxy groups of *trans*-resveratrol and 13 analogs except for DRG (*trans*-3,5-dihydroxy-4${}^{\prime }$-methoxystilbene), compound-1 ((E)-5-(4-Nitrostyryl)benzo[d][1,3]dioxole), and compound-2 ((E)-5-(4-Nitrostyryl)-1,3-phenylene diacetate) (Figure 2 and Figures S2–S5). For the ligands with a 3${}^{\prime }$-hydroxy group and a 4${}^{\prime }$-hydroxy group, such as M8, the 4${}^{\prime }$-hydroxy group also can form hydrogen bonds with Leu864 and Ash1004, and in addition, binding of M8 with the dCTP site is also anchored by the interaction of its 3${}^{\prime }$-hydroxy group with Asn954. In the cases of quercetin, myricetin and 3,4,5-THS, with two/three hydroxy groups at the 3${}^{\prime }$-, 4${}^{\prime }$-, and 5${}^{\prime }$-position, there is further interaction with Ash1004 through the 3${}^{\prime }$/5${}^{\prime }$-hydroxy group. This interaction might be related to the low MM-GBSA energies of quercetin, myricetin and 3,4,5-THS (−59.08, −61.90 and −63.64 kcal/mol). As a negative control, hydroxyurea, an oral chemotherapeutic drug, is docked onto dCTP binding site and shows the worst MM-GBSA energy (−20.25 kcal/mol).

**Figure 3:**
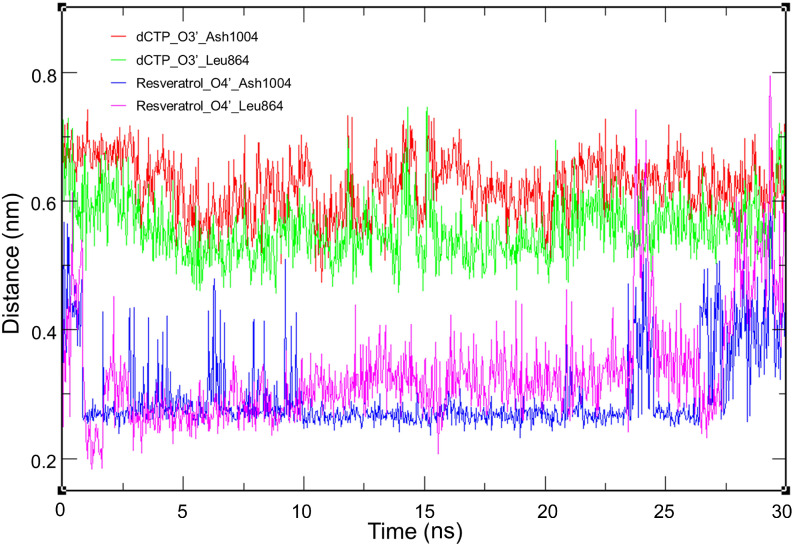
Minimum distance between hydrogen bond donors and acceptors on 4Q5V-ligand complex during MD simulation. The red line indicates the distance between 3${}^{\prime }$-OH of dCTP and Ash1004; the green line indicates the distance between 3${}^{\prime }$-OH of dCTP and Leu864; the blue line indicates the distance between 4${}^{\prime }$-OH of resveratrol and Ash1004; the pink line indicates the distance between 4${}^{\prime }$-OH of resveratrol and Leu864.

In the protein-ligand interaction diagram of dCTP, we observed that the 3${}^{\prime }$-OH forms two hydrogen bonds with Leu864 and Ash1004, suggesting that these two amino acid residues play a critical role in the binding site. Therefore, to further compare the interaction, we selected 4Q5V-bound *trans*-resveratrol and 4Q5V-bound dCTP as examples to run molecular dynamics (MD) simulation, and then calculated the distances between the hydroxyl group and the residues (Leu864, Ash1004) for each ligand. Consequently, we found that the distances of *trans*-resveratrol and two residues mainly fluctuated from 2 to 5 Å, and the distances of dCTP and two residues ranged from 4.5 to 7 Å (Figure 3). This range indicates that the hydrogen bonds are more likely to be formed between *trans*-resveratrol and two residues so that *trans*-resveratrol has strong competitiveness on binding to the dCTP binding site. Furthermore, the π-π stacking is another interaction between ligands and DNA molecules. For example, myricetin exhibits π-π stacking with dGTP110 and dGTP111 (Figure 2D). A previous report has shown that the 4${}^{\prime }$-hydroxy group is essential for the biological activities of resveratrol [18]. Therefore, 4,4${}^{\prime }$-DHS with only a 4${}^{\prime }$-hydroxy group shows relatively low MM-GBSA energy (−56.1 kcal/mol), whereas DRG with a 4${}^{\prime }$-methoxyl group has higher energy (−45.91 kcal/mol). Collectively, the results indicate that the ligand with three hydroxyl groups at the 3${}^{\prime }$-, 4${}^{\prime }$-, and 5${}^{\prime }$- (or 3-, 4-, and 5-) position, respectively, could bind closely to the binding site of DNA polymerase α.

**Figure 4:**
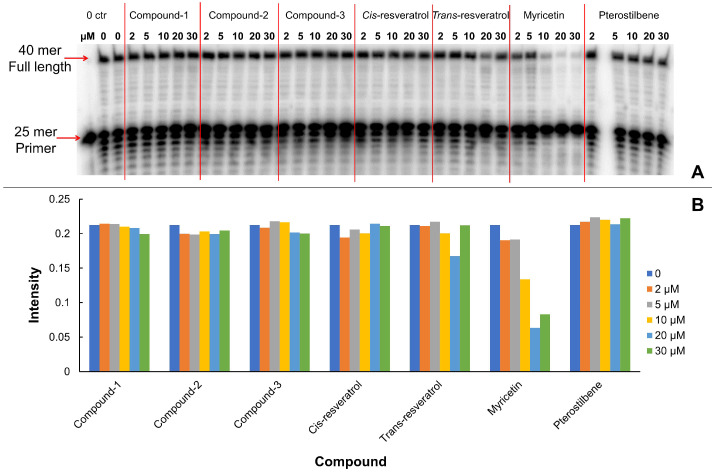
(**A**): Effects of increasing concentrations of resveratrol and its selected analogs on DNA oligo extension by human DNA polymerase δ. The first lane is the 25 mer primer, and the second and third lanes are the controls of the polymerase δ extension assay without addition of analogs. The inhibitors tested were compound-1, compound-2, compound-3, *cis*-resveratrol, *trans*-resveratrol, myricetin, and pterostilbene. The concentrations tested for each inhibitor were 2, 5, 10, 20, and 30 μM; (**B**) Bar chart of intensity of the full-length 40 mer products (as shown in **A**) by increasing concentrations of resveratrol and its selected analogs.

Additionally, we tested inhibitory effects of selected analogs of resveratrol on enzyme DNA polymerase δ. The inhibition of polymerase and exonuclease activities of DNA polymerase δ is clearly evident at concentrations of 10 μM or higher. Addition of increasing concentrations of myricetin at 10, 20 and 50 μM in the DNA 25 mer extension assay (Figure 4), the DNA oligo products ladders were almost diminished above and below the 25 mer primers, indicating that the primers were neither extended nor edited. The structure of human DNA polymerase δ has not been determined. We performed docking experiments on a predicted model of DNA polymerase δ, which was generated as we described in 2.1 protein protein structure preparation section. The docking results derived from DNA polymerase δ model (Table S1) showed results similar to those of DNA polymerase α; notably, however, the details of the molecular interactions were distinctively different. Since the predicted model may lack precision, we consider that the docking results attributed to DNA polymerase δ model to be less reliable than those generated using of DNA polymerase α. It is noteworthy that the validity of the docking approach is supported by experimental results using DNA polymerase α which has long been known to be potently inhibited by myricetin [40,41,42].

### 3.2. Inhibitory Effect on HIV-1 Reverse Transcriptase

**Figure 5:**
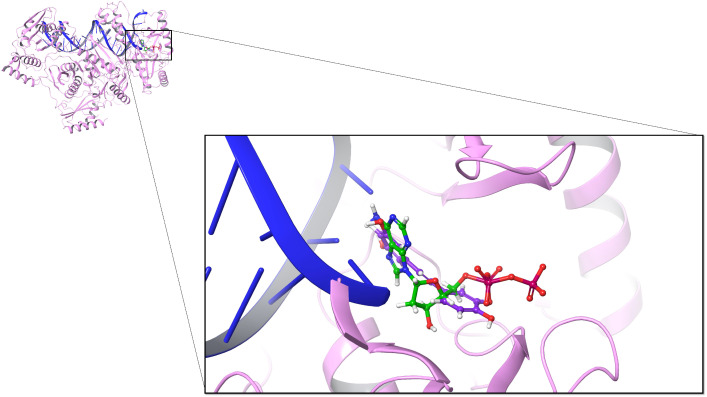
Superposition of two docking poses of *trans*-resveratrol (purple) and dATP (green) on HIV-1 reverse transcriptase (PDB ID: 5TXM).

To further examine our hypothesis that resveratrol could inhibit nucleotide-related enzymes, we also selected HIV-1 reverse transcriptase (PDB ID: 5TXM) as target [43]. First, we docked the original ligand ddATP and theoretical substrate dATP back onto the binding site separately. The MM-GBSA energies are −47.88 kcal/mol (ddATP) and −46.41 kcal/mol (dATP). Then, we docked *trans*-resveratrol onto the same binding site, and its MM-GBSA energy is −55.42 kcal/mol. The energy of *trans*-resveratrol is better than that of dATP, which indicated that *trans*-resveratrol can compete for the binding site of dATP. The docking poses (Figure 5) show that *trans*-resveratrol binds to the binding site of the dATP. That result validates our hypothesis that *trans*-resveratrol competes with substrate for the binding site. In addition, the binding pocket was not big enough to allow the existence of two ligands, so the substrate could not bind onto the binding site once *trans*-resveratrol occupies that position.

We also docked analogs of resveratrol onto the binding site of dATP (Table 1). The MM-GBSA energies of six resveratrol’s analogs are better than that of dATP, including compound-2, quercetin, myricetin, miquelianin, astringin, and mulberroside A. Specially, miquelianin shows the best binding energy than the other 18 compounds, and miquelianin and astringin show better binding affinities than that of *trans*-resveratrol. As a negative control, hydroxyurea shows the worst MM-GBSA energy of −10.82 kcal/mol. From the 2-D protein-ligand interaction diagrams (Figures S6–S10), we found that miquelianin and astringin have more interactions with binding site via hydrogen bond or π-π stacking than the other compounds, which could be the reason why miquelianin and astringin exhibit better binding affinities. In addition, through hydrogen bonding, the 4${}^{\prime }$-OH moiety of *trans*-resveratrol interacts with residues Ala114 and Asp185, and the 3-OH group interacts with dTTP on the DNA molecules. The compound DRG, which exhibits a substitution of a 4${}^{\prime }$-methoxyl group for a 4${}^{\prime }$-hydroxy group, has two hydrogen bonds on the 3-hydroxy group and the 5-hydroxy group. Similarly, pterostilbene, which has a 4${}^{\prime }$-hydroxy group and two methoxy groups on the 3- and the 5-position, forms two hydrogen bonds with Gly152 and Lys66. However, the MM-GBSA energies of DRG and pterostilbene are both worse than that of *trans*-resveratrol, which demonstrates that the hydroxyl groups of *trans*-resveratrol contribute to binding affinity.

### 3.3. Inhibitory Effect on Ribonucleotide Reductase

**Figure 6:**
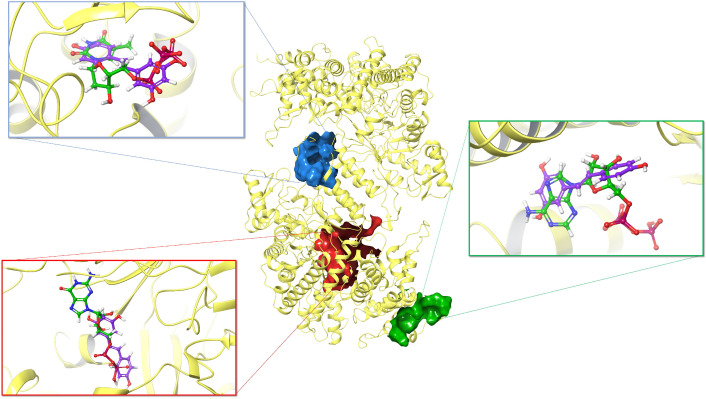
Superposition of the docking poses of *trans*-resveratrol (purple) and substrates (green) on ribonucleotide reductase (PDB ID: 5TUS). The blue surface refers to the S site, and the substrate inside is TTP; the red surface refers to the C site, and the substrate inside is GDP; the green surface refers to the A site, and the substrate inside is ATP.

Ribonucleotide reductase (RNR) is an enzyme that converts ribonucleotides to deoxyribonucleotides which are the substrate of DNA synthesis. Accordingly, RNR plays a critical role in regulating DNA synthesis and repair [44]. Therefore, RNR has been exploited as an important target for cancer drug discovery [45]. Here, we selected RNR (PDB ID: 5TUS) as the third target to test the inhibitory effects of resveratrol and its analogs [46]. There are three binding sites on RNR, namely, the catalytic site (C site), the allosteric site (A site), and the substrate specificity site (S site) [47] (Figure 6). To investigate the inhibitory mechanism of resveratrol, we first docked *trans*-resveratrol onto the three sites of RNR. As control, we docked substrates onto their own binding sites. Table 2 shows that the MM-GBSA energy of *trans*-resveratrol is close to that of the substrate on the A site, however, the *trans*-resveratrol does not show better binding affinities compared with the substrates on S site and C site even though steric clash is revealed after overlapping the docking poses (Figure 6).

Then, we docked the analogs of resveratrol onto those three binding sites separately. For A site, miquelianin shows the best MM-GBSA energy, which is −60.20 kcal/mol. Mulberroside A has the second best binding affinity, giving a score of −58.42 kcal/mol. By comparing the 2-D protein-ligand interaction diagrams (Figures S11–S15), we find that miquelianin and mulberroside A have more interactions with binding site via hydrogen bond than the other compounds, which is consistent with their better binding affinities. For S site, even though M8 shows the best binding affinity (−65.52 kcal/mol) than the other compounds, its MM-GBSA energy is still worse than than of substrate (−76.81 kcal/mol). From the 2-D protein-ligand interaction diagrams (Figures S16–S20), we found the hydroxyl groups of M8 on 3${}^{\prime }$-, 4${}^{\prime }$, and 5${}^{\prime }$-positions interact with Ser269, Asn270, Tyr285, Asp287, and Gln288 by forming six hydrogen bonds, so that the binding affinity of M8 is better than *trans*-resveratrol who has less interactions with binding site. For C site, three compounds show better MM-GBSA energies than the substrate (−49.42 kcal/mol), which are pinostilbene (−50.57 kcal/mol), miquelianin (−59.59 kcal/mol), and astringin (−51.77 kcal/mol). Interestingly, most tested compounds interact with Thr607 or Ala245 via hydrogen bond on C site, which indicates that Thr607 and Ala245 are the critical residues for ligand-protein interaction (Figures S21–S25).

## 4. Discussion

Molecular docking is the most widely used computational method in structure-based drug discovery. Advances in computation software and hardware as well as the increase in the number and the resolution of determined protein structures has improved our understanding of the biophysics and force field of molecular docking. However, molecular docking can be applied beyond drug screening. In this study, we docked resveratrol and its analogs to nucleotide-related enzymes including DNA polymerases, HIV-1 reverse transcriptase, and ribonucleotide reductase. Previous studies have demonstrated the binding and inhibitory effects of resveratrol to these enzymes [13,14,15,48]; however, neither the mechanisms nor the connections of these binding activity have been fully elucidated. Using molecular docking, we reveal that resveratrol and its analogs bind to the 3${}^{\prime }$ end of the elongating DNA or RNA strand and interact with the binding residues of incoming NTP. However, to decrease the computational load, most of the docking algorithms do not fully take the flexibility of the protein structure into account. A determined structure (PDB) of a protein is just a static snapshot of the protein, but the real structure of protein is fluctuating and dynamic. Usually, a ”flexible” docking algorithm considers the side chain fluctuations only or uses multiple structures when take the main chain fluctuation into account. Therefore, the accuracy of the docking results highly relies on the quality of determined protein structure (PDB). Because RNA polymerase is a large complex and its PDB structure has a relatively poor resolution (PDB ID: 5IYD, resolution: 3.9 Å) [49], the docking scores of resveratrol, analogs, and NTP are all relatively low (Table S1), even though the binding poses suggest the same mechanism for resveratrol. Without X-ray crystallography, point mutagenesis, or other bench experiments, molecular docking can be utilized to simulate molecular interactions and efficiently estimate binding affinities. The flexibility of the protein structure continues to limit wider applications and more accurate output of docking. To avoid the inaccurate calculation caused by bad quality structural details or induce-fit conformational changes, molecular dynamics simulation can be used to offset the limitations of molecular docking. The software simulates the flexible motions of protein-ligand structures and the dynamics of their interactions. MD simulation will correct the inaccurate structural details, simulate the conformational changes after ligand binding, and reaffirm the proposed interactions.

The evidence demonstrating that resveratrol inhibits DNA polymerases implies the potential clinical indication of resveratrol and its natural analogs [12]. Therefore, the elucidation of the mechanisms of binding and function is critical to the modification/optimization of the molecular structure and ultimately, medical application of resveratrol and its derivatives. Based on the results of molecular docking, we propose that resveratrol and its analogs inhibit DNA polymerases by binding to the polymerase site, competing with the incoming nucleotide, and blocking the DNA elongation process. These compounds demonstrate their ability to engage in π-π stacking with the terminal nucleotides of the DNA or RNA strand with aromatic rings while they form hydrogen bonds with DNA- or RNA-binding residues and nucleotides (see Figure 2, Figure 3 and Figures S2–S5). Based on this mechanism, we also predict that resveratrol and its analogs must bind to other enzymes involved in DNA and RNA elongation, such as RNA polymerase and reverse transcriptase. Our results validated this hypothesis (see Table 1, Figure 5 and Figures S6–S10). The docking results of RNA polymerase (Table S1 and Figures S26–S28) led us to the same conclusion as the results of DNA polymerases and HIV-1 reverse transcriptase. Resveratrol and its analogs bind to and compete for incoming NTP binding sites. In addition, the binding of resveratrol and its analogs onto the three nucleotide binding sites of the ribonucleotide reductase (Table 2, Figure 6 and Figures S11–S25) provide hints on how resveratrol broadly benefits human health in many aspects. There are thousands of ATP- or even NTP-binding proteins in our proteome. If resveratrol (and its derivatives) competes with NTPs for most of the binding sites, it would widely affect the dynamic behavior of many pathways and the entire protein network. Our future studies will verify this theory. Besides, there has been controversy about the low specificity of resveratrol’s effects [50]. It may be relatively difficult to develop a new drug specifically binding to a target based on a promiscuous compound. However, elucidating the mechanisms will still assist us in understanding how natural products interact with our proteome and how to enhance our health with natural products. Previous studies [51,52,53,54,55,56] and our results have demonstrated that resveratrol does interfere with the activities and alter the behavior of many proteins. It was proposed that the conventional one-drug-one-target paradigm cannot accurately describe the drug actions [57]. In reality, a drug usually binds to and affects more than one target at the same time and a target usually binds to more than one drug. We need to consider drug-drug interactions and their synergy. The drug-target interactions would be multilevel.

Many natural derivatives of resveratrol have already been identified and reported to have numerous benefits in enhancing human health due to their anti-cancer, anti-cardiovascular diseases, anti-diabetic, anti-inflammation, anti-oxidation, and anti-neurodegeneration activities [1,5,31,58]. For instance, 4,4${}^{\prime }$-DHS (*trans*-4,4${}^{\prime }$-dihydroxystilbene) was proven to have antitumor and anti-metastatic effects as it inhibits cell proliferation by arresting the cell cycle at the G1/S phase [59]. Quercetin and myricetin, two well-known cancer therapeutic agents/autophagy mediators, prevent tumor proliferation by inducing cell cycle arrest and inhibit DNA and/or RNA polymerases of viruses or other microorganisms [60,61,62,63,64,65,66]. M8 (3,3${}^{\prime }$,4,4${}^{\prime }$,5,5${}^{\prime }$-hexahydroxystilbene) has been shown to inhibit DNA polymerase and arrest the cell cycle [67]. Other natural derivatives, such as pterostilbene (*trans*-3,5-dimethoxy-4${}^{\prime }$-hydroxystilbene), HPSB (3${}^{\prime }$-hydroxypterostilbene), DRG (*trans*-3,5-dihydroxy-4${}^{\prime }$-methoxystilbene), piceatannol (*trans*-3,4,3${}^{\prime }$,5${}^{\prime }$-tetrahydroxystilbene), miquelianin (quercetin 3-*O*-glucuronide), and astringin (3-β-D-glucoside of piceatannol), have been shown to possess potent anti-proliferative and anti-cancer properties [68,69,70,71,72,73,74]. We only examined a few natural derivatives of resveratrol that are well-studied and found in dietary plants and/or wine; those compounds are structurally similar, bind to the same proteins and the same sites, and have similar functions in health. We speculate there must be many more natural derivatives of resveratrol that have yet to be identified and studied. Previously all of the healthy benefits of grapes, berries, and wine were attributed to resveratrol, although its abundance is relatively low and its half-life is short [75]. This evidence suggests that most of the derivatives in this study contribute to the inhibition of enzymes and the health benefits. Moreover, glycosylation of resveratrol or other polyphenolic compounds could enhance water solubility and drug efficacy [76], which is consistent with the results of this study. Our results show that miquelianin, a quercetin metabolite that is present in wine [77], whose binding affinity is lowest to DNA polymerase α, reverse transcriptase, and the A and C sites of ribonucleotide reductase (Table 1 and Table 2). The glucuronidation of quercetin produces an even more potent compound for nucleotide-related enzymes. The synthesized analog, 3,4,5-THS [78,79], possesses similar activities of binding to ribonucleotide reductase (Table 2). These findings indicate the potential to create artificial analogs of resveratrol that bind to target proteins but last longer than resveratrol as these are not as readily metabolized like a natural compound.

## Figures and Tables

**Table 1:** The MM-GBSA energies of the ligands bound to DNA polymerase α (PDB ID: 4Q5V) and HIV-1 reverse transcriptase (PDB ID: 5TXM).

Compound ${}^{\#}$	MM-GBSA $\Delta G_{\mathrm{Bind}}$ (kcal/mol)	
	4Q5V	5TXM
Substrate	−49.46(dCTP)	−47.88(ddATP) −46.41(dATP)
*Trans*-resveratrol	−49.58	−55.42
*Cis*-resveratrol	−48.85	−42.47
Piceatannol	−50.67	−41.76
M8	−59.6	−40.04
3,4,5-THS	−63.64	−38.37
4,4${}^{\prime }$-DHS	−56.1	−41.36
Pterostilbene	−46.68	−40.81
HPSB	−50.51	−42.28
DRG	−45.91	−44.08
Pinostilbene	−43.86	−35.87
Compound-1 ${}^{1}$	−42.87	−27.84
Compound-2 ${}^{2}$	−34.08	−47.19
Compound-3 ${}^{3}$	−52.48	−41.53
Quercetin	−59.08	−52.96
Myricetin	−61.90	−49.87
Miquelianin	−73.53 *	−75.71 *
Astringin	−56.22	−60.16
Mulberroside A	−59.33	−47.25
Hydroxyurea	−20.25	−10.82

${}^{\#}$ The structure of the 19 compounds are shown in Figure S1. * The best binder among the 19 compounds. ${}^{1}$ Compound-1: (E)-5-(4-Nitrostyryl)benzo[d][1,3]dioxole. ${}^{2}$ Compound-2: (E)-5-(4-Nitrostyryl)-1,3-phenylene diacetate. ${}^{3}$ Compound-3: (E)-4-(2,3,4-Trimethoxystyryl)benzenamine.

**Table 2:** The MM-GBSA energies of the ligands bound to three binding sites of ribonucleotide reductase (PDB ID: 5TUS).

Compound ${}^{\#}$	MM-GBSA $\Delta G_{\mathrm{Bind}}$ (kcal/mol)		
	A Site	S Site	C Site
Substrate	−42.27(ATP)	−76.81(TTP)	−49.42(GDP)
*Trans*-resveratrol	−42.14	−52.8	−37.02
*Cis*-resveratrol	−38.22	−51.05	−43.24
Piceatannol	−44.24	−58.18	−45.25
M8	−41.23	−65.52${}^{*}$	−46.42
3,4,5-THS	−39.08	−59.32	−31.65
4,4${}^{\prime }$-DHS	−37.21	−46.51	−35.58
Pterostilbene	−46.8	−51.16	−42.23
HPSB	−34.11	−56.22	−38.31
DRG	−43.66	−58.32	−40.16
Pinostilbene	−32	−55	−50.57
Compound-1 ${}^{1}$	−23.42	−46.50	−38.51
Compound-2 ${}^{2}$	−24.98	−49.11	−40.11
Compound-3 ${}^{3}$	−24.98	−59.82	−29.60
Quercetin	−24.14	−59.47	−46.89
Myricetin	−30.91	−59.43	−46.59
Miquelianin	−60.20 *	−62.45	−59.59 *
Astringin	−50.80	−58.74	−51.77
Mulberroside A	−58.42	−65.12	−48.44
Hydroxyurea	−14.73	−24.24	−20.80

${}^{\#}$ The structure of the 19 compounds are shown in Figure S1. * The most effective binder to DNA polymerase α among 19 compounds tested. ${}^{1}$ Compound-1: (E)-5-(4-Nitrostyryl)benzo[d][1,3]dioxole. ${}^{2}$ Compound-2: (E)-5-(4-Nitrostyryl)-1,3-phenylene diacetate. ${}^{3}$ Compound-3: (E)-4-(2,3,4-Trimethoxystyryl)benzenamine.

## Supplementary Materials

The following are available online at https://www.mdpi.com/2218-273X/10/9/1223/s1, Figure S1: Resveratrol and its analogs; Figure S2: 2-D protein-ligand interaction diagrams of 4Q5V and five ligands: Cis-resveratrol (A), Piceatannol (B), M8 (C), 3,4,5-THS (D), and 4,4′-DHS (E); Figure S3: 2-D protein-ligand interaction diagrams of 4Q5V and four ligands: Pterostilbene (F), HPSB (G), DRG (H), and Pinostilbene (I); Figure S4: 2-D protein-ligand interaction diagrams of 4Q5V and four ligands: Compound-1 (J), Compound-2 (K), Compound-3 (L), and Quercetin (M); Figure S5: 2-D protein-ligand interaction diagrams of 4Q5V and three ligands: Astringin (N), Mulberroside A (O), and Hydroxyurea (P); Figure S6: 2-D protein-ligand interaction diagrams of 5TXM and four ligands: dATP (A), ddATP (B), Trans-resveratrol (C), and Cis-resveratrol (D); Figure S7: 2-D protein-ligand interaction diagrams of 5TXM and four ligands: Piceatannol (E), M8 (F), 3,4,5-THS (G), and 4,4′-DHS (H); Figure S8: 2-D protein-ligand interaction diagrams of 5TXM and four ligands: Pterostilbene (I), HPSB (J), DRG (K), and Pinostilbene (L); Figure S9: 2-D protein-ligand interaction diagrams of 5TXM and five ligands: Compound-1 (M), Compound-2 (N), Compound-3 (O), Quercetin (P), and Myricetin (Q); Figure S10: 2-D protein-ligand interaction diagrams of 5TXM and four ligands: Miquelianin (R), Astringin (S), Mulberroside A (T), and Hydroxyurea (U); Figure S11: 2-D protein-ligand interaction diagrams of A site on 5TUS and three ligands: ATP (A), Trans-resveratrol (B), and Cis-resveratrol (C); Figure S12: 2-D protein-ligand interaction diagrams of A site on 5TUS and four ligands: Piceatannol (D), M8 (E), 3,4,5-THS (F) and 4,4′-DHS (G); Figure S13: 2-D protein-ligand interaction diagrams of A site on 5TUS and four ligands: Pterostilbene (H), HPSB (I), DRG (J), and Pinostilbene (K); Figure S14: 2-D protein-ligand interaction diagrams of A site on 5TUS and five ligands: Compound-1 (L), Compound-2 (M), Compound-3 (N), Quercetin (O), and Myricetin (P); Figure S15: 2-D protein-ligand interaction diagrams of A site on 5TUS and four ligands: Miquelianin (Q), Astringin (R), Mulberroside A (S), and Hydroxyurea (T); Figure S16: 2-D protein-ligand interaction diagrams of S site on 5TUS and three ligands: TTP (A), Trans-resveratrol (B), and Cis-resveratrol (C); Figure S17: 2-D protein-ligand interaction diagrams of S site on 5TUS and four ligands: Piceatannol (D), M8 (E), 3,4,5-THS (F) and 4,4′-DHS (G); Figure S18: 2-D protein-ligand interaction diagrams of S site on 5TUS and four ligands: Pterostilbene (H), HPSB (I), DRG (J), and Pinostilbene (K); Figure S19: 2-D protein-ligand interaction diagrams of S site on 5TUS and five ligands: Compound-1 (L), Compound-2 (M), Compound-3 (N), Quercetin (O), and Myricetin (P); Figure S20: 2-D protein-ligand interaction diagrams of S site on 5TUS and four ligands: Miquelianin (Q), Astringin (R), Mulberroside A (S), and Hydroxyurea (T); Figure S21: 2-D protein-ligand interaction diagrams of C site on 5TUS and three ligands: GDP (A), Trans-resveratrol (B), and Cis-resveratrol (C); Figure S22: 2-D protein-ligand interaction diagrams of C site on 5TUS and four ligands: Piceatannol (D), M8 (E), 3,4,5-THS (F) and 4,4′-DHS (G); Figure S23: 2-D protein-ligand interaction diagrams of C site on 5TUS and four ligands: Pterostilbene (H), HPSB (I), DRG (J), and Pinostilbene (K); Figure S24: 2-D protein-ligand interaction diagrams of C site on 5TUS and five ligands: Compound-1 (L), Compound-2 (M), Compound-3 (N), Quercetin (O), and Myricetin (P); Figure S25: 2-D protein-ligand interaction diagrams of C site on 5TUS and four ligands: Miquelianin (Q), Astringin (R), Mulberroside A (S), and Hydroxyurea (T); Figure S26: 2-D protein-ligand interaction diagrams of 5IYD and four ligands: GTP (A), Resveratrol (B), Miquelianin (C), and Piceatannol (D); Figure S27: 2-D protein-ligand interaction diagrams of 5IYD and four ligands: Pterostilbene (E), Quercetin (F), HPSB (G), and DRG (H); Figure S28: 2-D protein-ligand interaction diagrams of 5IYD and four ligands: Astringin (I), M8 (J), 3,4,5-THS (K), and 4,4′-DHS (L); Figure S29: Superposition of the docked aphidicolin (yellow) and the determined structure of aphidicolin (green) on DNA polymerase *α* (PDB ID: 4Q5V). The MM-GBSA energy is -62.22 kcal/mol; Table S1: The MM-GBSA energies of the ligands bound to RNA polymerase (5IYD) and DNA polymerase *δ* (predicted model); Figure S30: Docking poses of 4Q5V and six ligands: Cis-resveratrol (A), Piceatannol (B), M8 (C), 3,4,5-THS (D), 4,4′-DHS (E), and Pterostilbene (F); Figure S31: Docking poses of 4Q5V and six ligands: HPSB (G), DRG (H), Pinostilbene (I), Compound-1 (J), Compound-2 (K), and Compound-3 (L); Figure S32: Docking poses of 4Q5V and six ligands: Quercetin (M), Myricetin (N), Astringin (O), Miquelianin (P), Mulberroside A (Q), and Hydroxyurea (R); Figure S33: Docking poses of 5TXM and six ligands: Cis-resveratrol (A), Piceatannol (B), M8 (C), 3,4,5-THS (D), 4,4′-DHS (E), and Pterostilbene (F); Figure S34: Docking poses of 5TXM and six ligands: HPSB (G), DRG (H), Pinostilbene (I), Compound-1 (J), Compound-2 (K), and Compound-3 (L); Figure S35: Docking poses of 5TXM and six ligands: Quercetin (M), Myricetin (N), Astringin (O), Miquelianin (P), Mulberroside A (Q), and Hydroxyurea (R); Figure S36: Docking poses of A site on 5TUS and six ligands: Cis-resveratrol (A), Piceatannol (B), M8 (C), 3,4,5-THS (D), 4,4′-DHS (E), and Pterostilbene (F); Figure S37: Docking poses of A site on 5TUS and six ligands: HPSB (G), DRG (H), Pinostilbene (I), Compound-1 (J), Compound-2 (K), and Compound-3 (L); Figure S38: Docking poses of A site on 5TUS and six ligands: Quercetin (M), Myricetin (N), Astringin (O), Miquelianin (P), Mulberroside A (Q), and Hydroxyurea (R); Figure S39: Docking poses of S site on 5TUS and six ligands: Cis-resveratrol (A), Piceatannol (B), M8 (C), 3,4,5-THS (D), 4,4′-DHS (E), and Pterostilbene (F); Figure S40: Docking poses of S site on 5TUS and six ligands: HPSB (G), DRG (H), Pinostilbene (I), Compound-1 (J), Compound-2 (K), and Compound-3 (L); Figure S41: Docking poses of S site on 5TUS and six ligands: Quercetin (M), Myricetin (N), Astringin (O), Miquelianin (P), Mulberroside A (Q), and Hydroxyurea (R); Figure S42: Docking poses of C site on 5TUS and six ligands: Cis-resveratrol (A), Piceatannol (B), M8 (C), 3,4,5-THS (D), 4,4′-DHS (E), and Pterostilbene (F); Figure S43: Docking poses of C site on 5TUS and six ligands: HPSB (G), DRG (H), Pinostilbene (I), Compound-1 (J), Compound-2 (K), and Compound-3 (L); Figure S44: Docking poses of C site on 5TUS and six ligands: Quercetin (M), Myricetin (N), Astringin (O), Miquelianin (P), Mulberroside A (Q), and Hydroxyurea (R).

## Abbreviations

The following abbreviations are used in this manuscript:

MD simulation	molecular dynamics simulation
PDB	Protein Data Bank
RMSD	rootmean-square deviation
M8	3,3${}^{\prime }$,4,4${}^{\prime }$,5,5${}^{\prime }$-hexahydroxystilbene
DRG	trans-3,5-dihydroxy-4${}^{\prime }$-methoxystilbene
4,4${}^{\prime }$-DHS	trans-4,4${}^{\prime }$-dihydroxystilbene
HPSB	3${}^{\prime }$-hydroxypterostilbene
3,4,5-THS	trans-3${}^{\prime }$,4${}^{\prime }$,5${}^{\prime }$-trihydroxystilbene
dCTP	deoxycytidine triphosphate
dATP	deoxyadenosine triphosphate
ddATP	2’,3${}^{\prime }$-dideoxyadenosine triphosphate
RNR	ribonucleotide reductase
Ki	inhibition constant for the inhibitor
