# Supplementary material for: Elucidating the Inhibitory Effect of Resveratrol and Its Structural Analogs on Selected Nucleotide-Related Enzymes
Source: Biomolecules. 2020 Aug 22;10(9):1223. doi: 10.3390/biom10091223 (PMC7563984; doi:10.3390/biom10091223)
Supplement: Supplementary file 1 [file biomolecules-10-01223-s001.pdf]

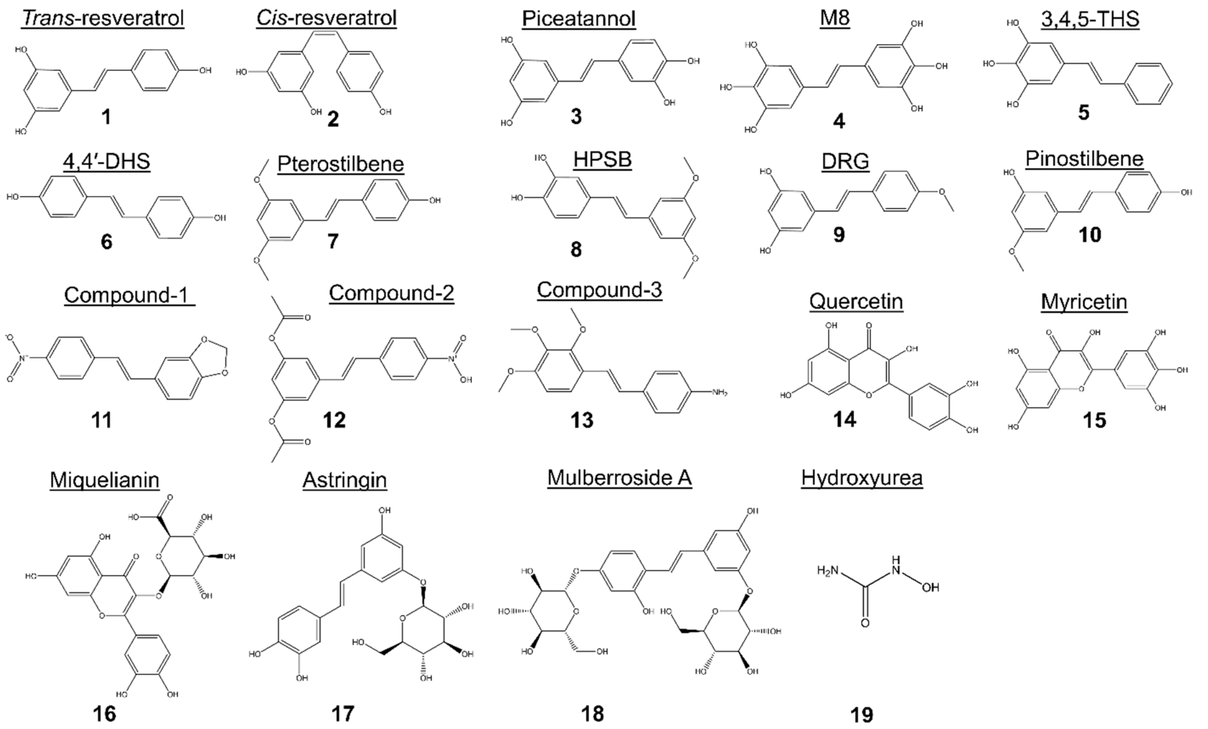

Figure S1 Resveratrol and its analogs (1-18). 1. *Trans*-resveratrol; **2**. *Cis*-resveratrol; **3.** Piceatannol; **4**. M8; **5**. 3,4,5-THS; **6**. 4,4′-DHS; **7**. Pterostilbene; **8**. HPSB; **9**. DRG; **10**. Pinostilbene; **11**.Compound-1; **12**. Compound-2; **13**. Compound-3; **14**. Quercetin; **15**. Myricetin; **16**. Miquelianin; **17**. Astringin; **18**. Mulberroside A; **19**. Hydroxyurea (negative control).

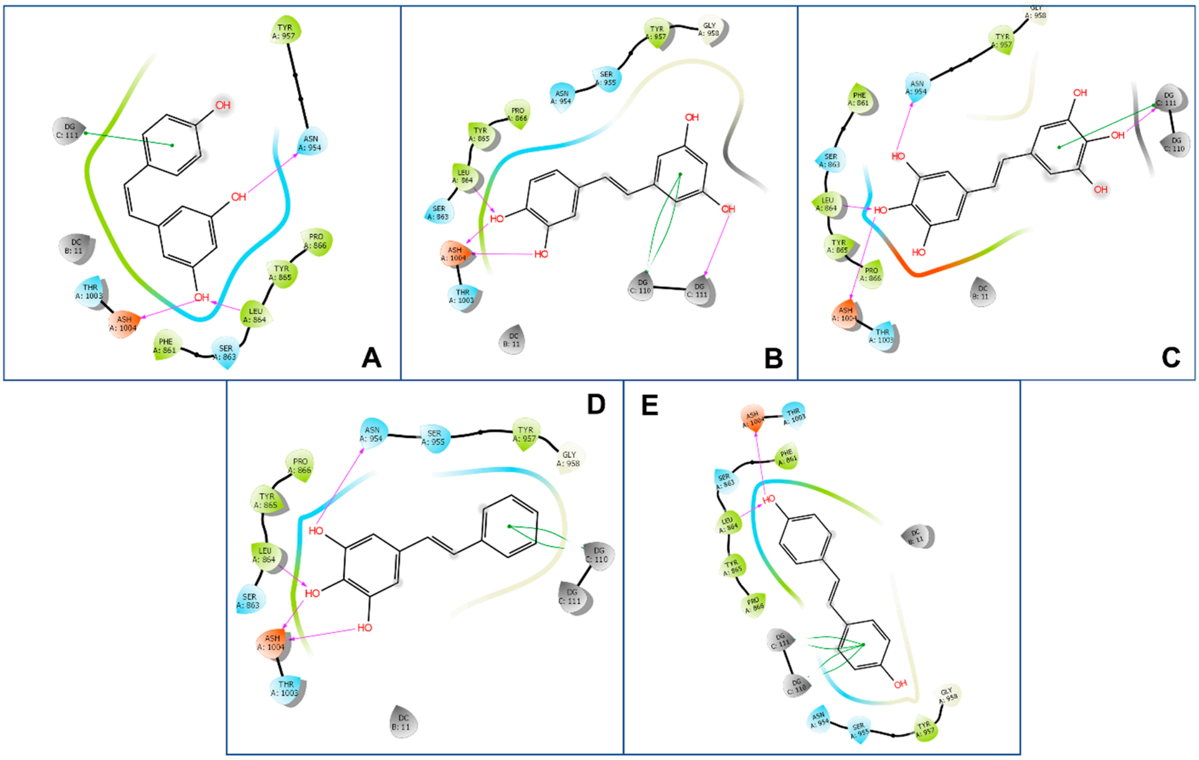

Figure S2 2-D protein-ligand interaction diagrams of 4Q5V and five ligands: *Cis*-resveratrol (A), Piceatannol (B), M8 (C), 3,4,5-THS (D), and 4,4′-DHS (E). The purple arrow indicates the hydrogen bond; the green line indicates the π-π stacking.

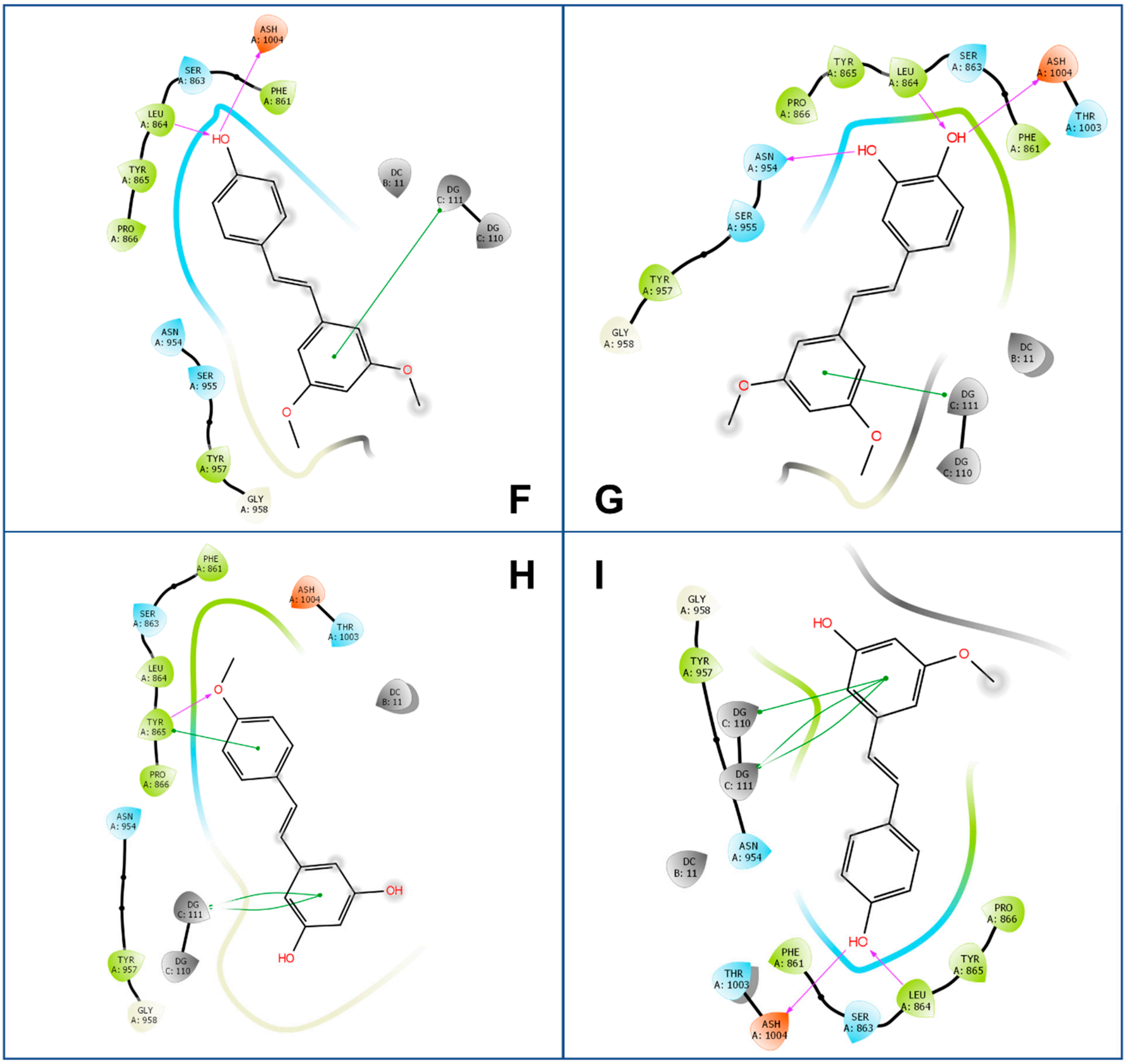

Figure S3 2-D protein-ligand interaction diagrams of 4Q5V and four ligands: Pterostilbene (F), HPSB (G), DRG (H), and Pinostilbene (I). The purple arrow indicates the hydrogen bond; the green line indicates the π-π stacking.

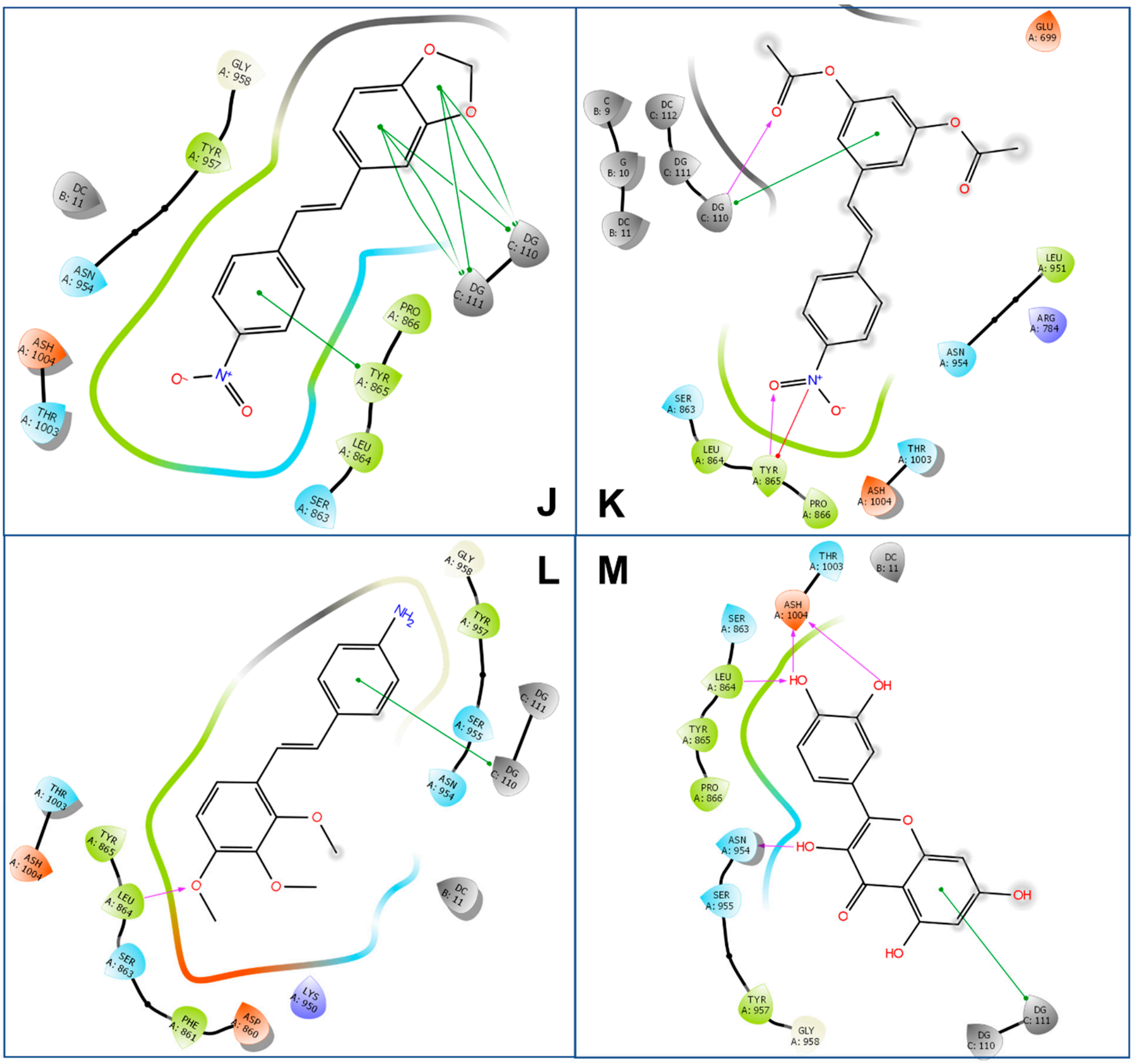

Figure S4 2-D protein-ligand interaction diagrams of 4Q5V and four ligands: Compound-1 (J), Compound-2 (K), Compound-3 (L), and Quercetin (M). The purple arrow indicates the hydrogen bond; the green line indicates the π-π stacking; the red line indicates the π-cation.

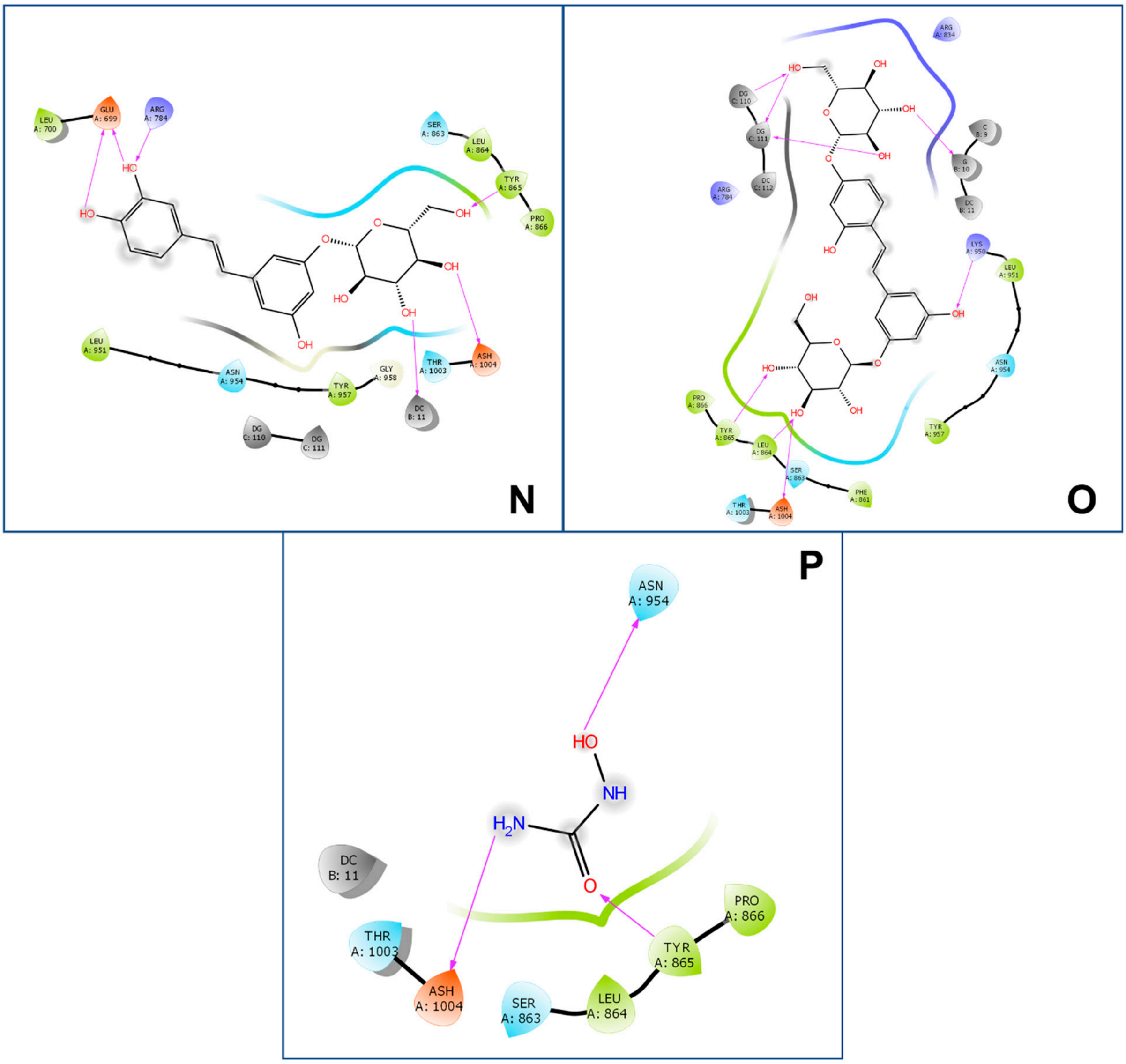

Figure S5 2-D protein-ligand interaction diagrams of 4Q5V and three ligands: Astringin (N), Mulberroside A (O), and Hydroxyurea (P). The purple arrow indicates the hydrogen bond.

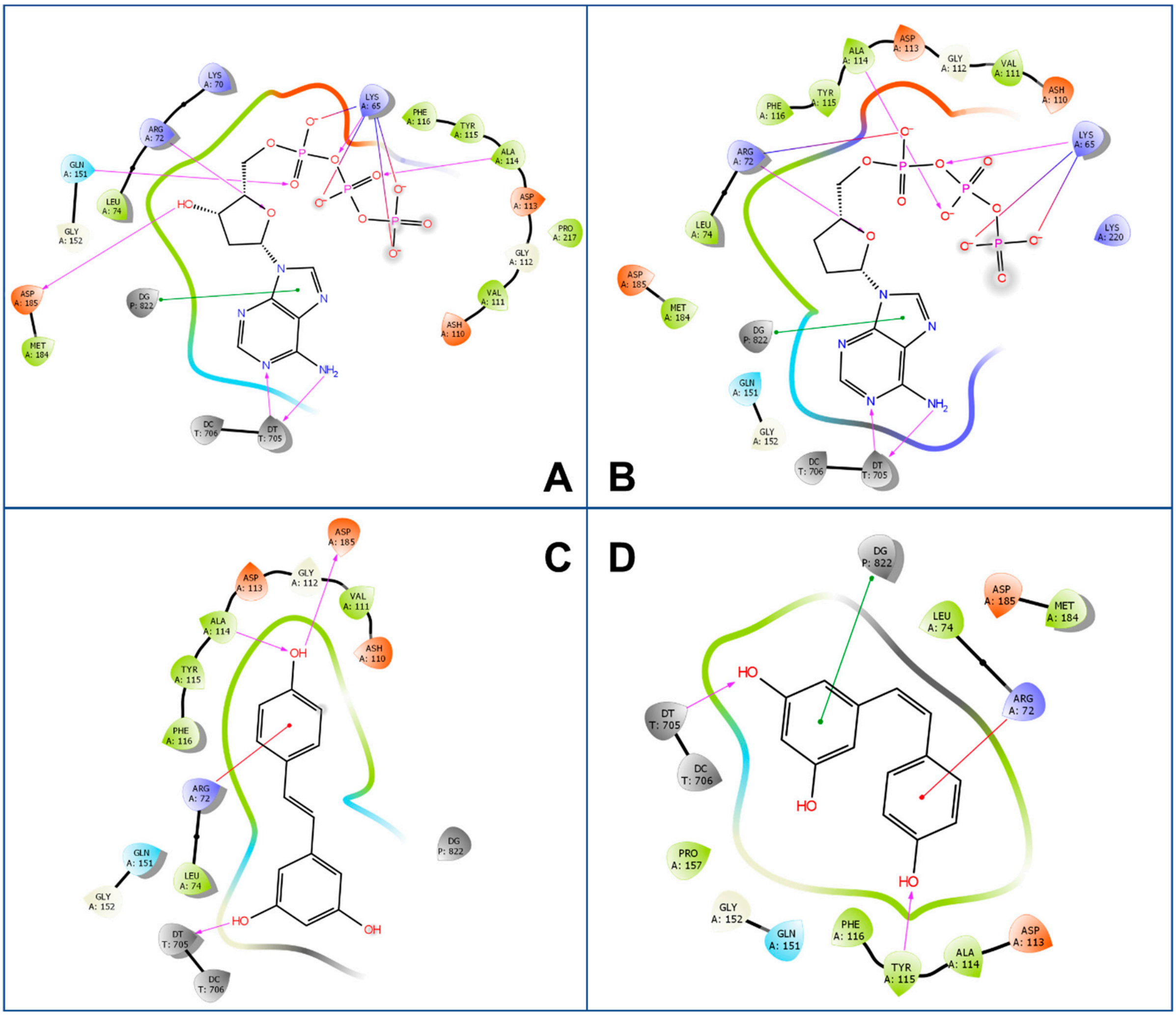

Figure S6 2-D protein-ligand interaction diagrams of 5TXM and four ligands: dATP (A), ddATP (B), *Trans*-resveratrol (C), and *Cis*-resveratrol (D). The purple arrow indicates the hydrogen bond; the green line indicates the π-π stacking; the red line indicates the π-cation; the blue-red line indicates the salt bridge.

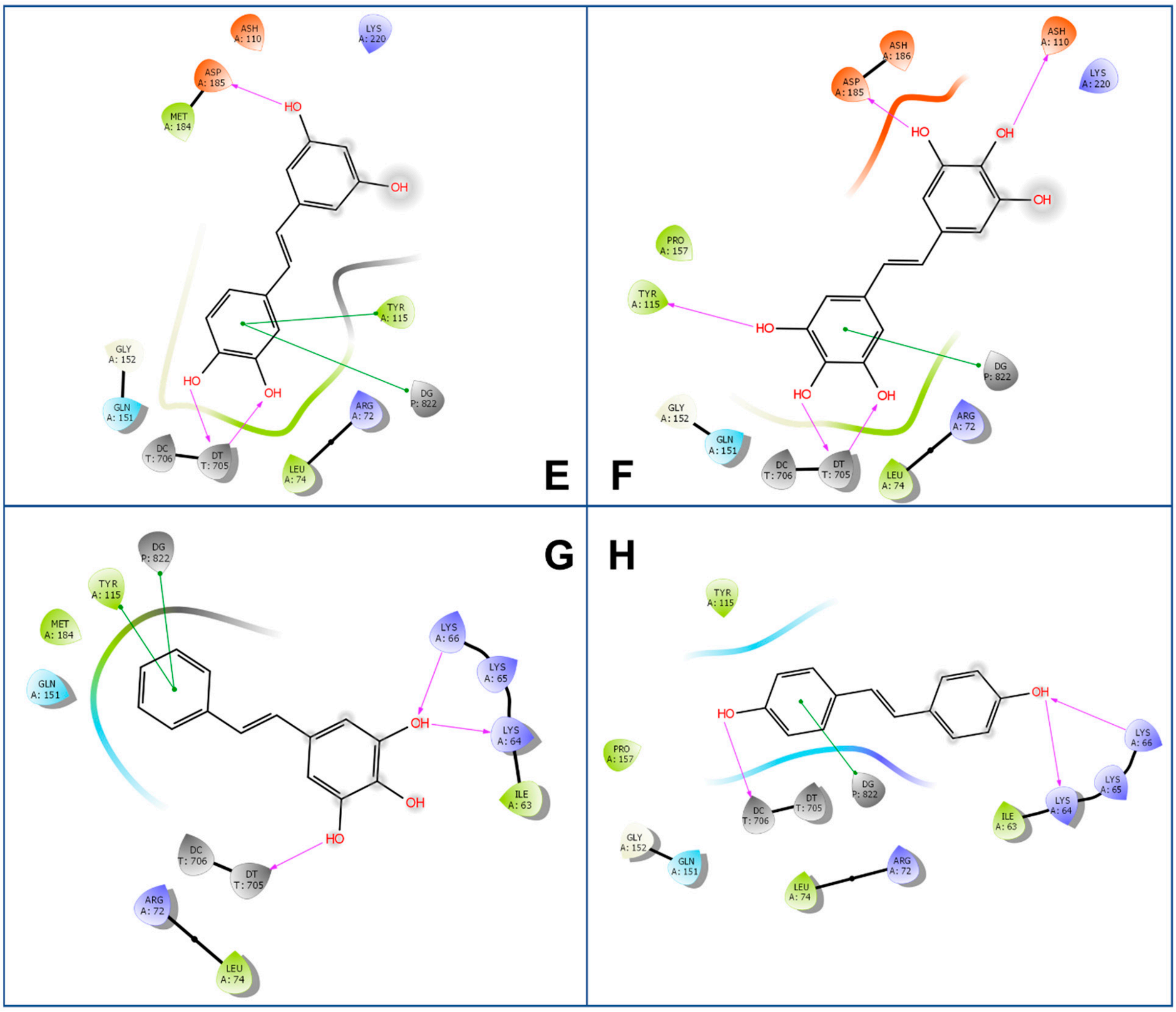

Figure S7 2-D protein-ligand interaction diagrams of 5TXM and four ligands: Piceatannol (E), M8 (F), 3,4,5-THS (G), and 4,4′-DHS (H). The purple arrow indicates the hydrogen bond; the green line indicates the π-π stacking.

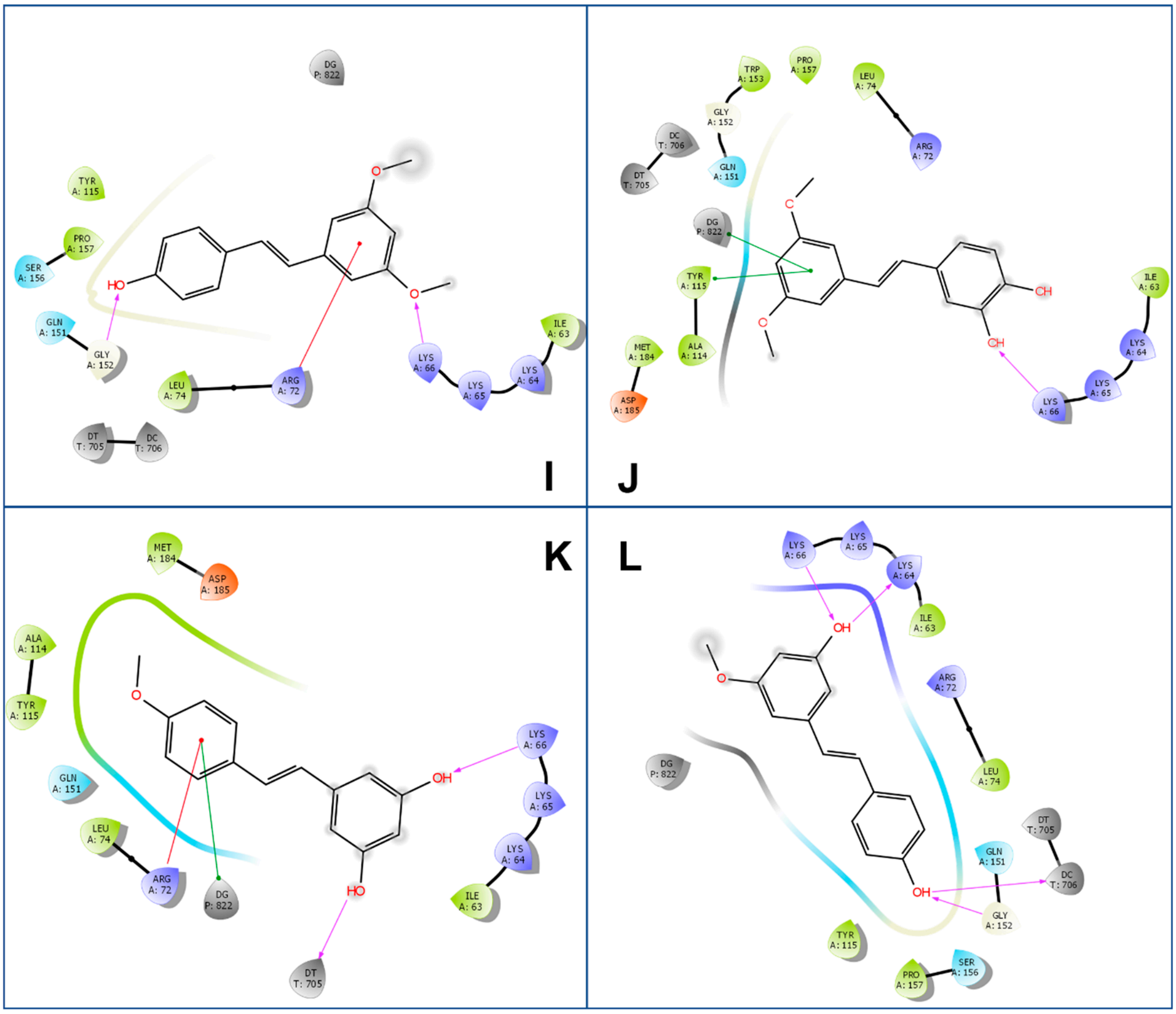

Figure S8 2-D protein-ligand interaction diagrams of 5TXM and four ligands: Pterostilbene (I), HPSB (J), DRG (K), and Pinostilbene (L). The purple arrow indicates the hydrogen bond; the green line indicates the π-π stacking; the red line indicates the π-cation.

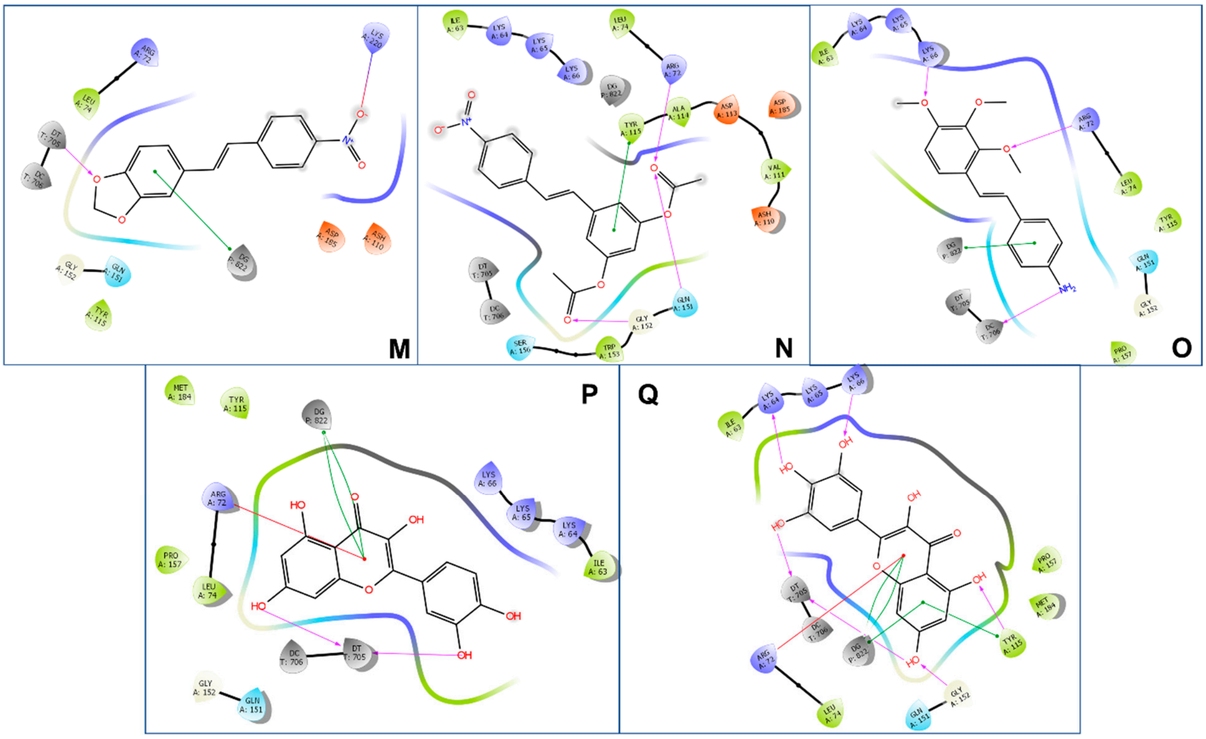

Figure S9 2-D protein-ligand interaction diagrams of 5TXM and five ligands: Compound-1 (M), Compound-2 (N), Compound-3 (O), Quercetin (P), and Myricetin (Q). The purple arrow indicates the hydrogen bond; the green line indicates the π-π stacking, the red line indicates the π-cation.

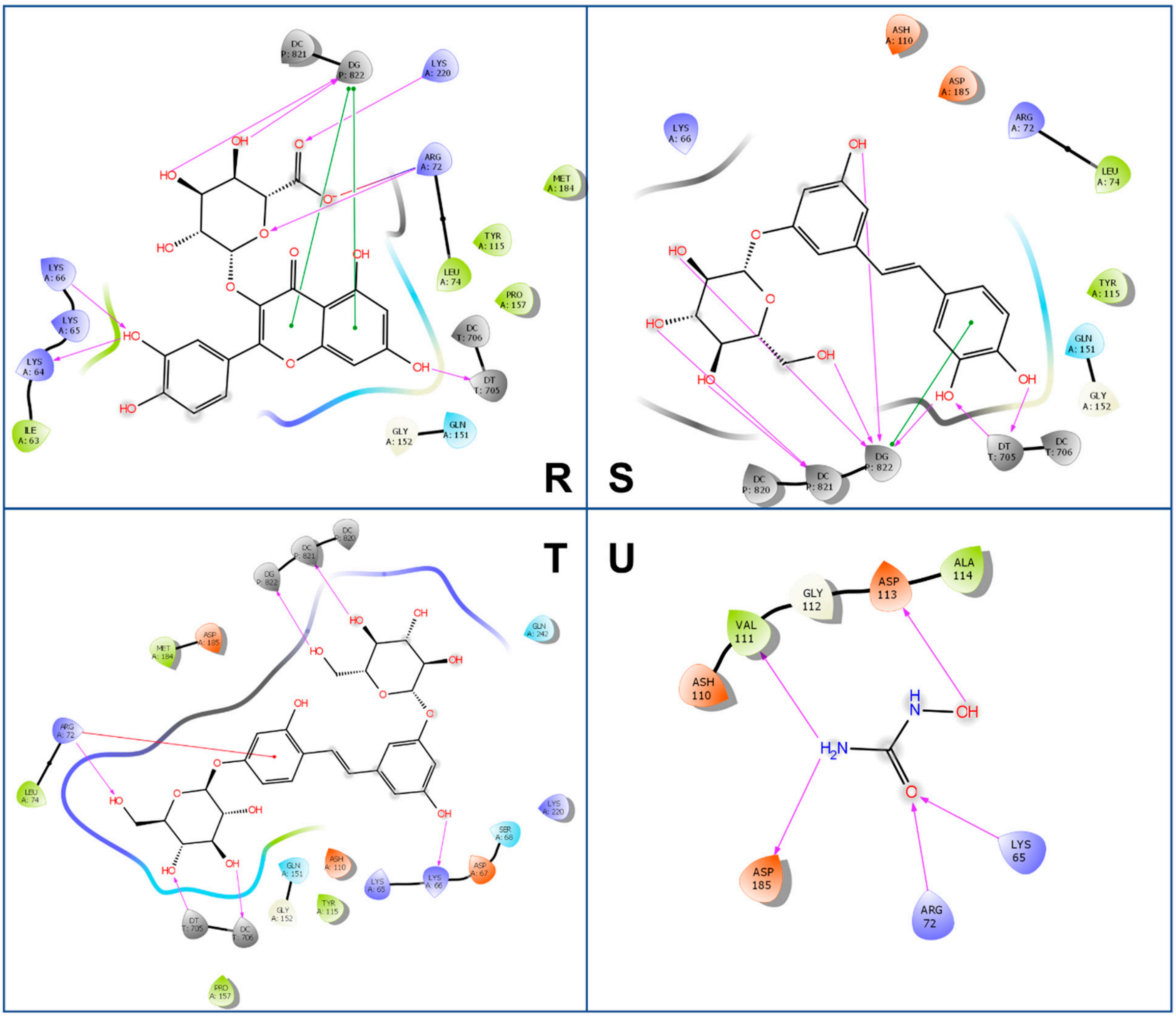

Figure S10 2-D protein-ligand interaction diagrams of 5TXM and four ligands: Miquelianin (R), Astringin (S), Mulberroside A (T), and Hydroxyurea (U). The purple arrow indicates the hydrogen bond; the green line indicates the π-π stacking, the red line indicates the π-cation.

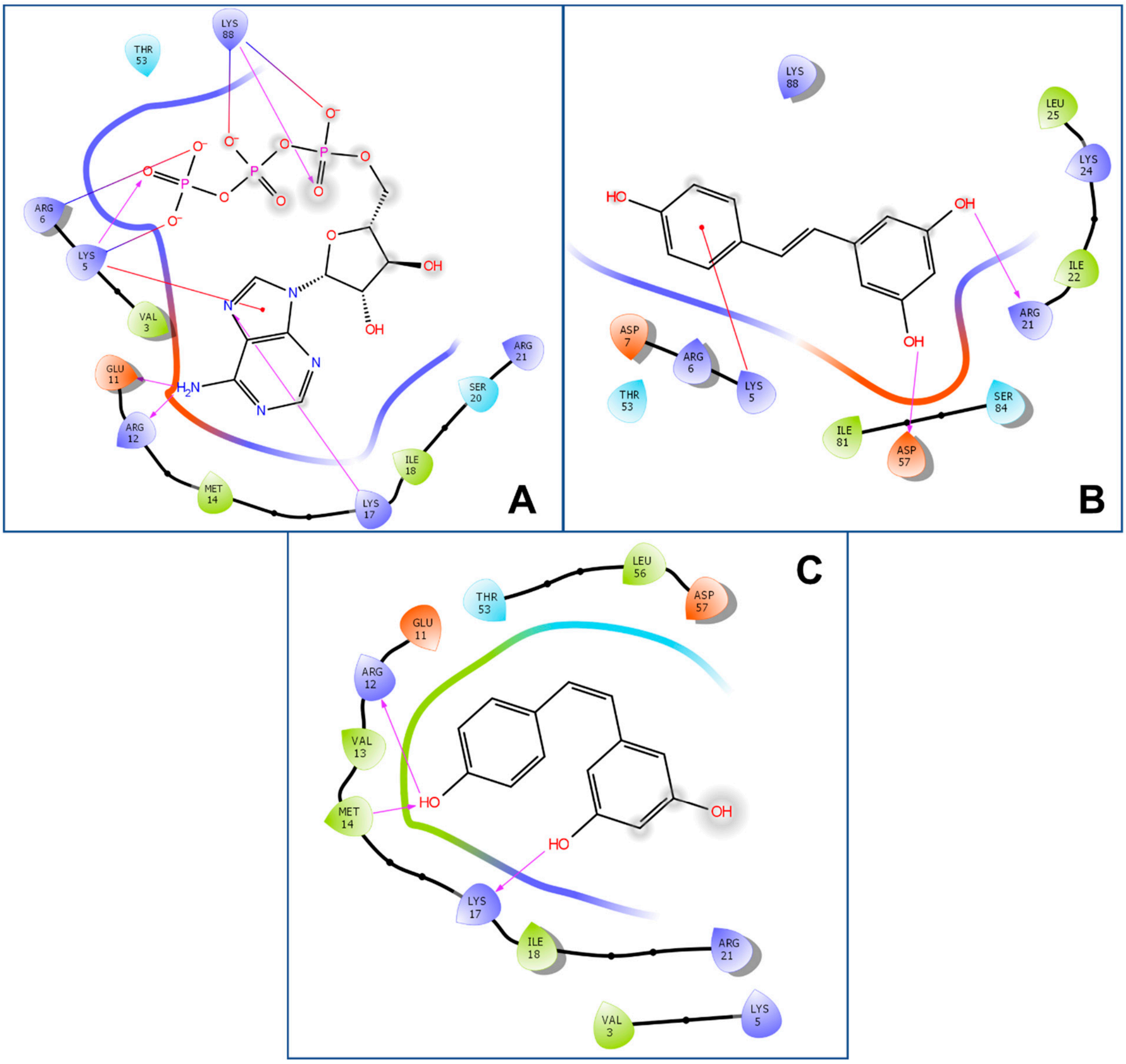

Figure S11 2-D protein-ligand interaction diagrams of A site on 5TUS and three ligands: ATP (A), *Trans*-resveratrol (B), and *Cis*-resveratrol (C). The purple arrow indicates the hydrogen bond; the red line indicates the π-cation; the blue-red line indicates the salt bridge.

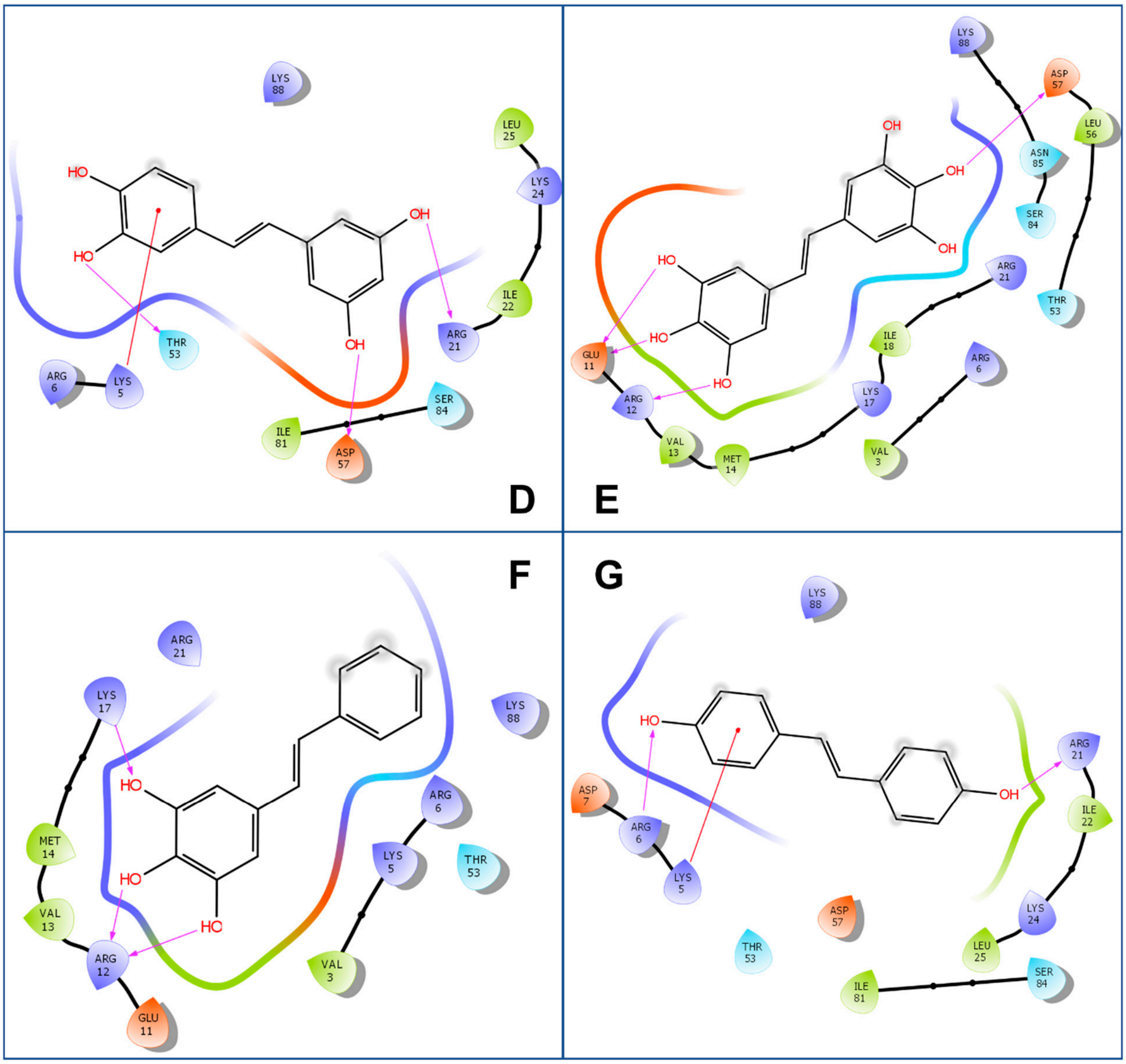

Figure S12 2-D protein-ligand interaction diagrams of A site on 5TUS and four ligands: Piceatannol (D), M8 (E), 3,4,5-THS (F) and 4,4′-DHS (G). The purple arrow indicates the hydrogen bond; the red line indicates the π-cation.

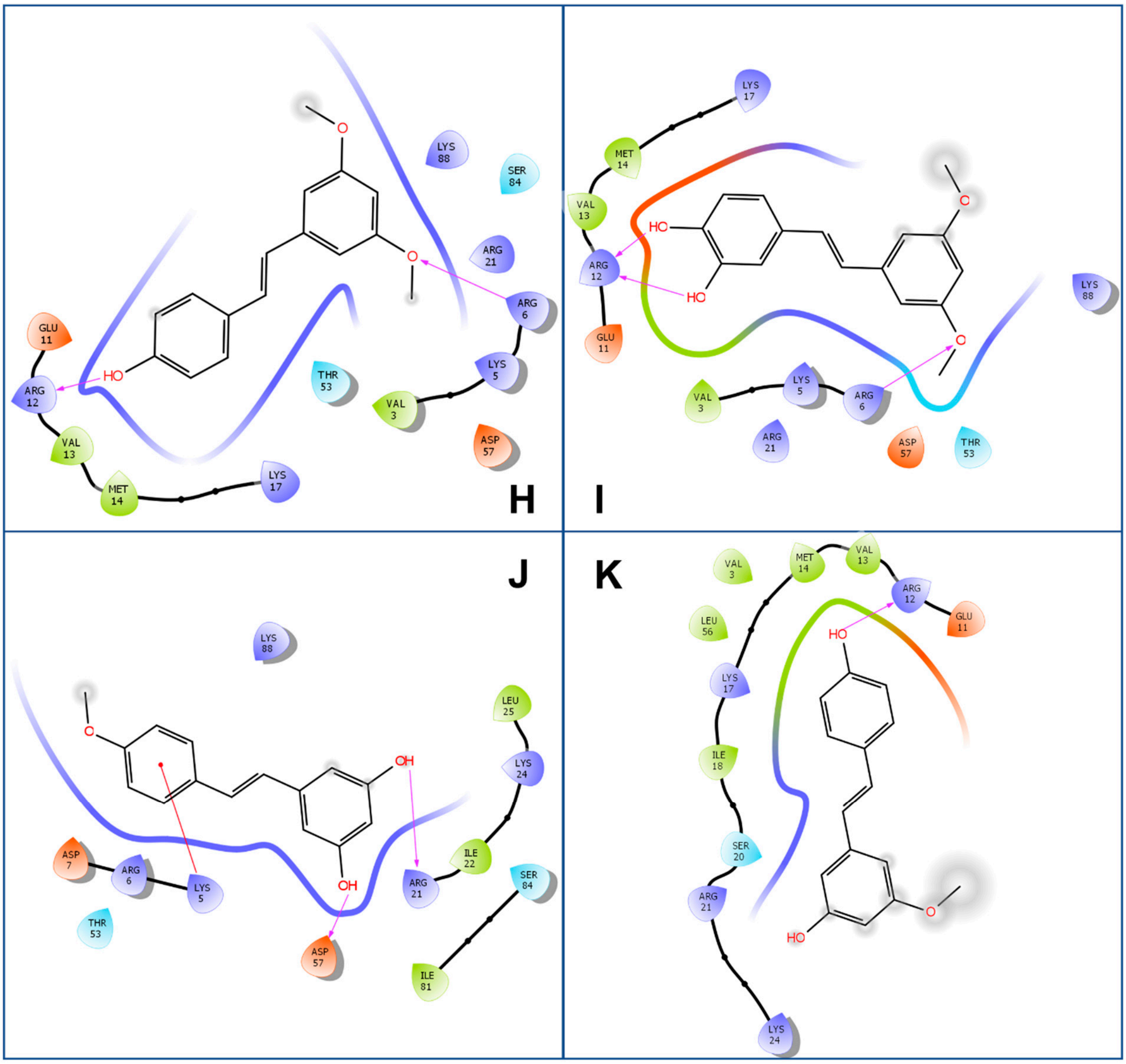

Figure S13 2-D protein-ligand interaction diagrams of A site on 5TUS and four ligands: Pterostilbene (H), HPSB (I), DRG (J), and Pinostilbene (K). The purple arrow indicates the hydrogen bond; the red line indicates the π-cation.

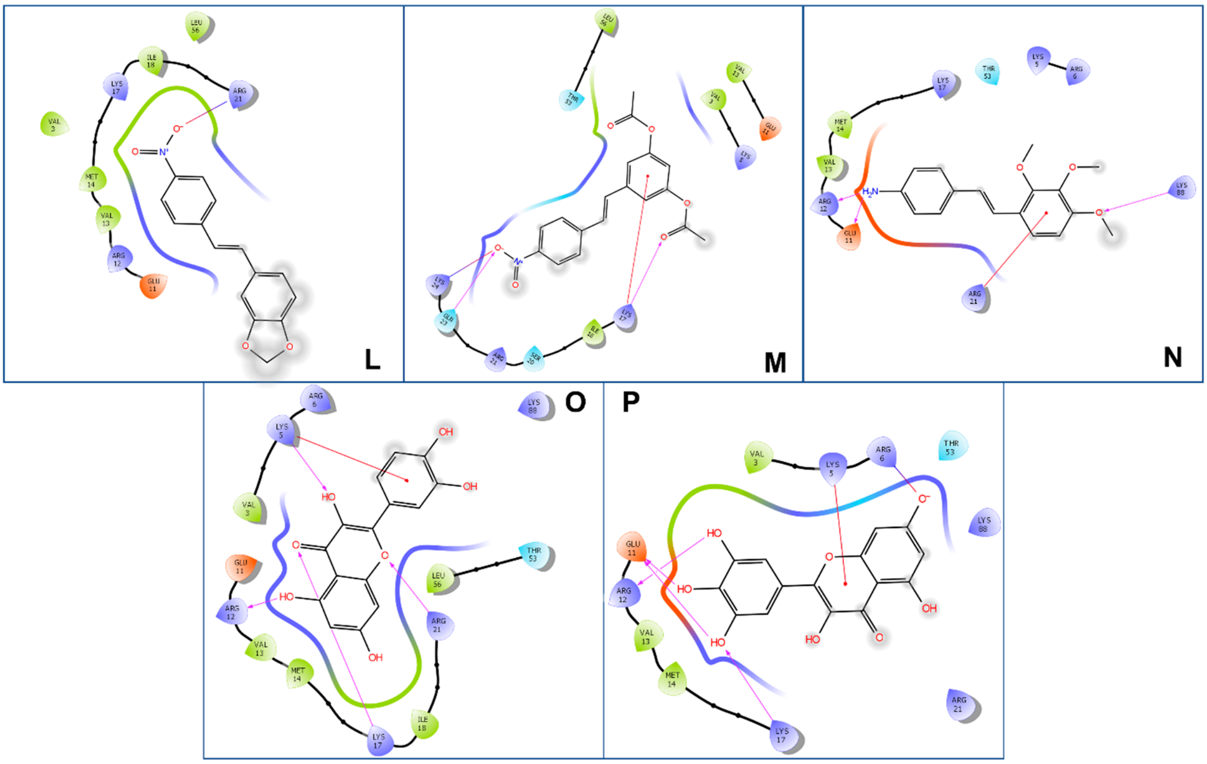

Figure S14 2-D protein-ligand interaction diagrams of A site on 5TUS and five ligands: Compound-1 (L), Compound-2 (M), Compound-3 (N), Quercetin (O), and Myricetin (P). The purple arrow indicates the hydrogen bond; the red line indicates the π-cation, the blue-red line indicates the salt bridge.

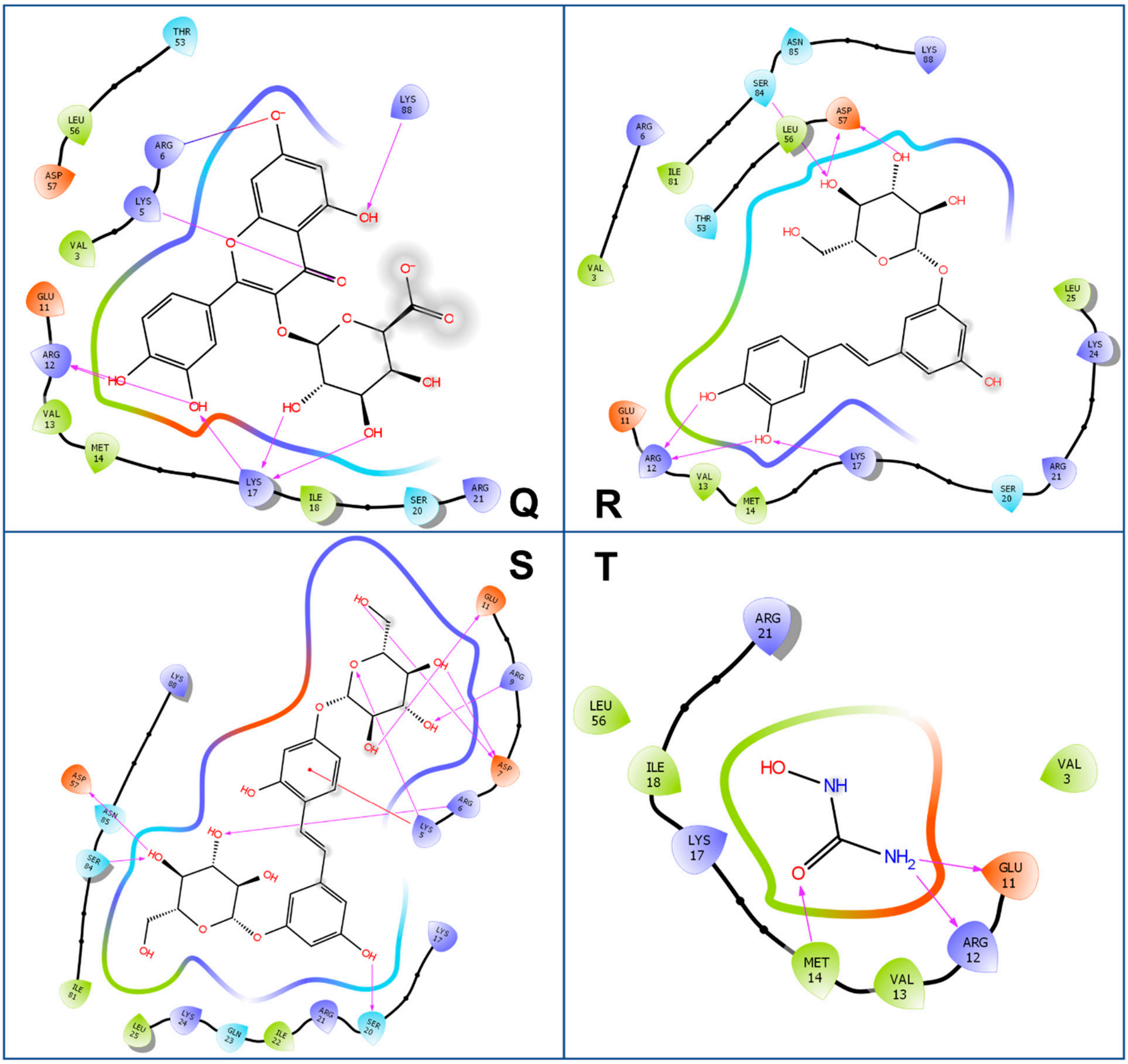

Figure S15 2-D protein-ligand interaction diagrams of A site on 5TUS and four ligands: Miquelianin (Q), Astringin (R), Mulberroside A (S), and Hydroxyurea (T). The purple arrow indicates the hydrogen bond; the blue-red line indicates the salt bridge.

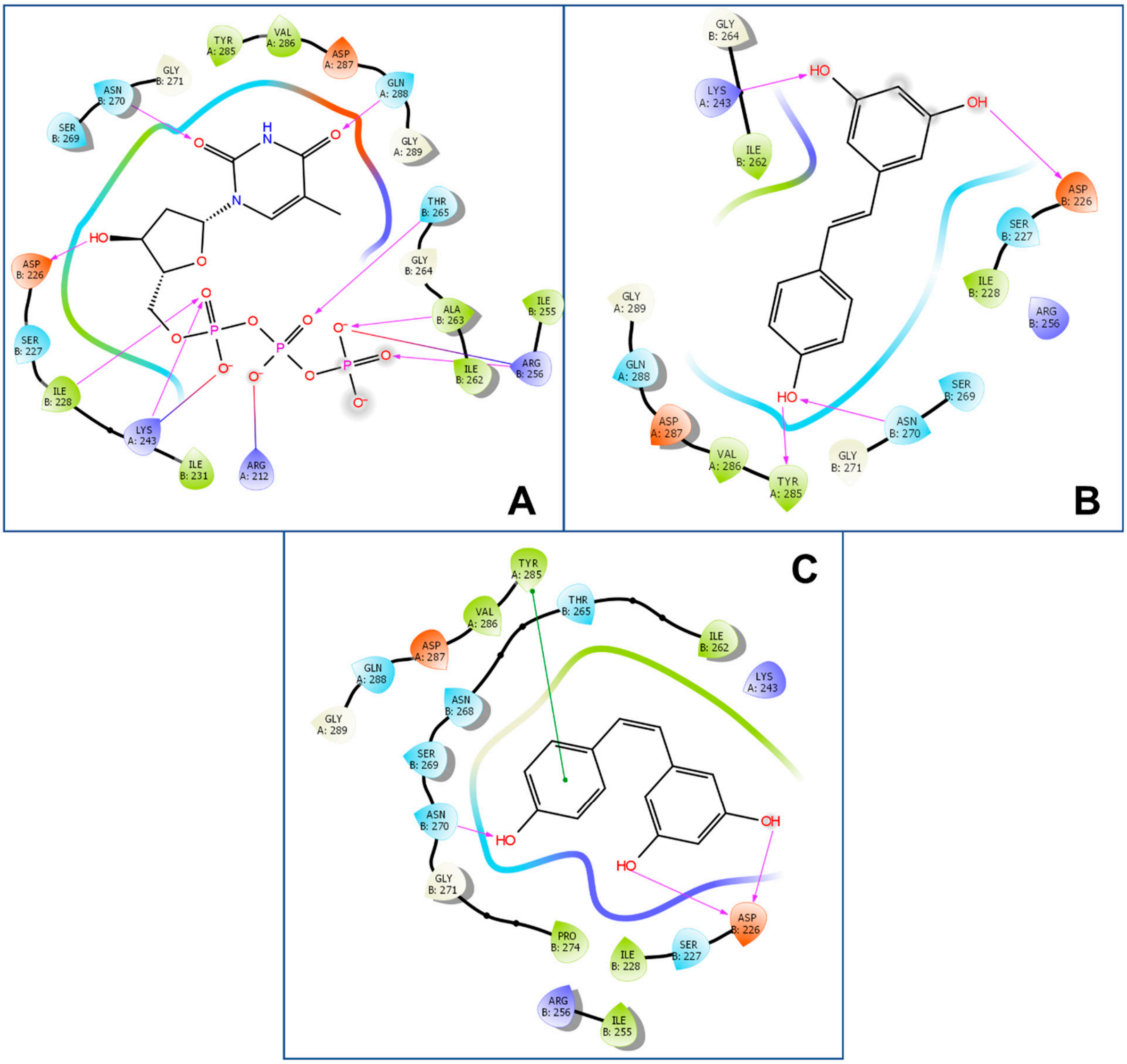

Figure S16 2-D protein-ligand interaction diagrams of S site on 5TUS and three ligands: TTP (A), *Trans*-resveratrol (B), and *Cis*-resveratrol (C). The purple arrow indicates the hydrogen bond; the green line indicates the π-π stacking; the blue-red line indicates the salt bridge.

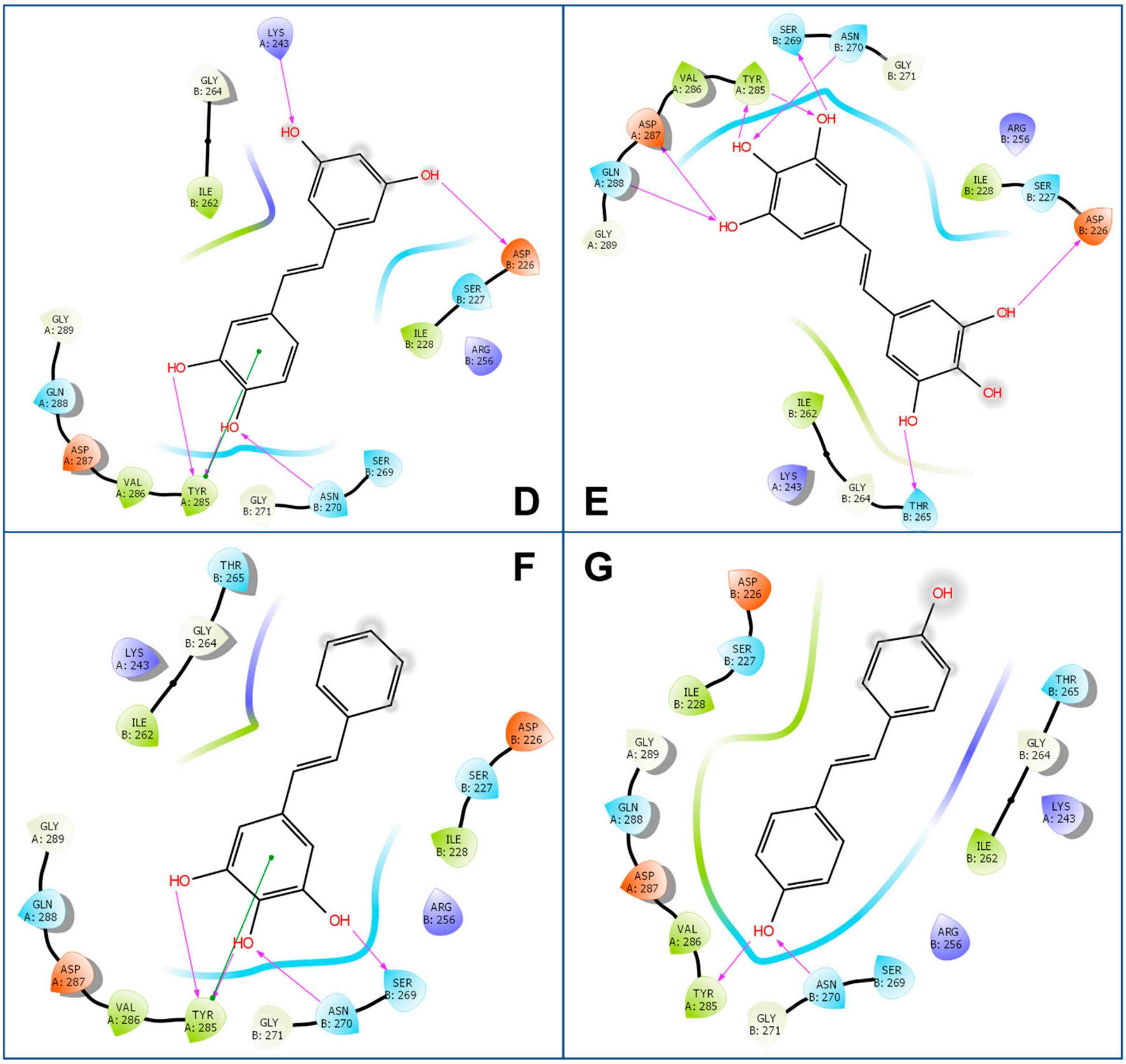

Figure S17 2-D protein-ligand interaction diagrams of S site on 5TUS and four ligands: Piceatannol (D), M8 (E), 3,4,5-THS (F) and 4,4′-DHS (G). The purple arrow indicates the hydrogen bond; the green line indicates the π-π stacking.

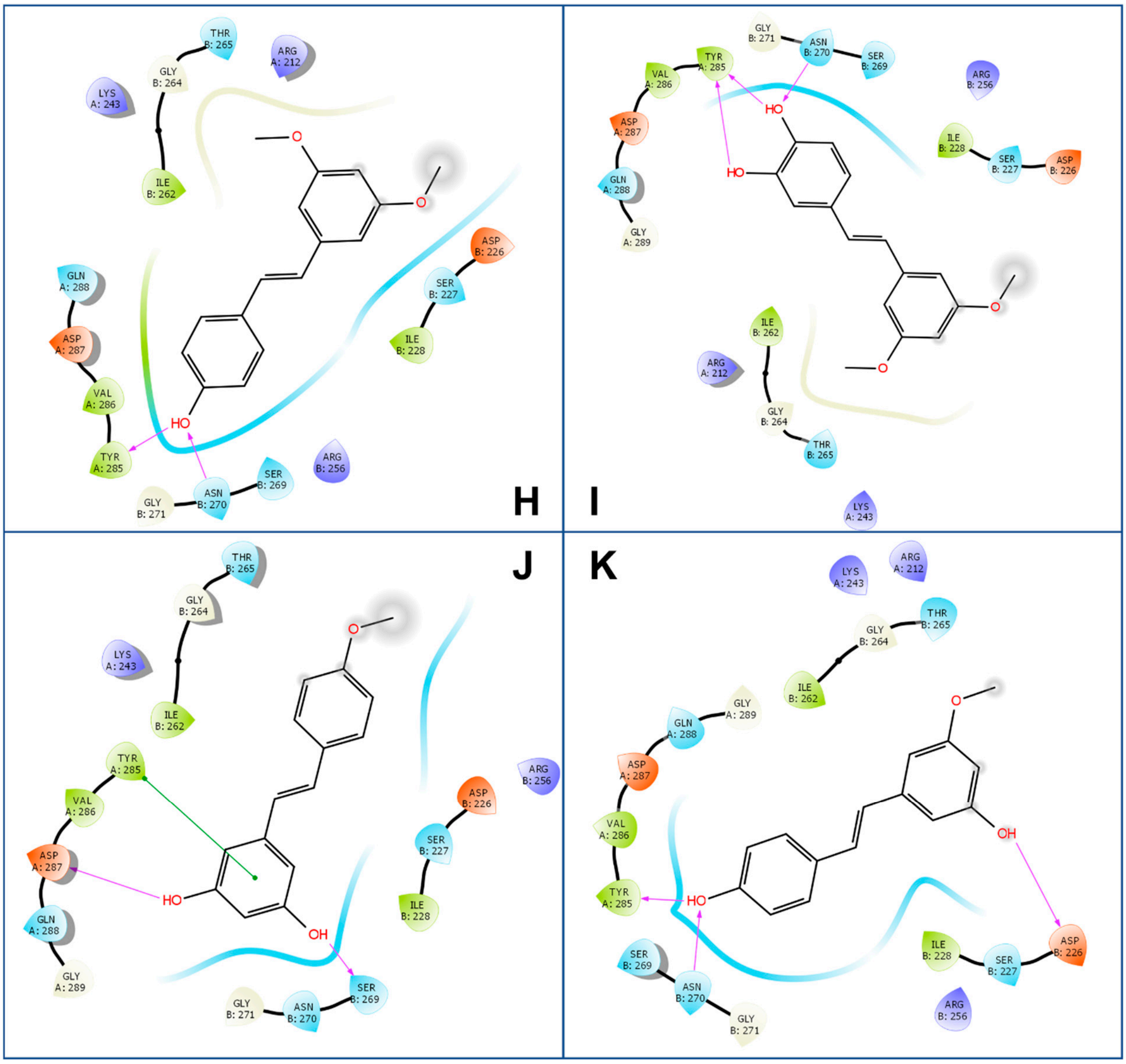

Figure S18 2-D protein-ligand interaction diagrams of S site on 5TUS and four ligands: Pterostilbene (H), HPSB (I), DRG (J), and Pinostilbene (K). The purple arrow indicates the hydrogen bond; the green line indicates the π-π stacking.

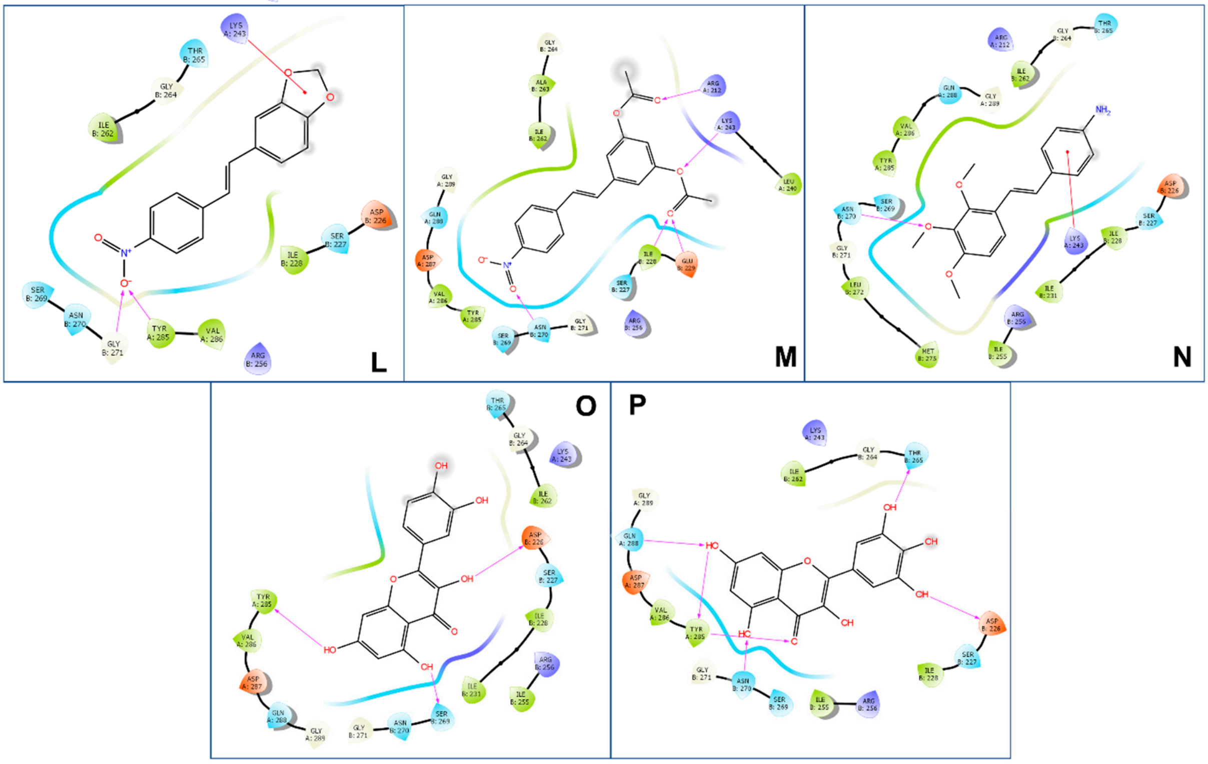

Figure S19 2-D protein-ligand interaction diagrams of S site on 5TUS and five ligands: Compound-1 (L), Compound-2 (M), Compound-3 (N), Quercetin (O), and Myricetin (P). The purple arrow indicates the hydrogen bond; the green line indicates the π-π stacking.

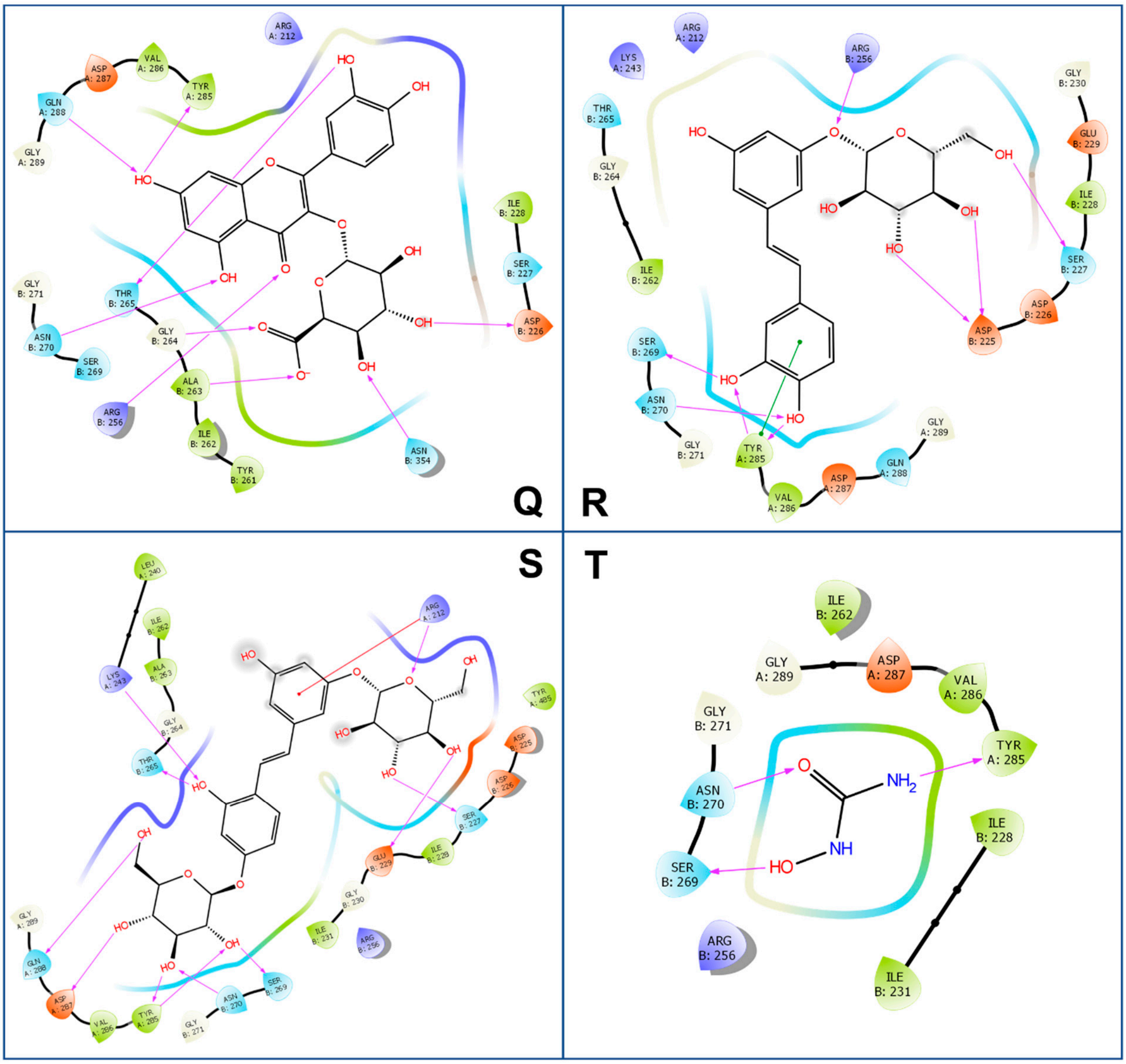

Figure S20 2-D protein-ligand interaction diagrams of S site on 5TUS and four ligands: Miquelianin (Q), Astringin (R), Mulberroside A (S), and Hydroxyurea (T). The purple arrow indicates the hydrogen bond; the green line indicates the π-π stacking, the red line indicates the π-cation.

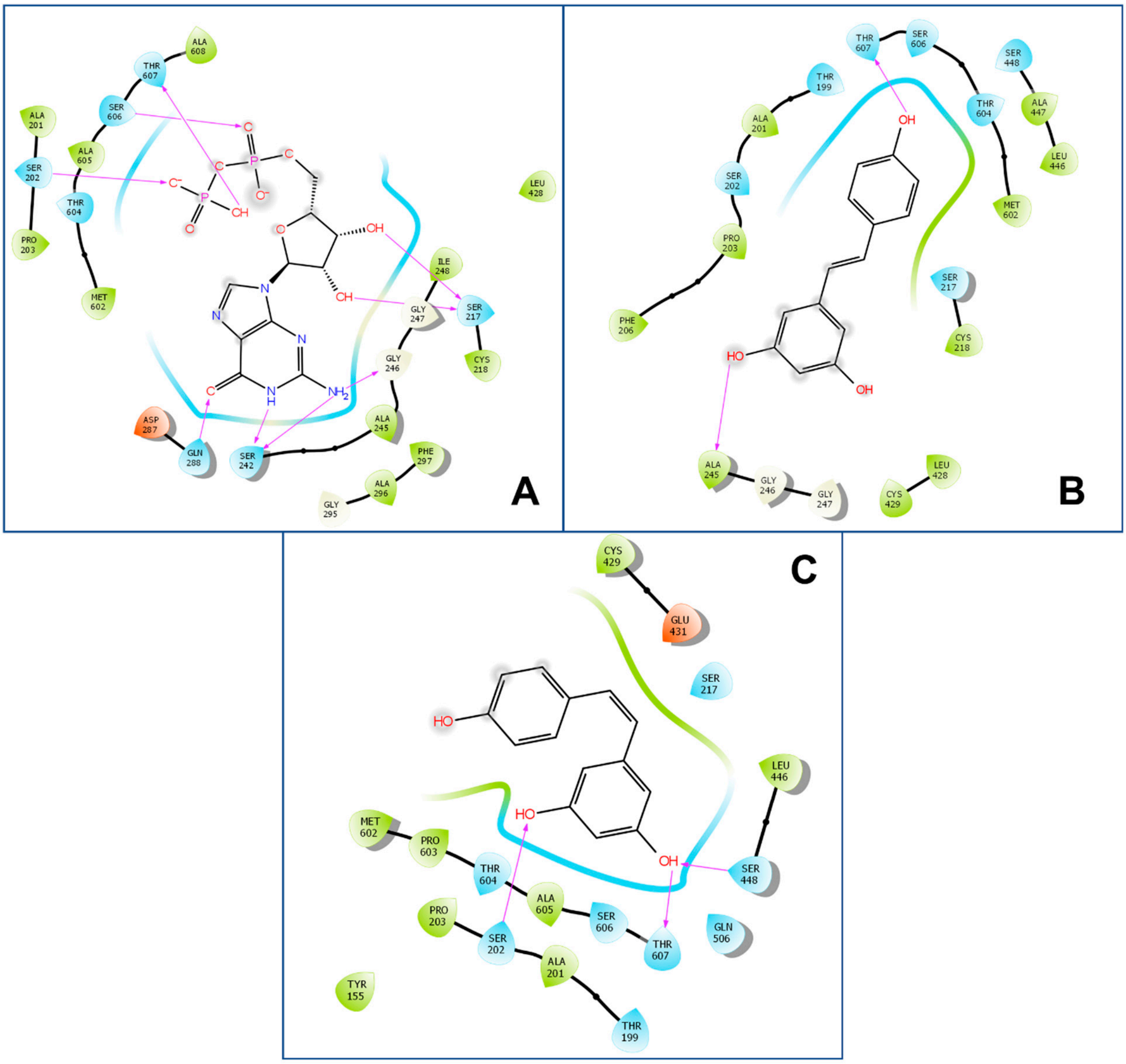

Figure S21 2-D protein-ligand interaction diagrams of C site on 5TUS and three ligands: GDP (A), *Trans*-resveratrol (B), and *Cis*-resveratrol (C). The purple arrow indicates the hydrogen bond.

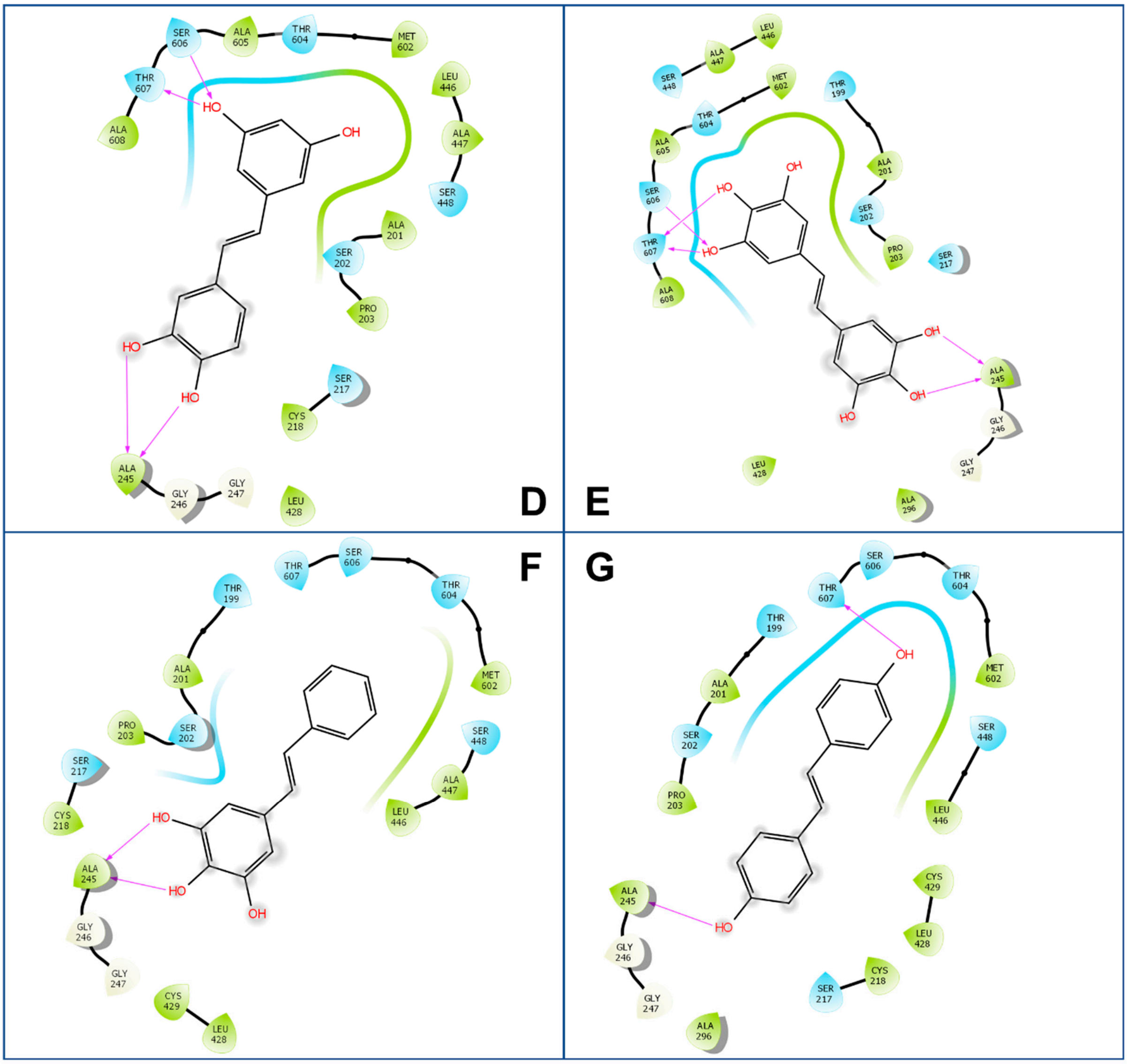

Figure S22 2-D protein-ligand interaction diagrams of C site on 5TUS and four ligands: Piceatannol (D), M8 (E), 3,4,5-THS (F) and 4,4′-DHS (G). The purple arrow indicates the hydrogen bond.

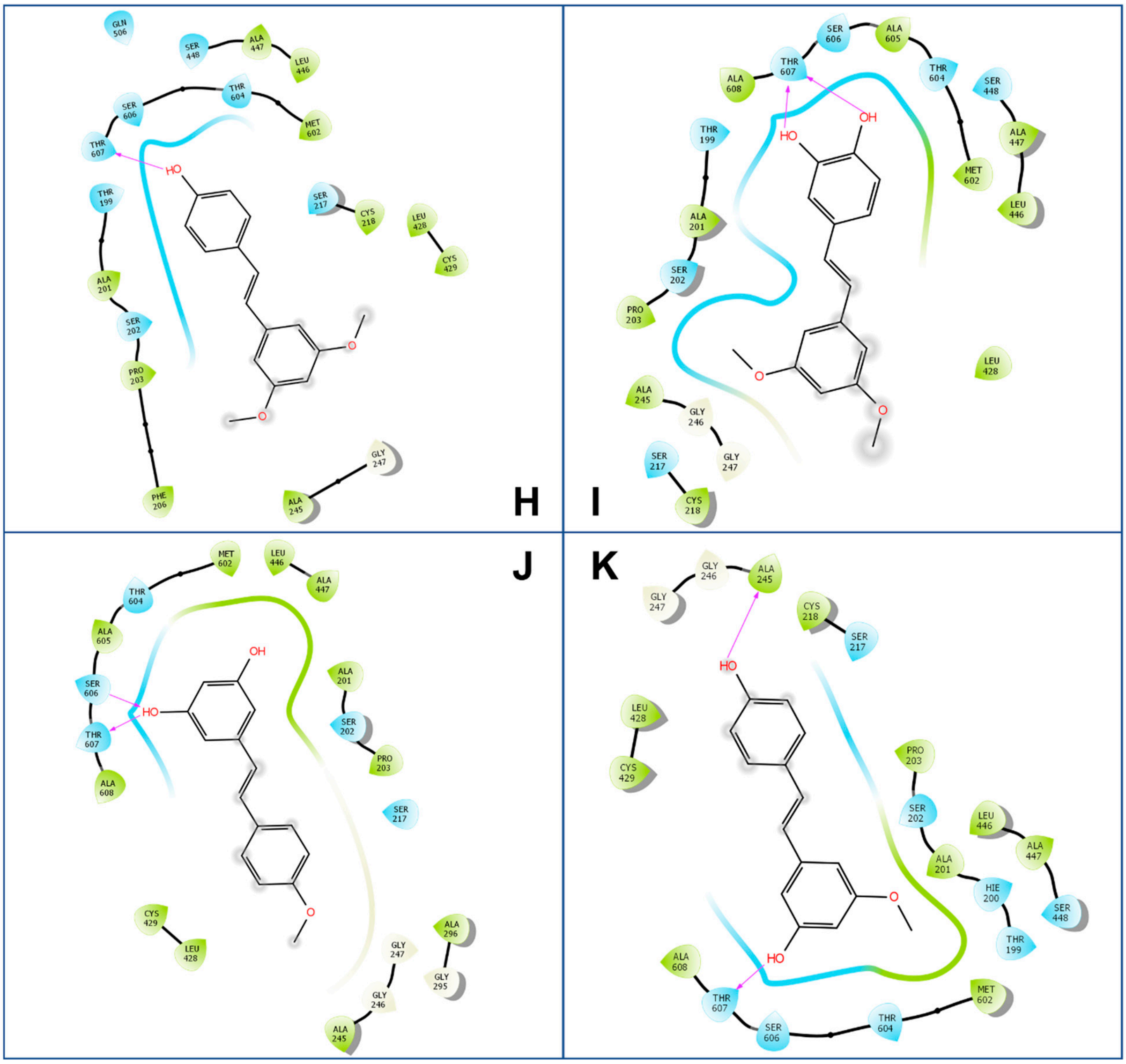

Figure S23 2-D protein-ligand interaction diagrams of C site on 5TUS and four ligands: Pterostilbene (H), HPSB (I), DRG (J), and Pinostilbene (K). The purple arrow indicates the hydrogen bond.

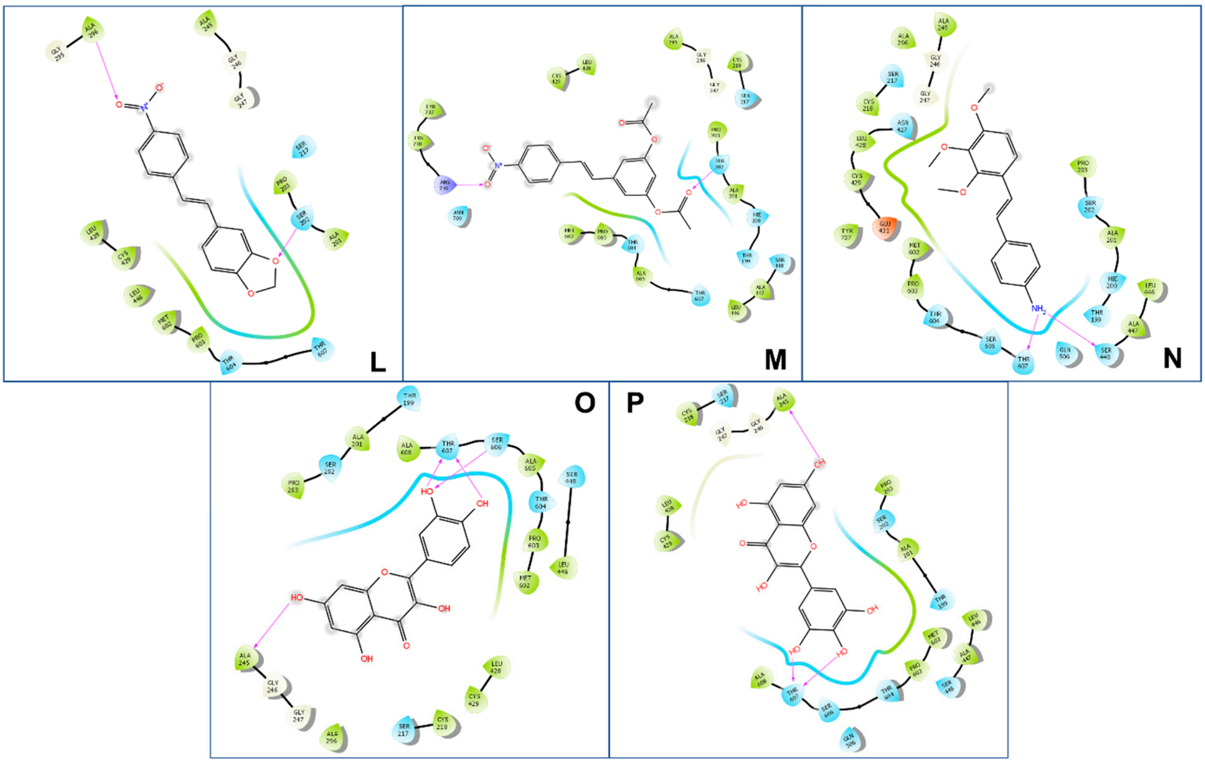

Figure S24 2-D protein-ligand interaction diagrams of C site on 5TUS and five ligands: Compound-1 (L), Compound-2 (M), Compound-3 (N), Quercetin (O), and Myricetin (P). The purple arrow indicates the hydrogen bond.

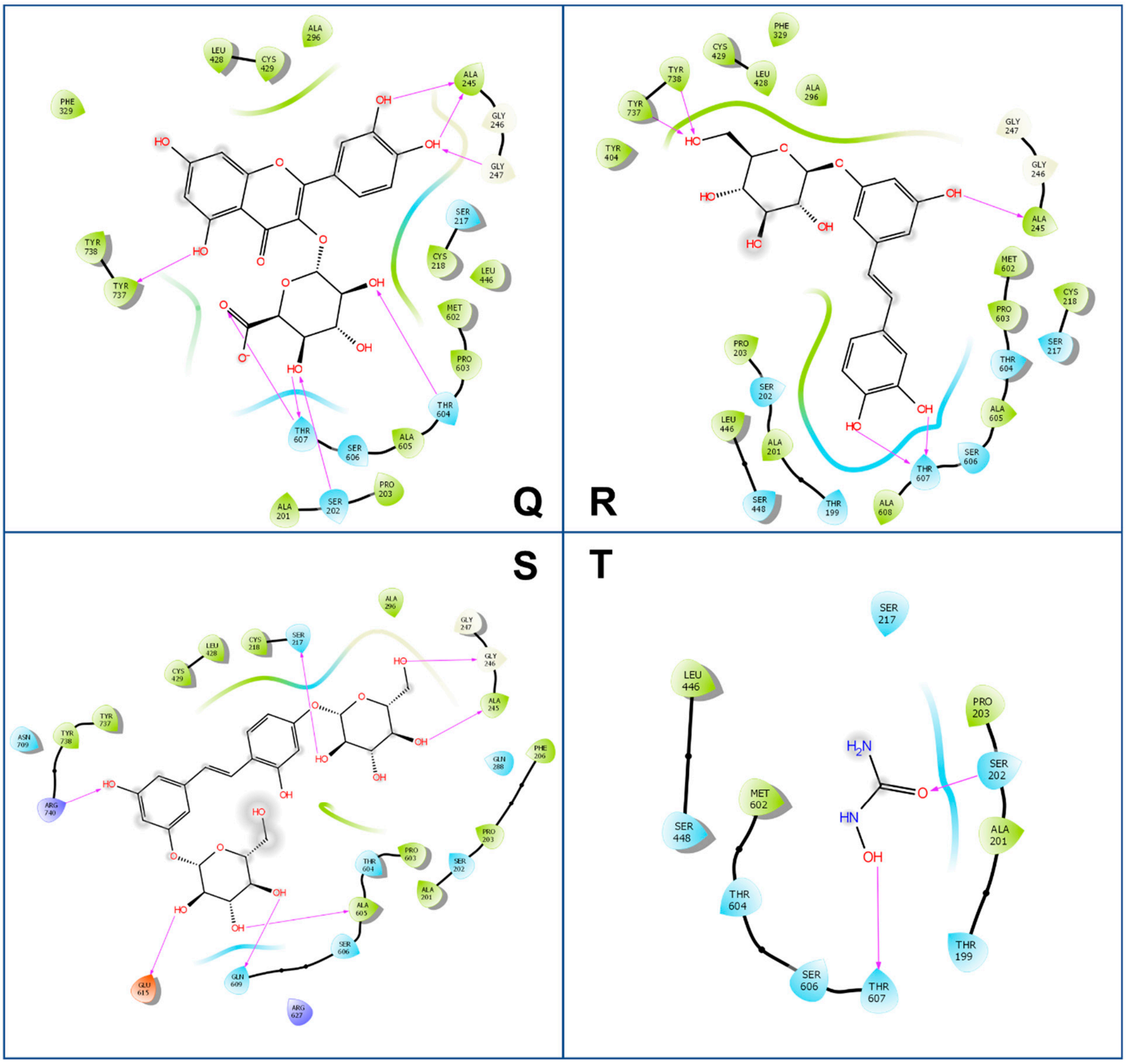

Figure S25 2-D protein-ligand interaction diagrams of C site on 5TUS and four ligands: Miquelianin (Q), Astringin (R), Mulberroside A (S), and Hydroxyurea (T). The purple arrow indicates the hydrogen bond.

Table S1 The MM-GBSA energies (kcal/mol) of the ligands bound to RNA polymerase (PDB ID: 5IYD) and DNA polymerase δ (predicted model).

**Inhibitor**[#]	5IYD	δ-model
Substrate	-14.92(GTP)	-54.46(dCTP)
Trans-resveratrol	-37.14	-45.31
Piceatannol	-20.42	-44.91
M8	-20.23	-36.16
3,4,5-THS	-16.41	-25.34
4,4′-DHS	-22.36	-28.88
Pterostilbene	-23.85	-30.89
HPSB	-38.22	-31.23
DRG	-23.10	-25.58
Miquelianin	-34.88	-74.49
Quercetin	-29.43	-43.17
Astringin	-20.52	-51.02

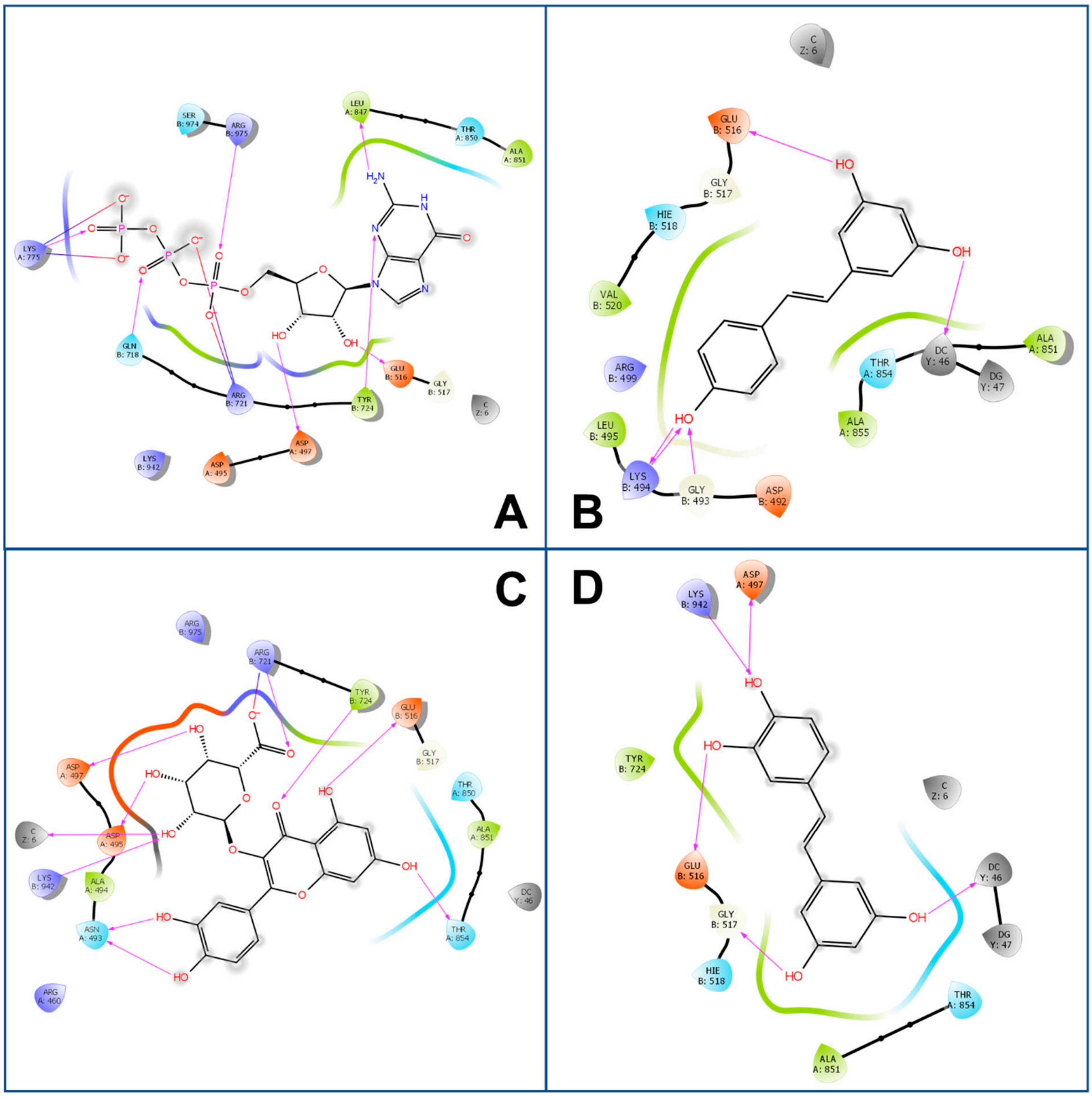

Figure S26 2-D protein-ligand interaction diagrams of 5IYD and four ligands: GTP (A), Resveratrol (B), Miquelianin (C), and Piceatannol (D). The purple arrow indicates the hydrogen bond; the blue-red line indicates the salt bridge.

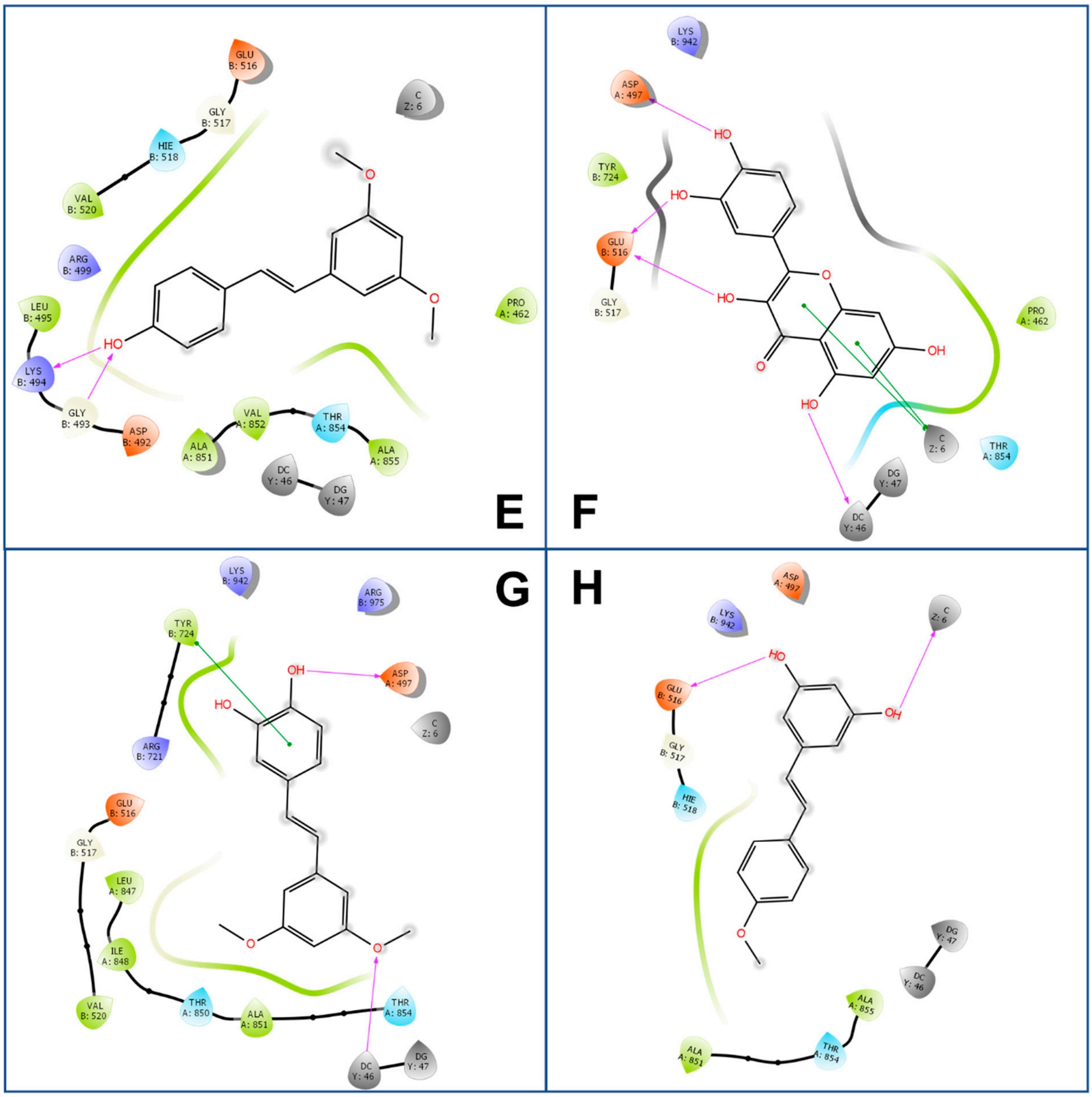

Figure S27 2-D protein-ligand interaction diagrams of 5IYD and four ligands: Pterostilbene (E), Quercetin (F), HPSB (G), and DRG (H). The purple arrow indicates the hydrogen bond; the green line indicates the π-π stacking.

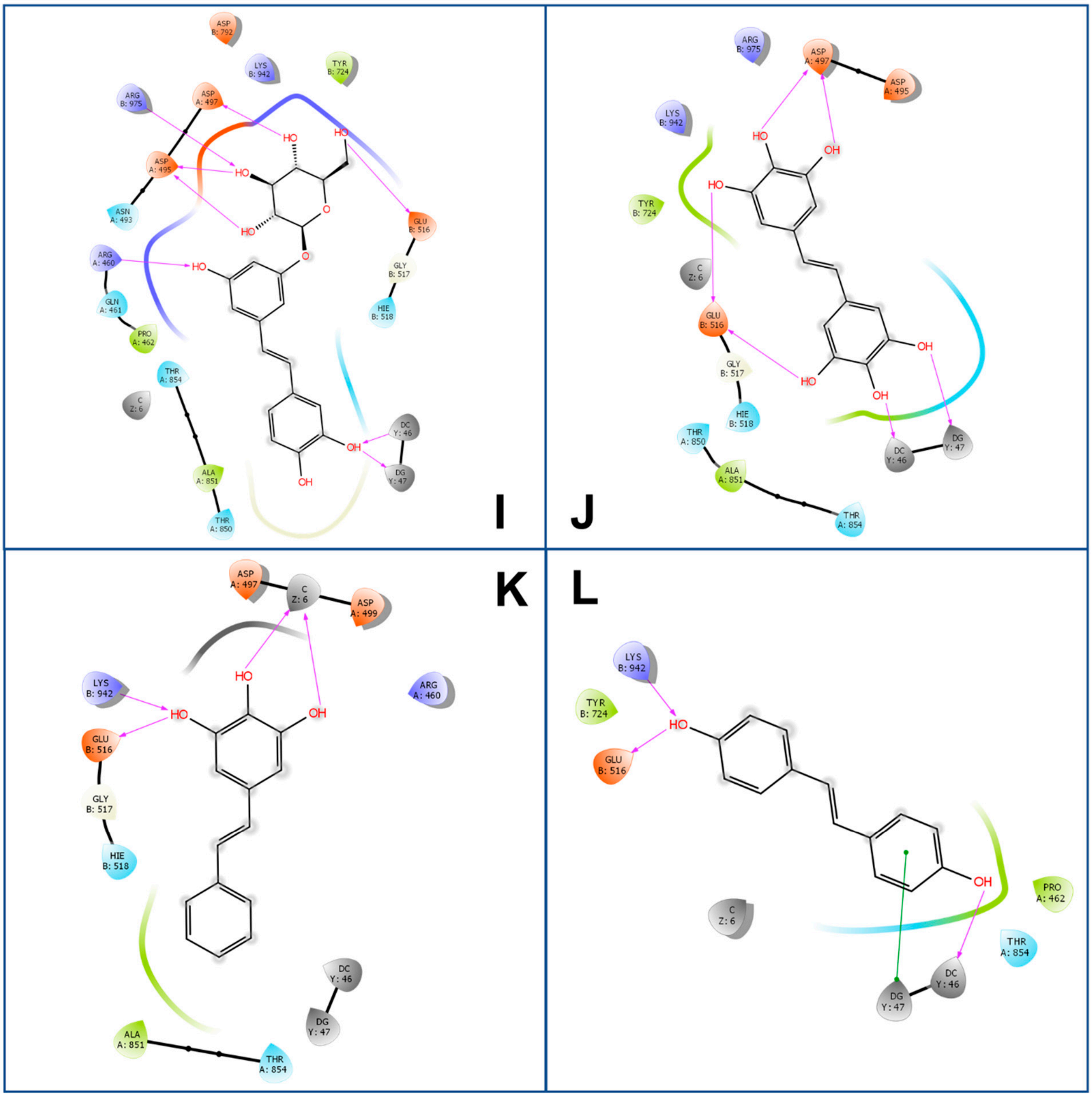

Figure S28 2-D protein-ligand interaction diagrams of 5IYD and four ligands: Astringin (I), M8 (J), 3,4,5-THS (K), and 4,4′-DHS (L). The purple arrow indicates the hydrogen bond; the green line indicates the π-π stacking.

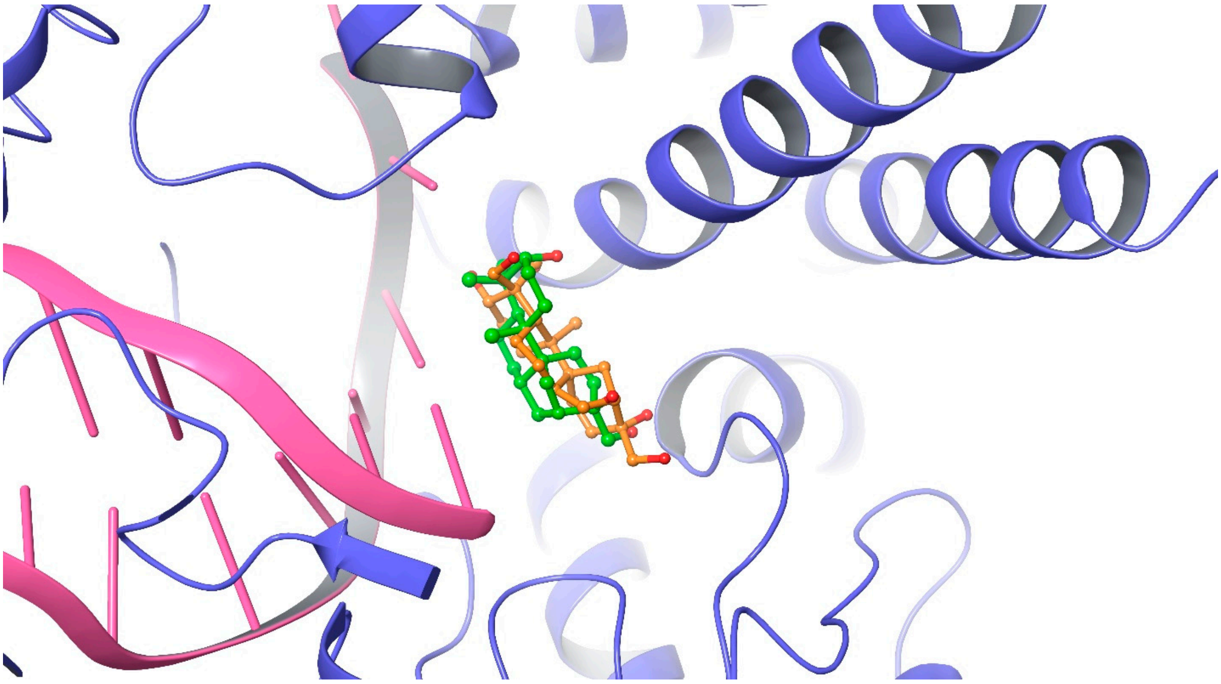

Figure S29 Superposition of docked aphidicolin (yellow) and original aphidicolin (green) on DNA polymerase α (PDB ID: 4Q5V).

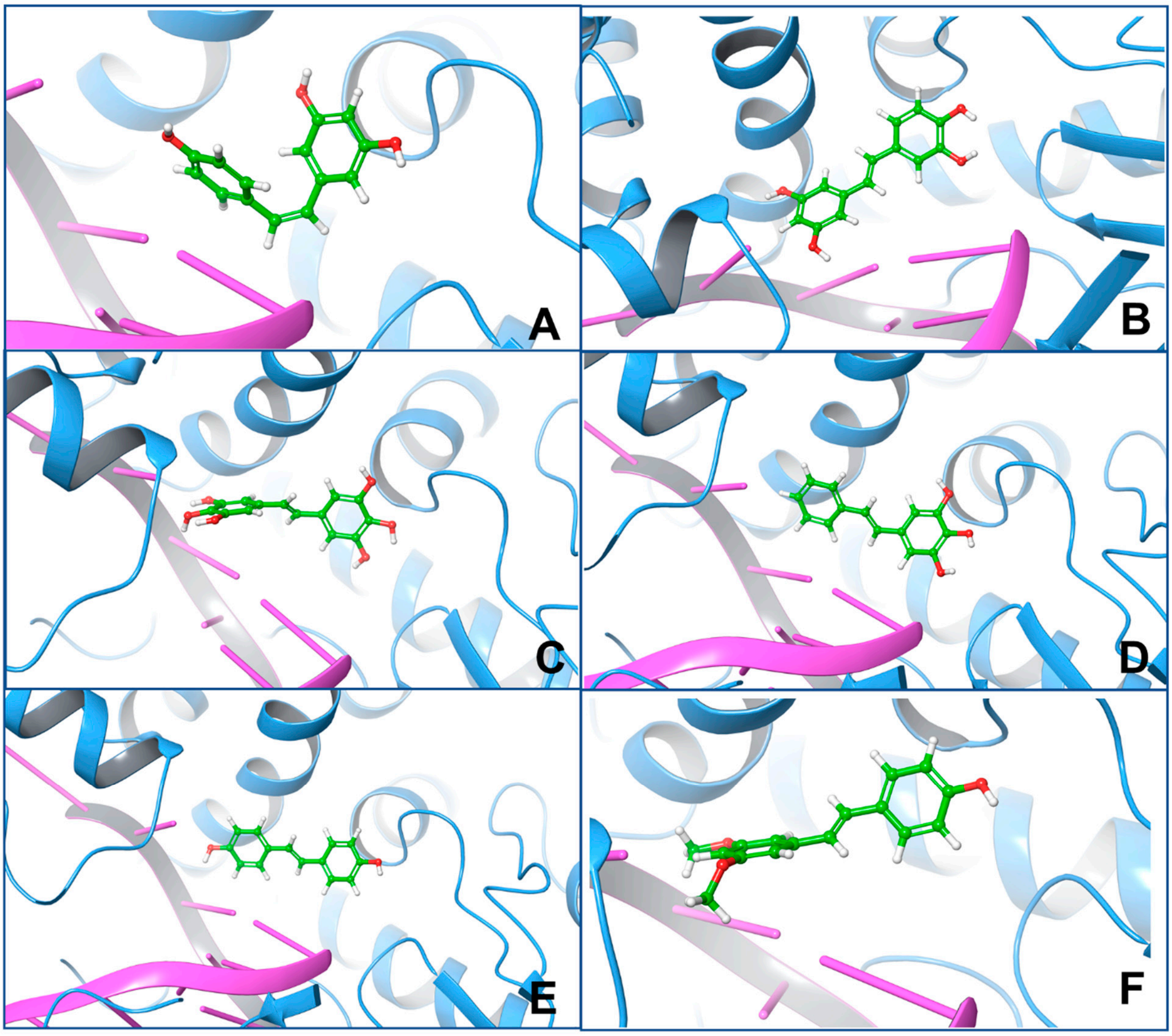

Figure S30 Docking poses of 4Q5V and six ligands: *Cis*-resveratrol (A), Piceatannol (B), M8 (C), 3,4,5-THS (D), 4,4′-DHS (E), and Pterostilbene (F).

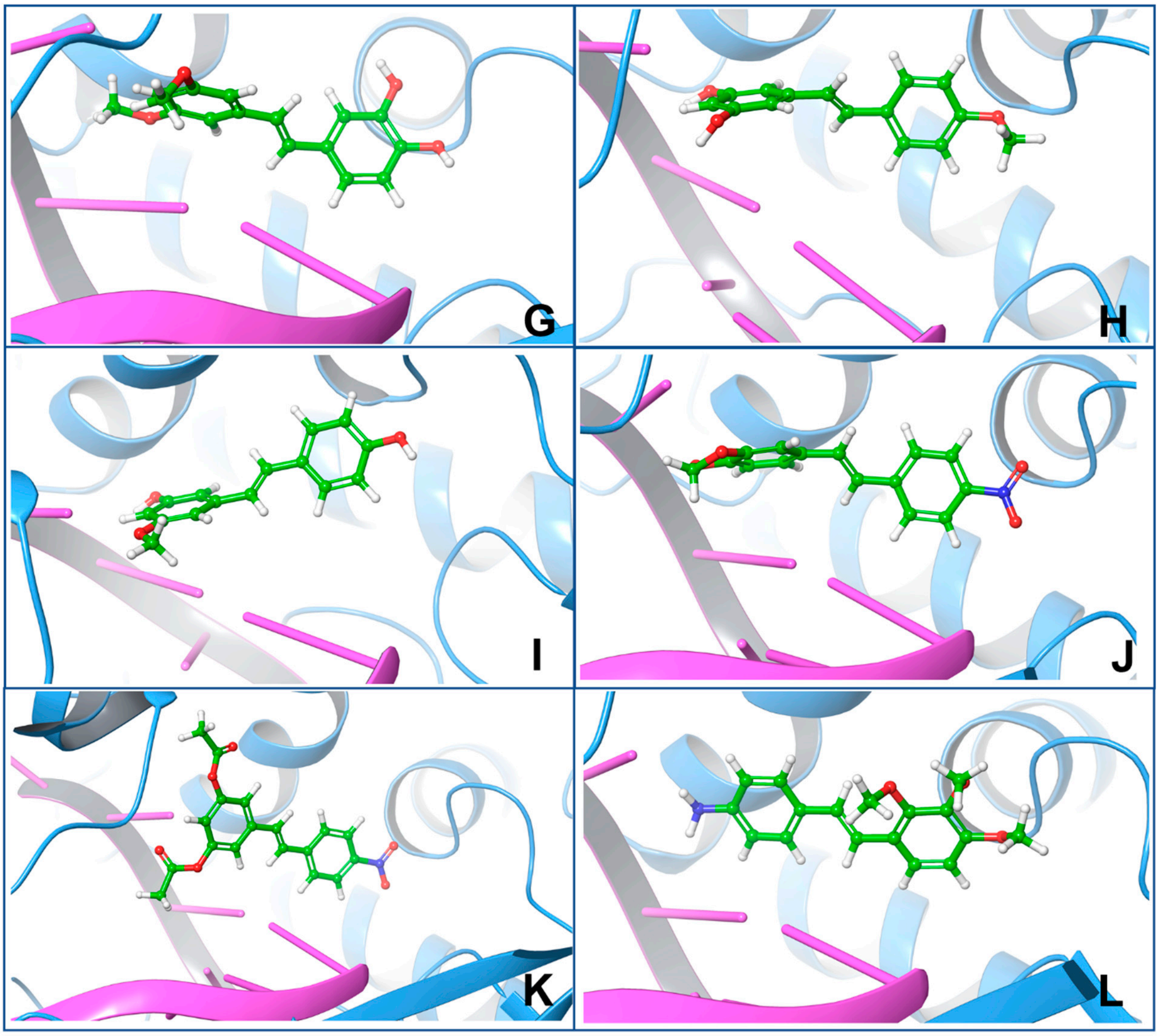

Figure S31 Docking poses of 4Q5V and six ligands: HPSB (G), DRG (H), Pinostilbene (I), Compound-1 (J), Compound-2 (K), and Compound-3 (L).

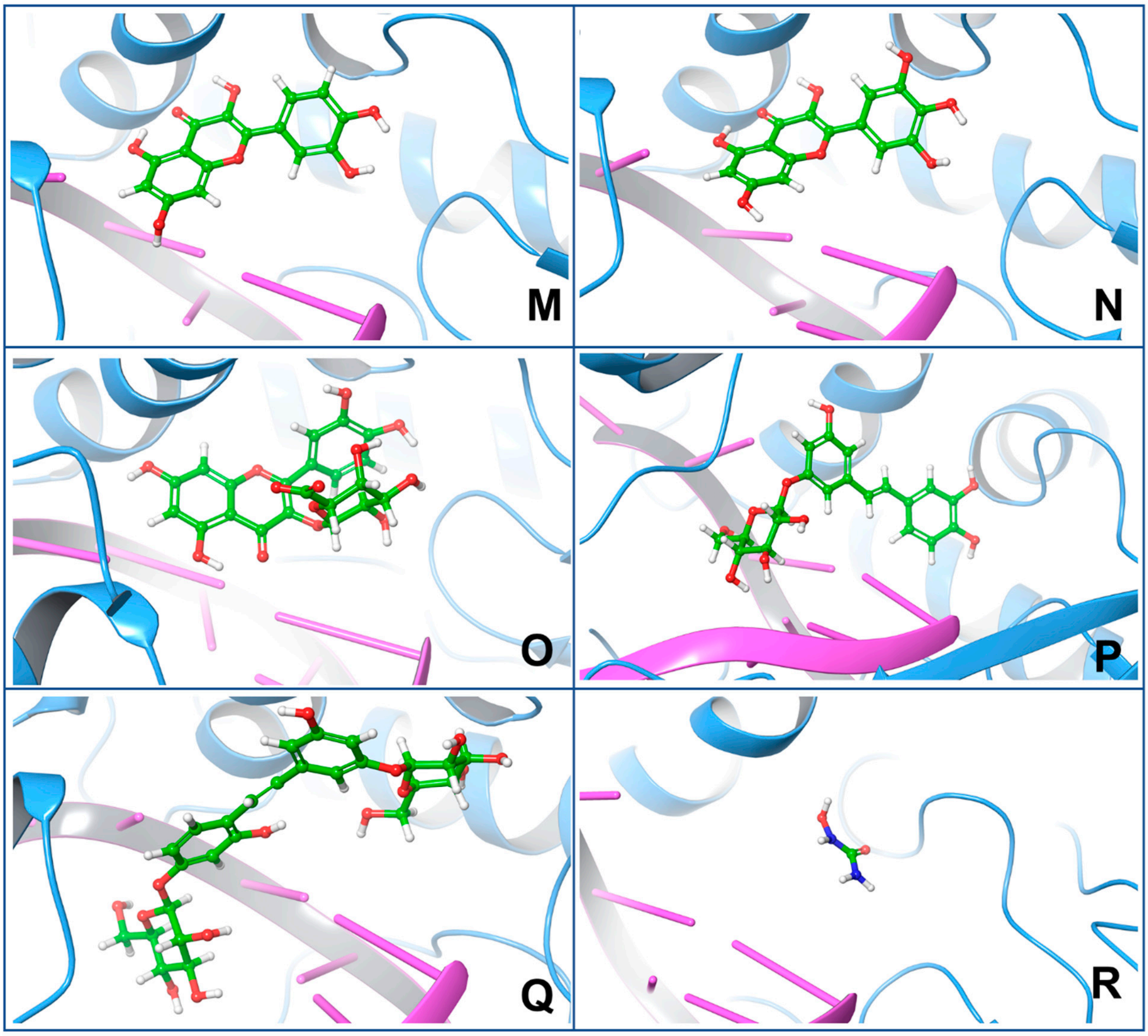

Figure S32 Docking poses of 4Q5V and six ligands: Quercetin (M), Myricetin (N), Astringin (O), Miquelianin (P), Mulberroside A (Q), and Hydroxyurea (R).

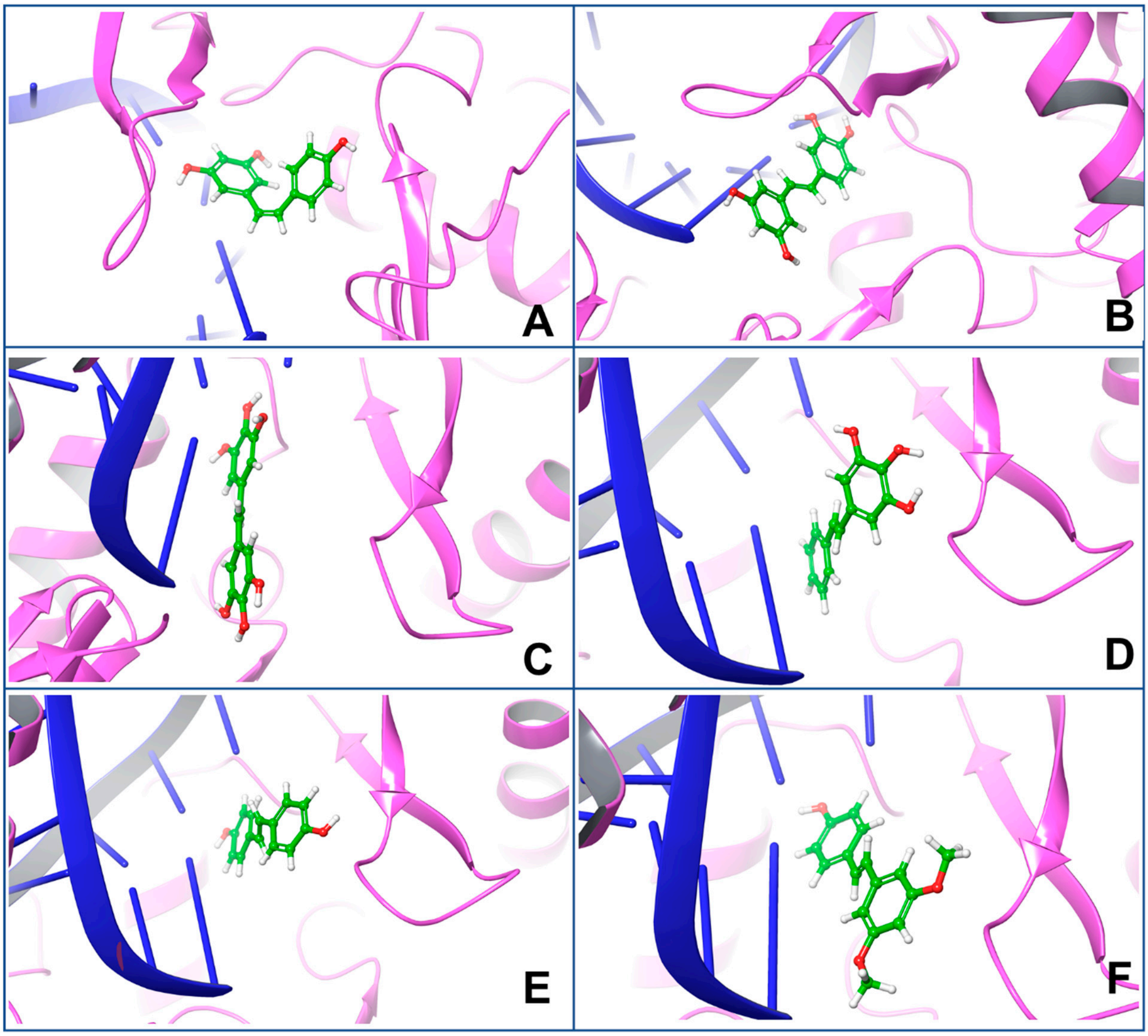

Figure S33 Docking poses of 5TXM and six ligands: *Cis*-resveratrol (A), Piceatannol (B), M8 (C), 3,4,5-THS (D), 4,4′-DHS (E), and Pterostilbene (F).

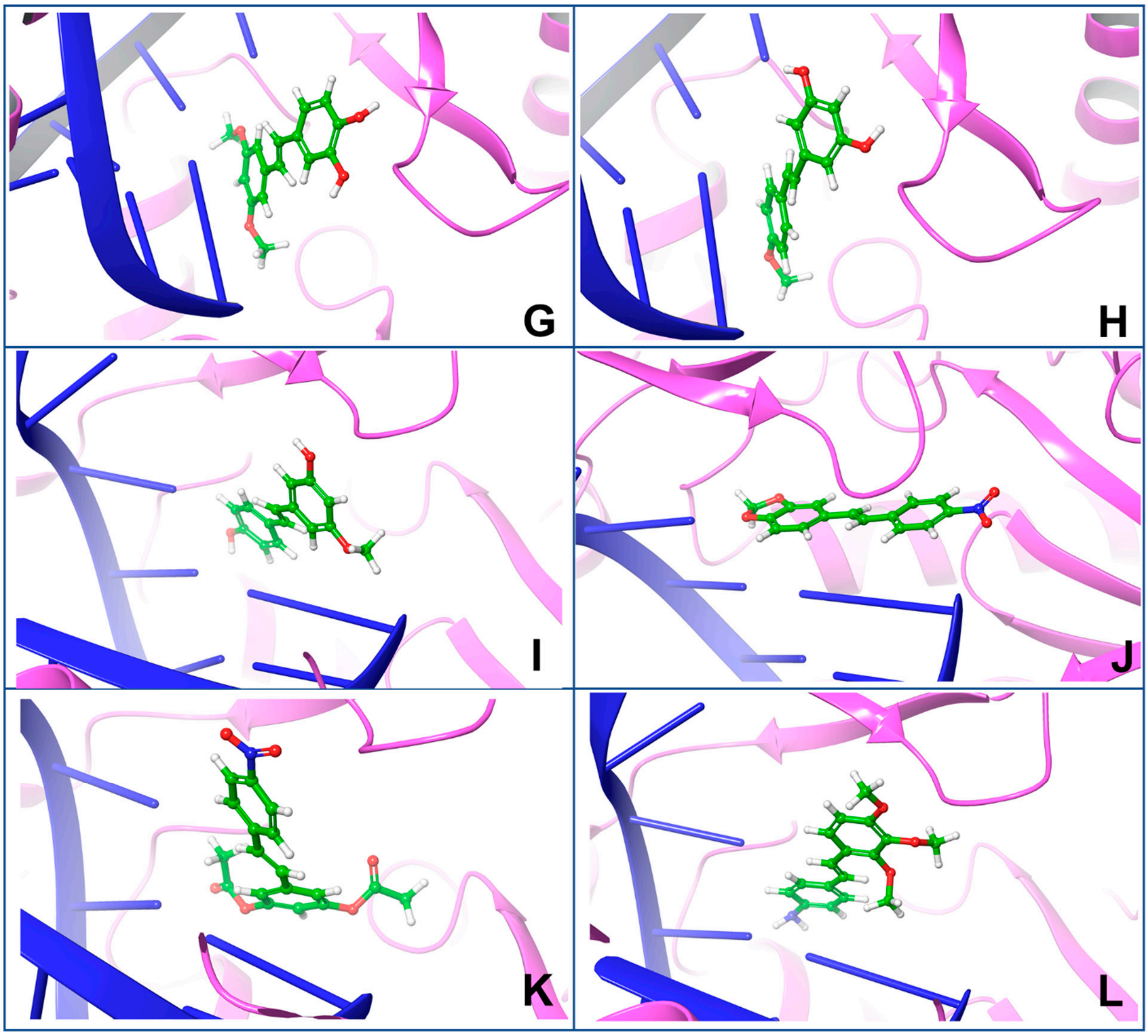

Figure S34 Docking poses of 5TXM and six ligands: HPSB (G), DRG (H), Pinostilbene (I), Compound-1 (J), Compound-2 (K), and Compound-3 (L).

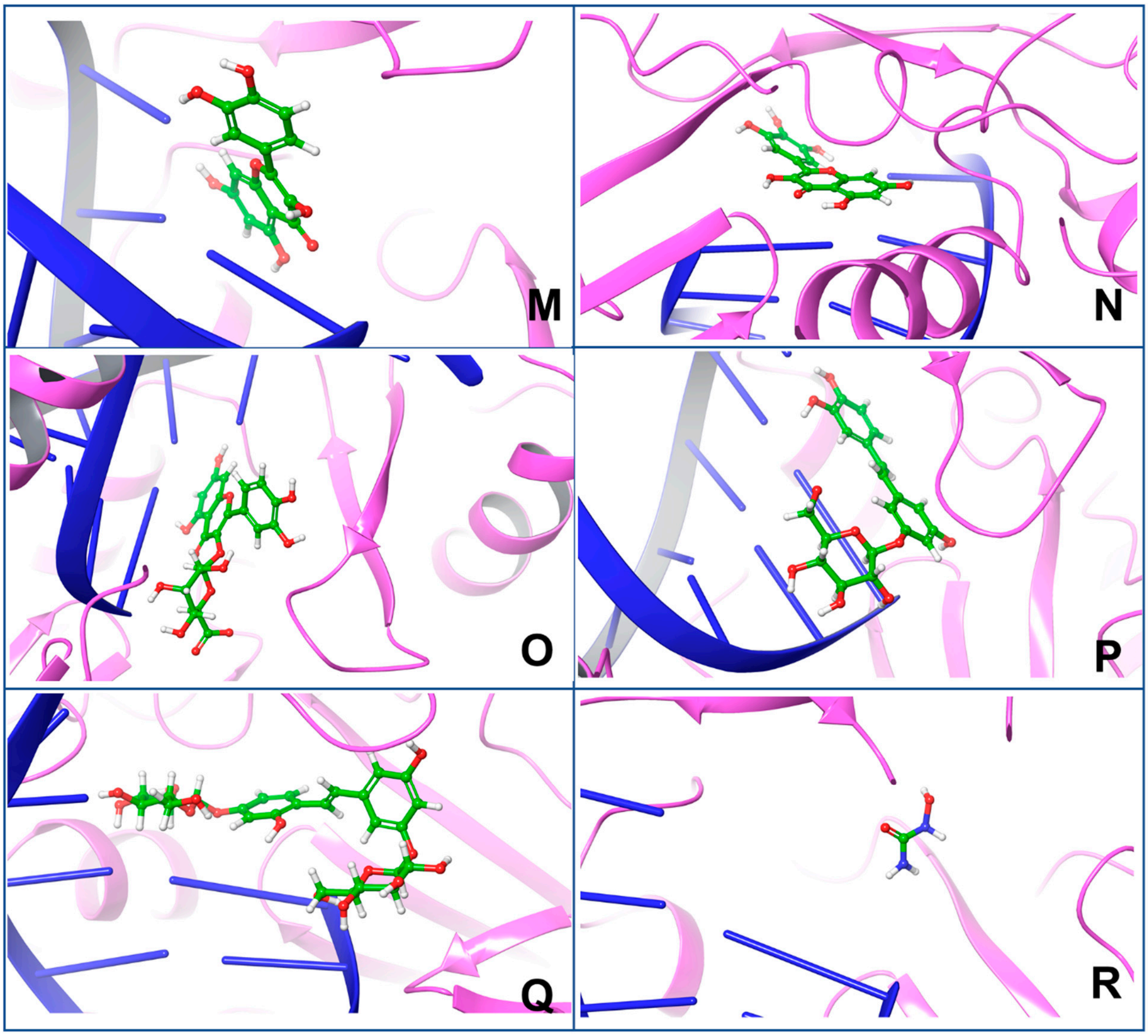

Figure S35 Docking poses of 5TXM and six ligands: Quercetin (M), Myricetin (N), Astringin (O), Miquelianin (P), Mulberroside A (Q), and Hydroxyurea (R).

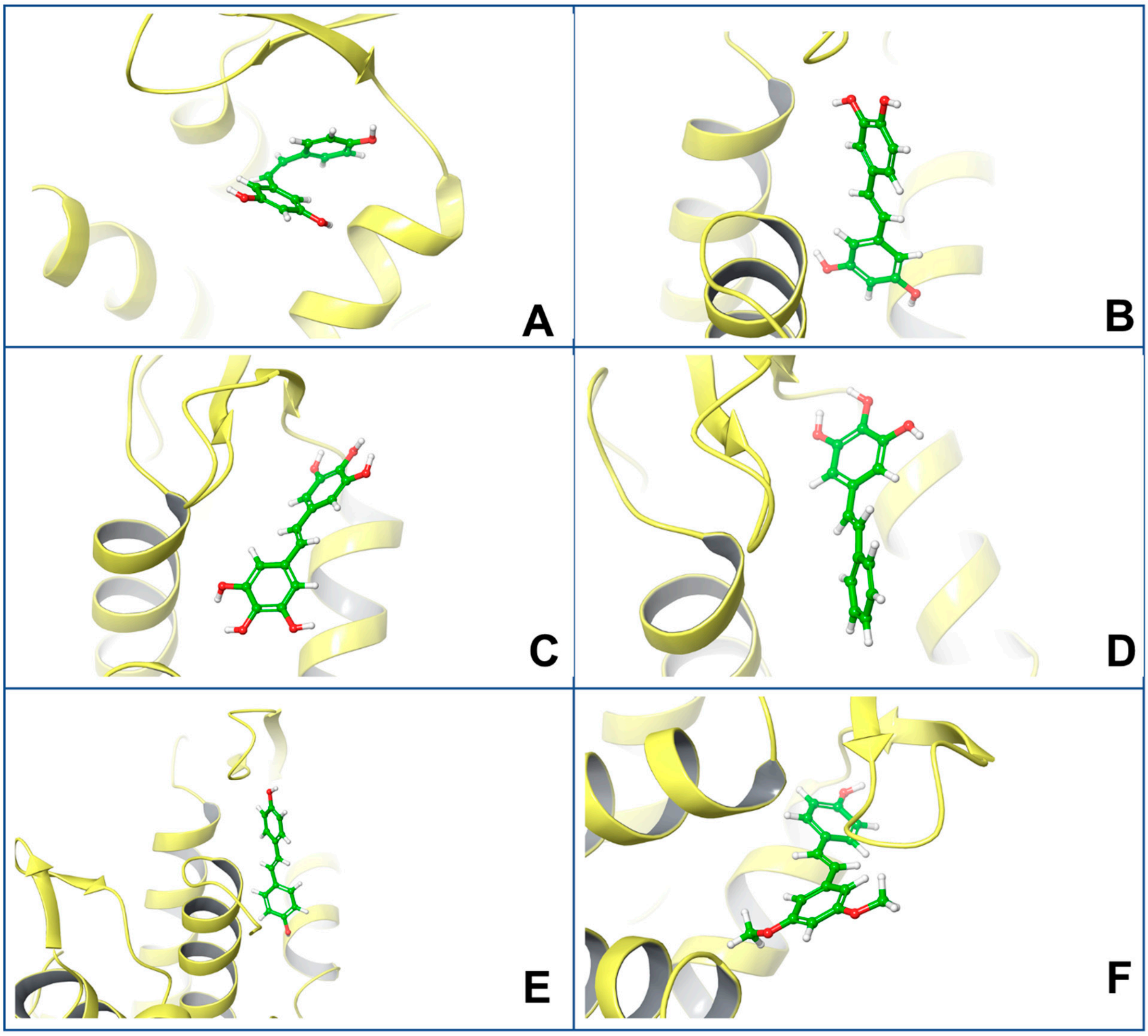

Figure S36 Docking poses of A site on 5TUS and six ligands: *Cis*-resveratrol (A), Piceatannol (B), M8 (C), 3,4,5-THS (D), 4,4′-DHS (E), and Pterostilbene (F).

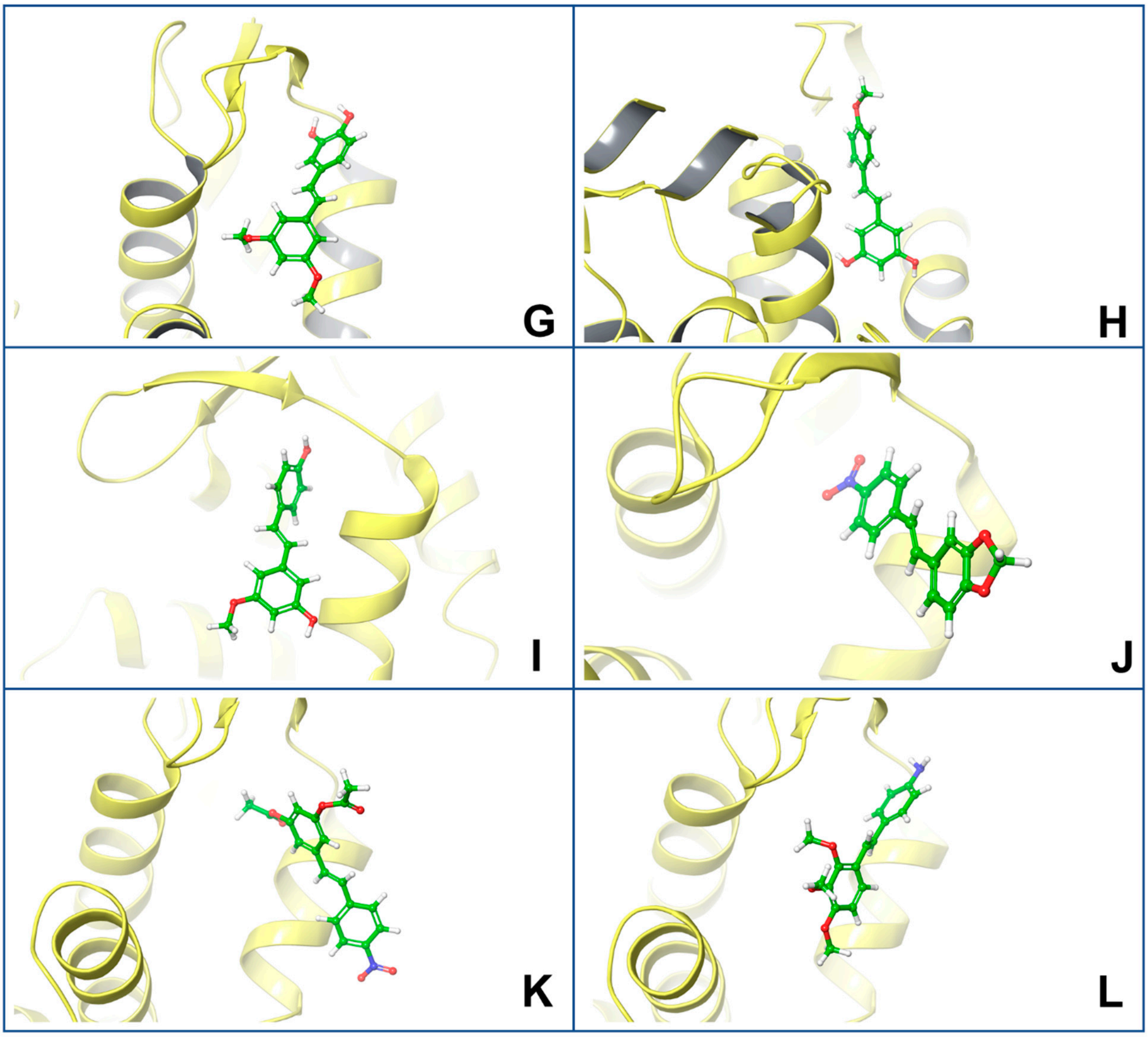

Figure S37 Docking poses of A site on 5TUS and six ligands: HPSB (G), DRG (H), Pinostilbene (I), Compound-1 (J), Compound-2 (K), and Compound-3 (L).

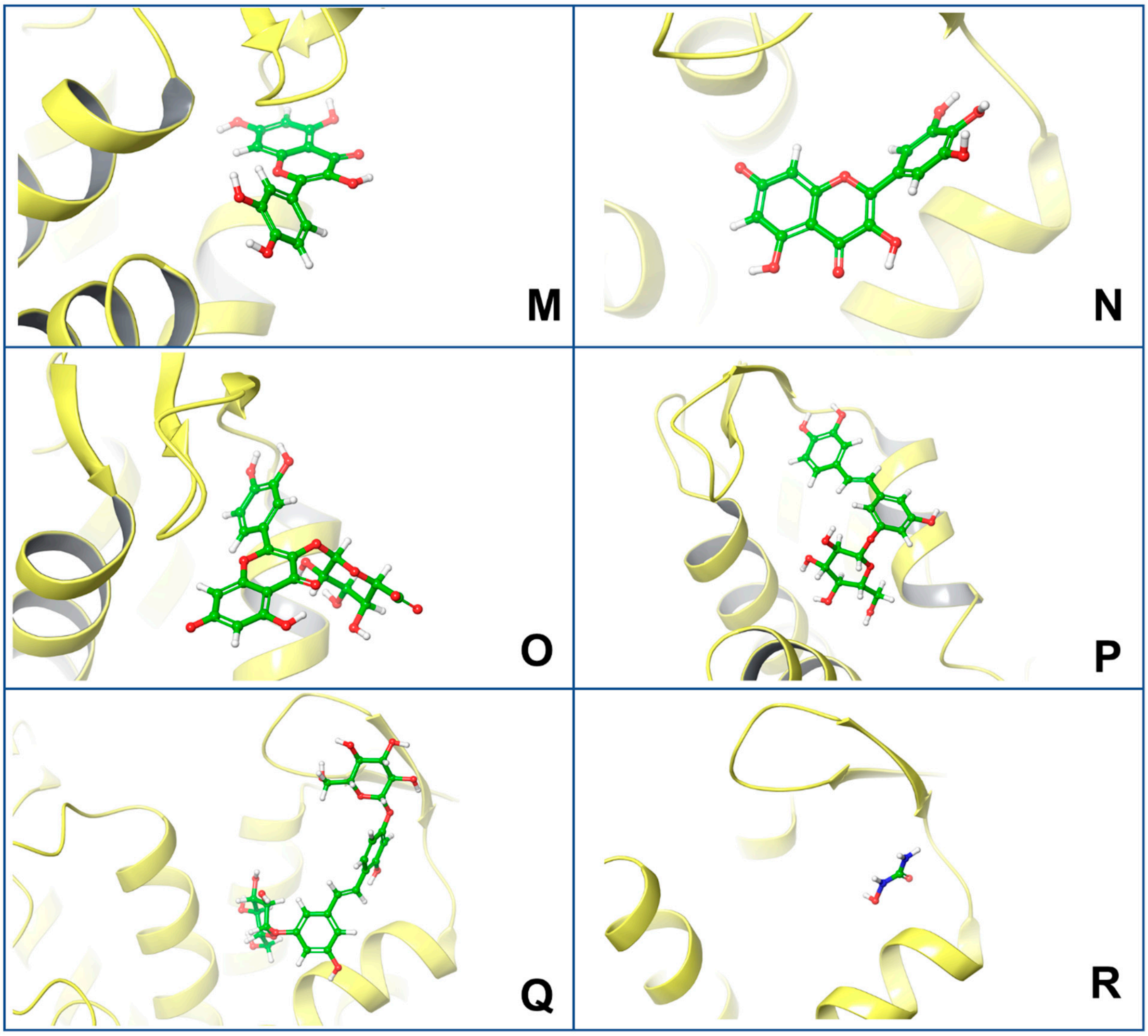

Figure S38 Docking poses of A site on 5TUS and six ligands: Quercetin (M), Myricetin (N), Astringin (O), Miquelianin (P), Mulberroside A (Q), and Hydroxyurea (R).

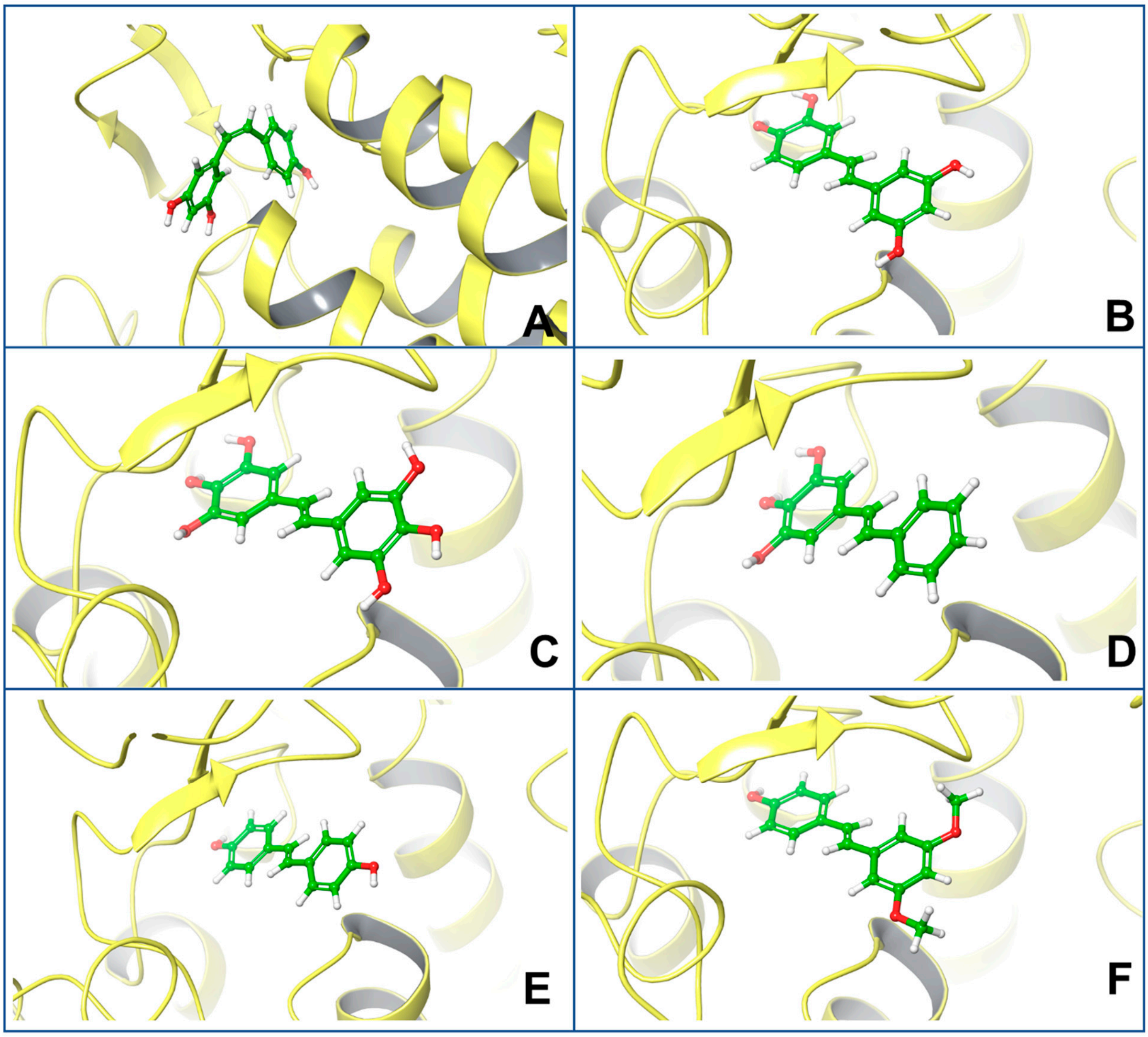

Figure S39 Docking poses of S site on 5TUS and six ligands: ***Cis*-resveratrol (A), Piceatannol (B), M8 (C), 3,4,5-THS (D), 4,4′-DHS (E), and Pterostilbene (F).**

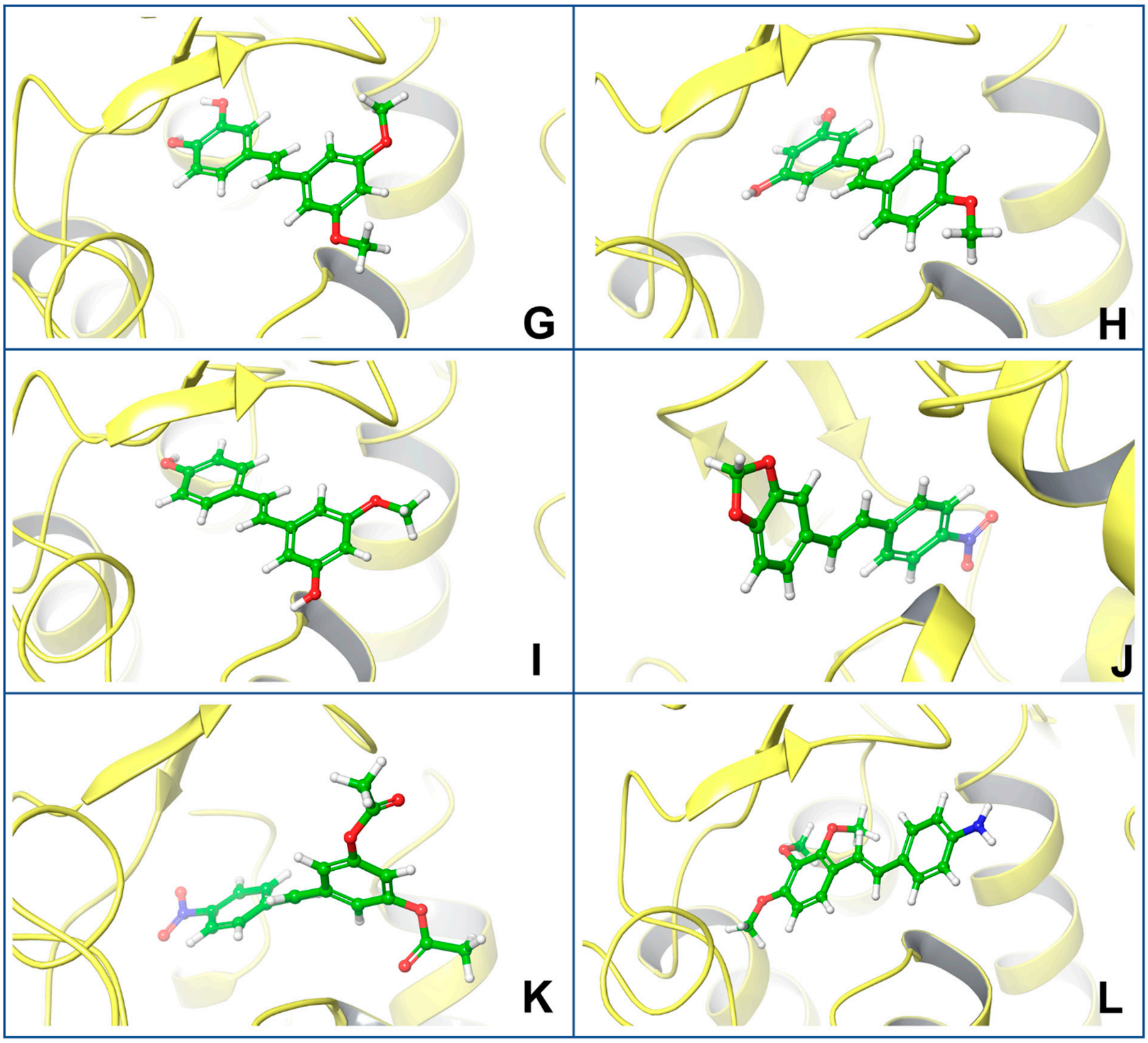

Figure S40 Docking poses of S site on 5TUS and six ligands: HPSB (G), DRG (H), Pinostilbene (I), Compound-1 (J), Compound-2 (K), and Compound-3 (L).

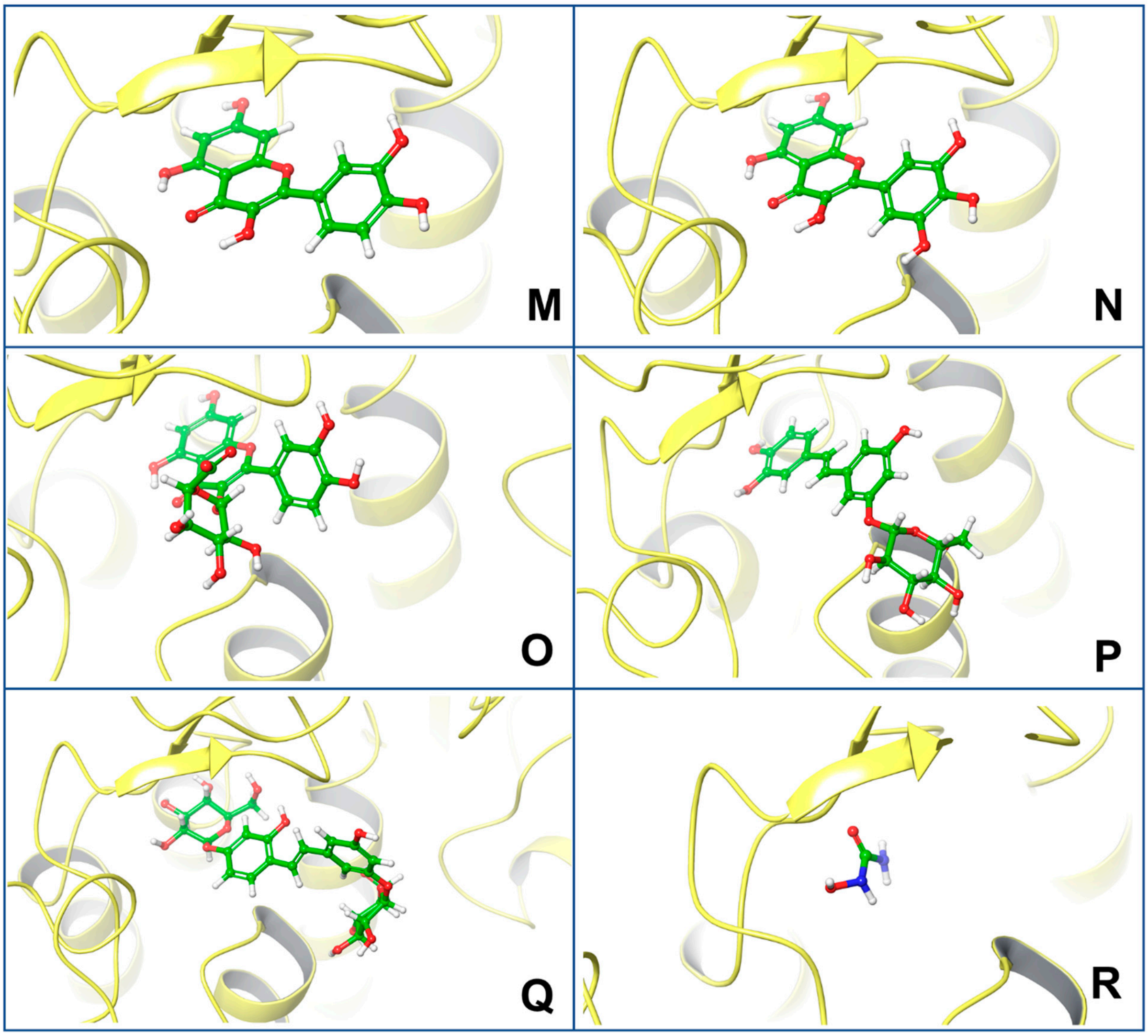

Figure S41 Docking poses of S site on 5TUS and six ligands: Quercetin (M), Myricetin (N), Astringin (O), Miquelianin (P), Mulberroside A (Q), and Hydroxyurea (R).

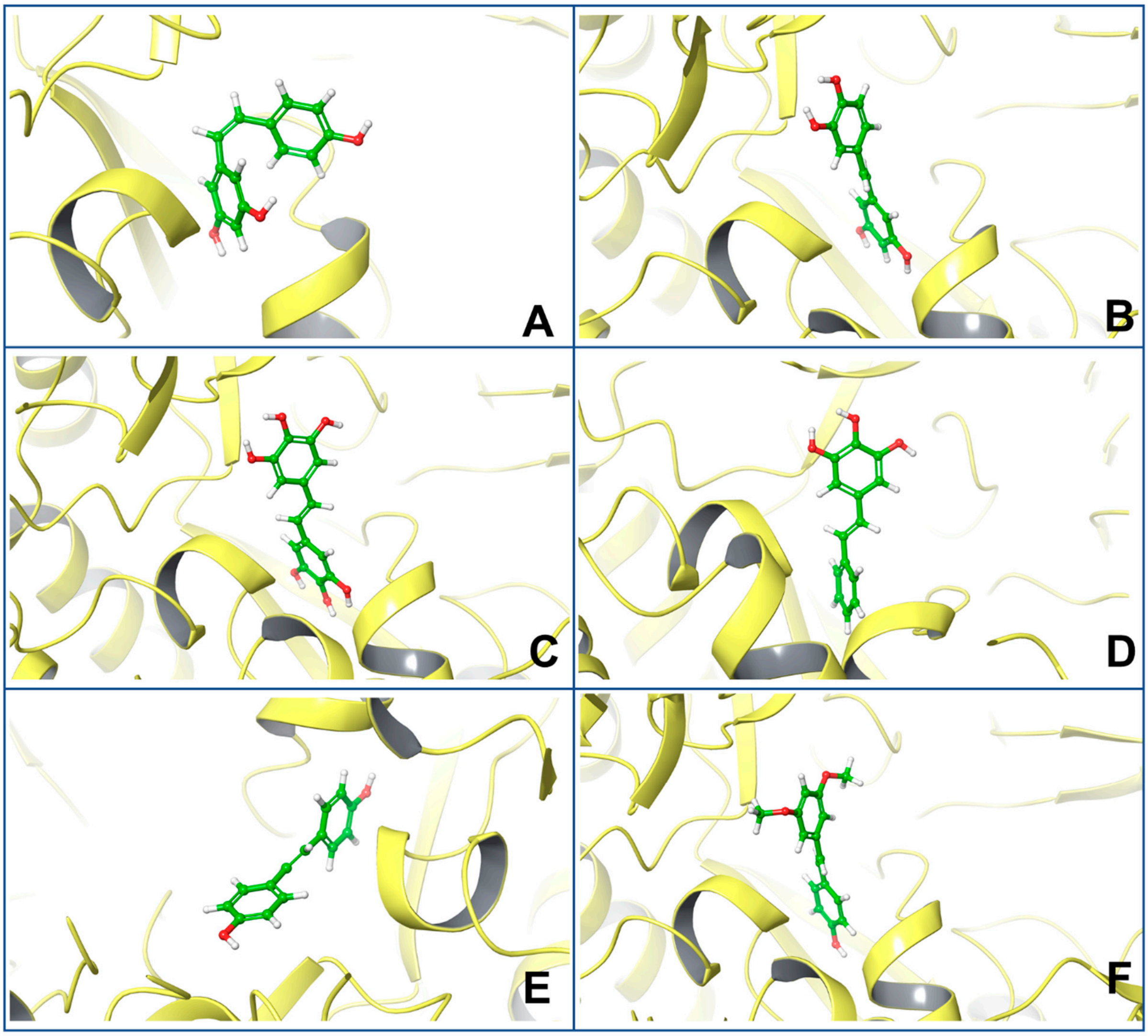

Figure S42 Docking poses of C site on 5TUS and six ligands: *Cis*-resveratrol (A), Piceatannol (B), M8 (C), 3,4,5-THS (D), 4,4′-DHS (E), and Pterostilbene (F).

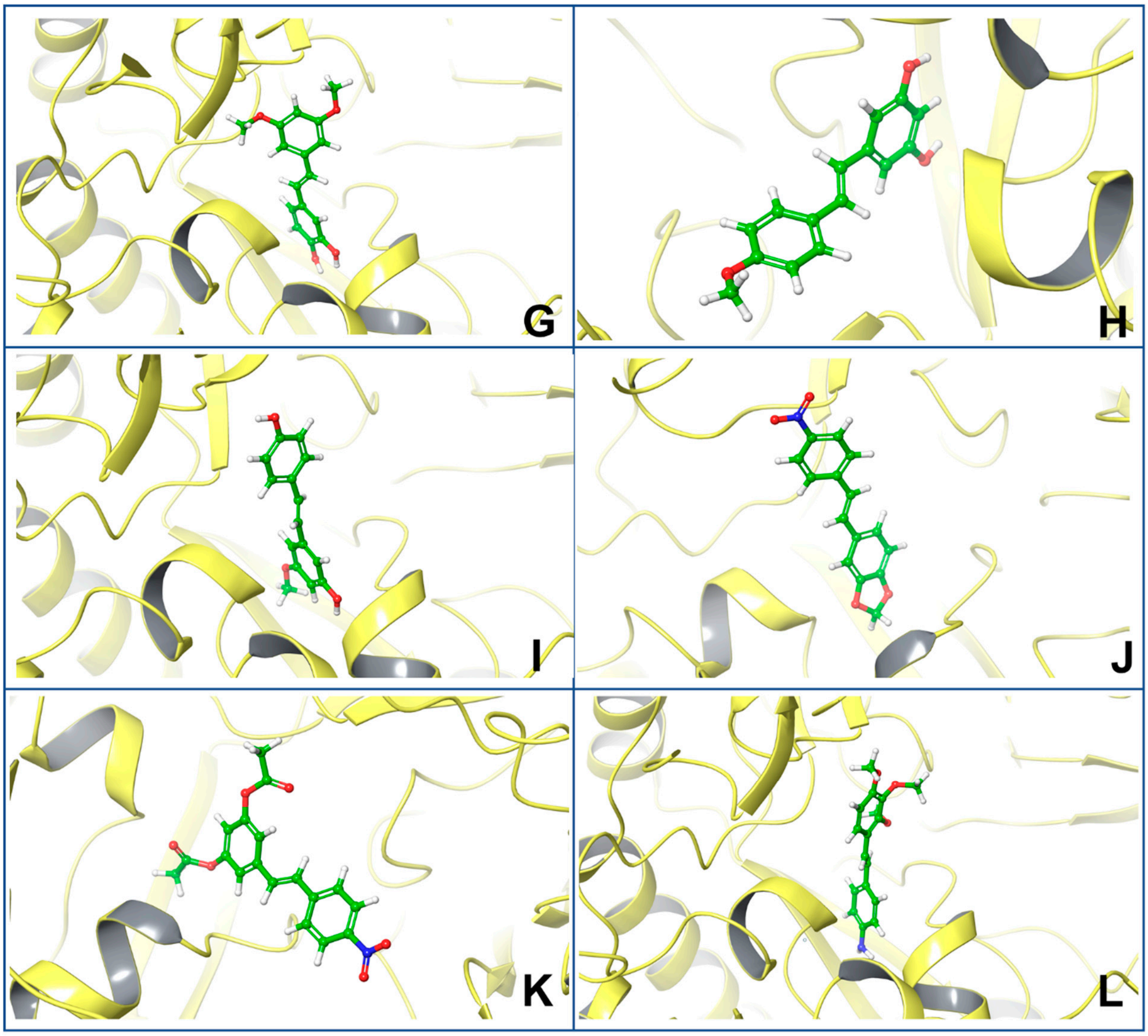

Figure S43 Docking poses of C site on 5TUS and six ligands: HPSB (G), DRG (H), Pinostilbene (I), Compound-1 (J), Compound-2 (K), and Compound-3 (L).

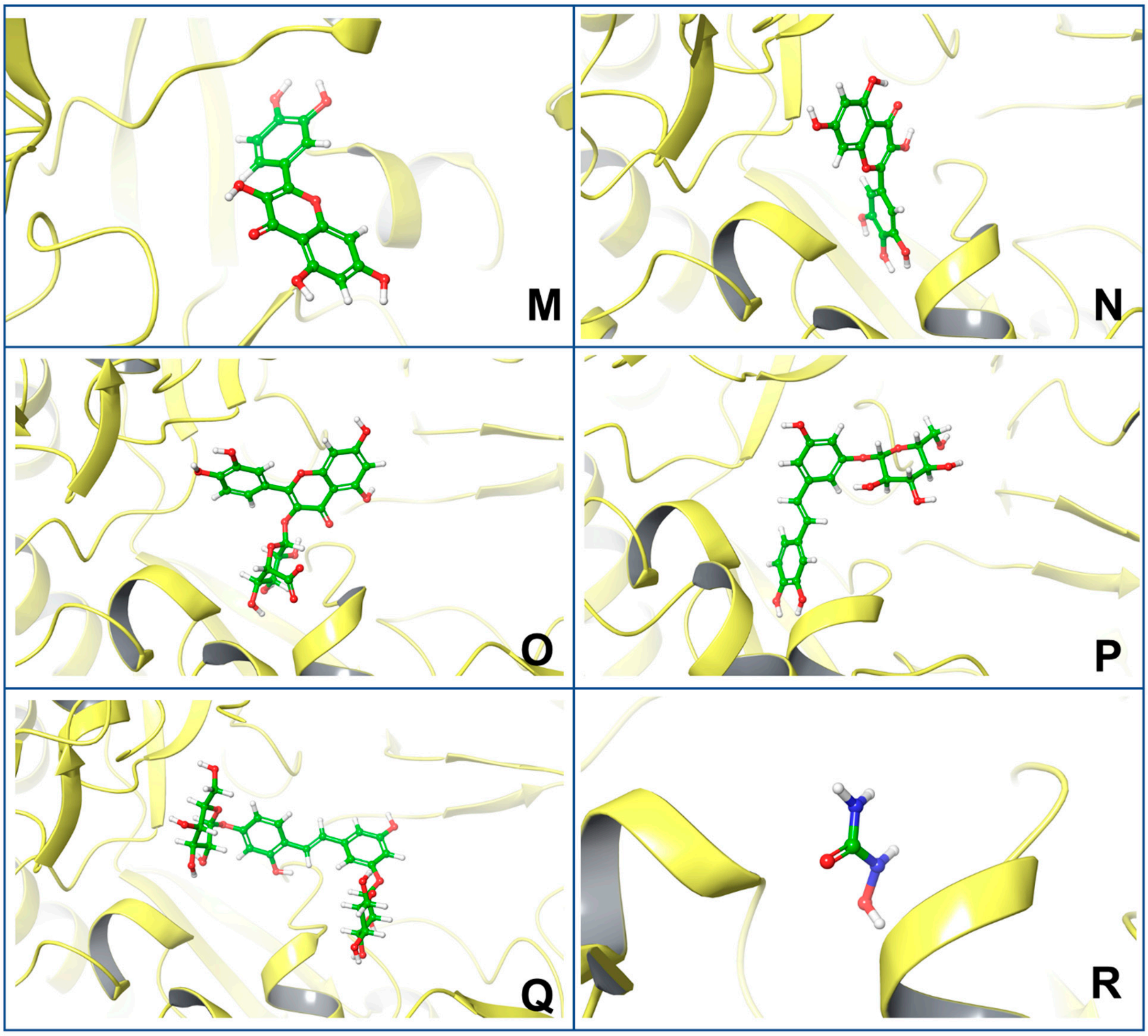

Figure S44 Docking poses of C site on 5TUS and six ligands: Quercetin (M), Myricetin (N), Astringin (O), Miquelianin (P), Mulberroside A (Q), and Hydroxyurea (R).
